# EDNRA Forms a Positive Feedback Loop with the Hippo/YAP Axis to Drive Triple‐Negative Breast Cancer Progression

**DOI:** 10.1002/advs.76784

**Published:** 2026-07-24

**Authors:** Zehao Hong, Boyang Li, Jiahui Xu, Ruonan Lin, Jinghao Pan, Chenlu Fang, Boxiang Zhang, Lucy Yue Lau, Richard J. Aldridge, Elizabeth A. Whitmore, Yi Chen

**Affiliations:** ^1^ Department of Breast Surgery The First Affiliated Hospital of Zhengzhou University Zhengzhou China; ^2^ Cancer Research Institute The Affiliated Cancer Hospital of Xinjiang Medical University Urumqi Xinjiang Uygur Autonomous Region China; ^3^ Department of Public Health Harvard Medical School Boston Massachusetts USA; ^4^ Department of Medicine University of Minnesota Medical School Minneapolis Minnesota USA

**Keywords:** EDNRA, G protein‐coupled receptor, Hippo/YAP signaling, positive feedback loop, triple‐negative breast cancer

## Abstract

Sustained Hippo/Yes‐associated protein (YAP) activation drives triple‐negative breast cancer (TNBC), but druggable upstream regulators remain unclear. We investigated G protein‐coupled receptors (GPCRs) as membrane inputs for YAP activation. TCGA‐based Hippo/YAP signature analysis, marketed‐drug GPCR annotation, and siRNA screening using connective tissue growth factor (CTGF) identified endothelin receptor type A (EDNRA). EDNRA function was tested by knockdown, overexpression, endothelin‐1 (ET‐1) stimulation, and atrasentan blockade in TNBC cells and xenografts. YAP regulation was examined by immunoblotting, RT‐qPCR, TEAD reporter assays, subcellular localization, docking, co‐immunoprecipitation, mutagenesis, ChIP‐qPCR, and CRISPRi. EDNRA correlated with Hippo/YAP signatures, adverse clinical features, and poor outcome. EDNRA depletion or atrasentan suppressed proliferation, migration, stem‐like populations, and xenograft growth, whereas EDNRA overexpression or ET‐1 had opposite effects. Mechanistically, EDNRA reduced YAP Ser127 phosphorylation, promoted nuclear YAP accumulation, and enhanced TEAD transcription through Gαq/11–Rho/ROCK–LATS signaling. Mutating predicted EDNRA–Gαq/11 interface residues impaired YAP activation and tumor‐promoting activity. Reciprocally, YAP/TEAD4 enhanced EDNRA transcription through an enhancer‐associated region. Atrasentan also sensitized TNBC cells to paclitaxel and produced positive zero interaction potency (ZIP) synergy scores. EDNRA establishes a druggable positive feedback loop with Hippo/YAP signaling and represents a potential therapeutic vulnerability in YAP‐driven TNBC.

## Graphical abstract

Endothelin receptor type A (EDNRA) and the Hippo/YAP pathway form a self‐reinforcing loop that sustains triple‐negative breast cancer. EDNRA activates YAP through Gαq/11–Rho/ROCK–LATS signaling, while YAP/TEAD4 reciprocally drives EDNRA transcription. The clinically tested antagonist atrasentan disrupts this circuit, restrains tumor growth, and sensitizes cells to paclitaxel—revealing a druggable vulnerability in YAP‐active disease.

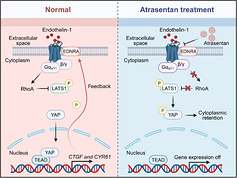

## 1 Introduction

Triple‐negative breast cancer (TNBC) is defined by the absence of estrogen receptor, progesterone receptor, and human epidermal growth factor receptor 2 (HER2) expression and represents one of the most aggressive molecular subtypes of breast cancer. TNBC is characterized by marked molecular heterogeneity, high metastatic potential, early recurrence, and limited durable targeted therapeutic options [1, 2]. Although immune checkpoint blockade has improved outcomes in early‐stage TNBC, recurrence and therapeutic resistance remain major clinical barriers [3]. Poly(ADP‐ribose) polymerase (PARP) inhibition in germline BRCA‐mutated disease and antibody–drug conjugates have further expanded treatment choices for selected patients, but durable disease control is still not achieved in a substantial proportion of cases [4, 5]. More recent clinical analyses continue to support the therapeutic value of antibody–drug conjugates in advanced TNBC, while also emphasizing the need for additional biomarker‐defined strategies [6, 7].

The Hippo/Yes‐associated protein (YAP) pathway is an evolutionarily conserved signaling cascade that controls tissue growth, regeneration, cell fate, and tumorigenesis. In the canonical pathway, mammalian STE20‐like protein kinase 1/2 (MST1/2) and large tumor suppressor kinase 1/2 (LATS1/2) phosphorylate YAP and transcriptional coactivator with PDZ‐binding motif (TAZ), resulting in cytoplasmic retention, ubiquitin‐mediated degradation, and transcriptional inactivation. When Hippo signaling is suppressed, YAP/TAZ translocate into the nucleus and cooperate with the TEA domain transcription factor (TEAD) family members to activate genes involved in proliferation, migration, stemness, extracellular matrix remodeling, immune regulation, and survival [8, 9]. In breast cancer, YAP/TAZ activity has been linked to aggressive phenotypes, metastatic plasticity, immune remodeling, and therapeutic resistance, particularly in TNBC‐like states [10, 11]. However, the upstream signals that sustain persistent YAP activation in TNBC remain incompletely defined.

G protein‐coupled receptors (GPCRs) are major cell‐surface sensors that convert extracellular cues into intracellular signaling outputs. The biological connection between GPCR signaling and Hippo/YAP regulation provides a framework for understanding how membrane‐level stimuli influence nuclear transcriptional programs [12, 13]. Depending on the coupled G‐protein subtype, GPCRs can either activate or suppress YAP/TAZ activity. Gαq/11‐ and Gα12/13‐coupled receptors generally promote YAP activation through Rho‐mediated inhibition of LATS kinases, whereas Gαs‐coupled receptors often suppress YAP through cyclic adenosine monophosphate (cAMP)/protein kinase A (PKA)‐related mechanisms. Because GPCRs are pharmacologically tractable membrane proteins and constitute one of the most successful families of approved drug targets, identifying GPCRs that functionally regulate Hippo/YAP signaling may provide an alternative strategy to direct inhibition of nuclear YAP/TEAD activity [14, 15].

Endothelin receptor type A (EDNRA), also known as endothelin A receptor, is a GPCR activated by endothelin‐1 (ET‐1). The endothelin system participates in tumor–microenvironment communication, vascular regulation, stromal remodeling, and malignant progression [16, 17]. Importantly, endothelin signaling has been connected to YAP‐associated oncogenic programs in colorectal and ovarian cancer, suggesting that endothelin receptors can function as membrane‐level inputs into YAP/TAZ signaling [18, 19]. Recent pan‐cancer analyses further suggest that EDNRA may be associated with tumor immune microenvironment features and immunotherapy‐related outcomes [20, 21]. Nevertheless, whether EDNRA regulates Hippo/YAP activity in TNBC, whether this regulation contributes to TNBC progression, and whether EDNRA itself is transcriptionally controlled by YAP remain unclear.

In the present study, we identified EDNRA as a druggable GPCR associated with sustained Hippo/YAP activation and aggressive TNBC phenotypes. Through integrated transcriptomic analysis, marketed‐drug‐targeted GPCR annotation, and small interfering RNA (siRNA) screening using connective tissue growth factor (CTGF) as a YAP activity readout, we identified EDNRA as a candidate upstream regulator of the Hippo/YAP pathway. We further evaluated EDNRA depletion, EDNRA overexpression, ET‐1 stimulation, and atrasentan‐mediated EDNRA blockade in TNBC models. Mechanistically, EDNRA activates YAP through the Gαq/11–Rho/ROCK (Rho‐associated coiled‐coil‐containing protein kinase)–LATS cascade, while YAP/TEAD family member 4 (TEAD4) reinforces EDNRA transcription through an enhancer‐associated regulatory region. These findings reveal an EDNRA–YAP positive feedback loop that contributes to malignant TNBC phenotypes, at least partly through YAP signaling, and suggest that EDNRA blockade may represent a therapeutic strategy for EDNRA‐expressing, YAP‐active TNBC.

## 2 Materials and Methods

### 2.1 Cell Culture

Human TNBC cell lines BT549, MDA‐MB‐231, MDA‐MB‐157, HCC1806, Hs578T, SUM149PT, MDA‐MB‐436, CAL‐51, MDA‐MB‐468, and HCC1937 were used for basal EDNRA expression screening. MDA‐MB‐231, BT549, and HCC1806 cells were used for subsequent functional validation. HEK293T cells were used for lentiviral packaging. MDA‐MB‐231 cells were cultured in Leibovitz's L‐15 medium supplemented with 10% fetal bovine serum (FBS) under atmospheric air. BT549 and HCC1806 cells were cultured in RPMI‐1640 medium supplemented with 10% FBS, and insulin was additionally supplemented for BT549 cells. HEK293T cells were cultured in high‐glucose Dulbecco's modified Eagle medium (DMEM) supplemented with 10% FBS at 37°C with 5% CO_2_. The remaining TNBC cell lines used for baseline screening were cultured according to the suppliers’ recommendations. All media were supplemented with 1% penicillin/streptomycin. All cell lines were authenticated by short tandem repeat profiling and routinely tested for mycoplasma contamination.

### 2.2 Public Dataset Analysis and GPCR Screening

RNA‐sequencing and clinicopathological data from The Cancer Genome Atlas (TCGA)‐BRCA were used for initial breast cancer‐level GPCR screening, EDNRA expression analysis, clinicopathological comparison, survival analysis, functional signature correlation, Hippo/YAP signature enrichment, and YAP target‐gene correlation. Genotype‐Tissue Expression (GTEx) normal breast tissue data were integrated with TCGA‐BRCA for tumor‐versus‐normal expression comparison. TNBC samples were identified according to available estrogen receptor, progesterone receptor, and HER2 annotations. TCGA‐TNBC was used for TNBC‐restricted validation of candidate GPCRs, functional signature analysis, Hippo/YAP enrichment, YAP target‐gene correlation, and survival analysis. Molecular Taxonomy of Breast Cancer International Consortium (METABRIC)‐TNBC and GSE58812 TNBC cohorts were used as independent validation datasets for EDNRA prognostic analysis. GSE9893_BRCA was used for the comparison of EDNRA expression according to distant metastasis status. TCGA‐COAD, TCGA‐LIHC, TCGA‐LUAD, and TCGA‐PAAD were used to evaluate the association between EDNRA expression and Hippo/YAP‐related signatures across additional cancer types. YAP and TEAD4 chromatin immunoprecipitation sequencing datasets GSE61852 and GSE131687 were used to analyze chromatin occupancy around the EDNRA locus.

Clinical endpoints were defined as follows. Overall survival (OS) was defined as the time from diagnosis or enrollment to death from any cause. Progression‐free interval (PFI) was defined as the time to first disease progression, recurrence, new tumor event, or death. Disease‐free interval (DFI) was defined as the time from disease‐free status after initial treatment to recurrence, new tumor event, or death. Relapse‐free survival (RFS) was defined as the time to tumor relapse or recurrence, with censoring at the last follow‐up for event‐free patients. Endpoint information was used as provided by the corresponding public dataset.

The initial GPCR gene universe was obtained from the MSigDB C5 Gene Ontology Molecular Function gene set GOMF_G_PROTEIN_COUPLED_RECEPTOR_ACTIVITY. GPCRs with available expression data in TCGA‐BRCA were retained. For each GPCR, Spearman correlation analysis was performed between its expression and the enrichment scores of two Hippo/YAP‐related gene sets, CORDENONSI_YAP_CONSERVED_SIGNATURE and REACTOME_SIGNALING_BY_HIPPO. GPCRs positively correlated with both Hippo/YAP‐related gene sets, using Spearman's ρ > 0.3 and P < 0.05 as the filtering criteria, were retained and intersected with a curated list of GPCRs targeted by marketed drugs. The resulting candidate GPCRs were further functionally screened by siRNA‐mediated knockdown in MDA‐MB‐231 cells using CTGF expression as a YAP activity readout. For TNBC‐specific validation, the 15 candidates were re‐evaluated in TCGA‐TNBC by gene set enrichment analysis and correlation analysis with combined Hippo/YAP signature z‐scores [14].

### 2.3 GPCR‐Based siRNA Screening

Candidate GPCRs were further screened in MDA‐MB‐231 cells. Cells were transfected with siRNAs targeting the candidate GPCRs. Forty‐eight hours after transfection, total RNA was extracted and reverse‐transcribed into complementary DNA (cDNA). CTGF, a classical YAP target gene, was measured by reverse transcription quantitative polymerase chain reaction (RT‐qPCR) as a readout of Hippo/YAP activity. CTGF expression in siControl cells was normalized to 1. The siGPCR sequences used for screening are listed in Table S1.

### 2.4 Gene Set Enrichment and Functional Signature Analysis

Gene set enrichment analysis (GSEA) was performed using GSEA software. Samples were divided into EDNRA‐high and EDNRA‐low groups according to the median EDNRA expression level unless otherwise indicated. Functional signatures, including angiogenesis, apoptosis, cell cycle, differentiation, DNA damage, DNA repair, epithelial–mesenchymal transition (EMT), hypoxia, inflammation, invasion, metastasis, proliferation, quiescence, and stemness, were scored using predefined gene sets. Correlations between EDNRA expression and functional signatures were calculated using Spearman correlation analysis.

### 2.5 Clinical Specimens

Human TNBC tissues and normal breast tissues were obtained from the Affiliated Cancer Hospital of Xinjiang Medical University. This study was approved by the Ethics Committee of the Affiliated Cancer Hospital of Xinjiang Medical University (approval no. K‐2024014). Written informed consent was obtained from all participants or their legal guardians. Clinical information was anonymized before analysis.

### 2.6 DNA Constructs and Mutagenesis

Flag‐EDNRA, Myc‐YAP, LATS1, kinase‐defective LATS1‐K/R, Myc‐Rho‐L63, TEAD luciferase reporter, Renilla luciferase control, catalytically inactive Cas9–Krüppel‐associated box (dCas9‐KRAB), and single‐guide RNA (sgRNA) expression vectors were used in this study. EDNRA mutants, including Thr28A, Trp154A, His66A, and the combined Thr28A/Trp154A/His66A mutant, were generated by site‐directed mutagenesis and verified by sequencing.

### 2.7 Transfection and Lentiviral Infection

Plasmid transfection was performed using Lipofectamine 2000 according to the manufacturer's instructions. siRNA transfection was performed using RNAiMAX. For short‐term in vitro assays, siRNA‐mediated knockdown was used to achieve transient EDNRA depletion and to minimize potential effects caused by long‐term selection or adaptation. Cells were collected at the indicated time points for RT‐qPCR, western blotting, luciferase assays, or functional experiments.

For stable EDNRA knockdown, shEDNRA or shControl sequences were cloned into a lentiviral vector. Lentiviruses were produced in HEK293T cells using packaging plasmids. Viral supernatants were collected, filtered, and used to infect MDA‐MB‐231 cells. Stable cells were selected with puromycin before xenograft experiments. The shRNA system was used for in vivo experiments to maintain sustained EDNRA depletion during tumor growth. The knockdown efficiency of siRNA‐ and shRNA‐mediated EDNRA depletion was compared by RT‐qPCR before functional validation. siRNA, short hairpin RNA (shRNA), sgRNA, and other oligonucleotide sequences are listed in Table S2.

### 2.8 CRISPR Interference (CRISPRi) Assay

CRISPRi was used to repress candidate YAP/TEAD4‐bound regulatory regions around the EDNRA locus. MDA‐MB‐231 cells stably expressing dCas9‐KRAB were transduced with sgRNAs targeting candidate promoter‐ or enhancer‐associated regions identified from YAP and TEAD4 ChIP‐seq signals. A non‐targeting sgRNA was used as the control. After selection, EDNRA expression was examined by RT‐qPCR, and YAP enrichment at the targeted regions was analyzed by chromatin immunoprecipitation quantitative PCR (ChIP‐qPCR). sgRNA sequences and target coordinates are listed in Table S2.

### 2.9 Pharmacological Treatments

Atrasentan was used to inhibit EDNRA signaling, and endothelin‐1 (ET‐1) was used to activate EDNRA signaling. Paclitaxel (PTX) was used for chemotherapy‐sensitivity and drug‐combination assays. For combination experiments, PTX was administered alone or together with a fixed low dose of atrasentan at 300 nM unless otherwise indicated. Verteporfin and XMU‐MP‐1 were used to inhibit or activate YAP pathway output. C3 transferase was used to inhibit Rho signaling. GSK429286 and Y27632 were used as ROCK inhibitors. Cells were treated with the indicated reagents at the concentrations and durations shown in the figures. Chemicals and treatment conditions are listed in Table S3.

### 2.10 Drug‐Combination and Synergy Analysis

For drug‐combination assays, TNBC cells were treated with increasing concentrations of paclitaxel (PTX) and atrasentan either alone or in combination. For IC50 comparison, cells were treated with increasing concentrations of PTX in the presence or absence of a fixed low dose of atrasentan at 300 nM. Cell viability was measured using the Cell Counting Kit‐8 assay, and IC50 values were calculated from dose–response curves. For drug‐matrix analysis, PTX and atrasentan were combined across the indicated concentration ranges, and inhibition rates were calculated relative to untreated controls. Drug synergy was first evaluated using the zero interaction potency (ZIP) model [22, 23]. ZIP synergy scores greater than 10 were interpreted as synergistic interactions between PTX and atrasentan. In addition, a combination index (CI) analysis was performed according to the Chou–Talalay method using CalcuSyn software based on the indicated fixed‐ratio PTX–atrasentan combination data. CI values less than 1 were interpreted as synergism, CI values equal to 1 as additive effects, and CI values greater than 1 as antagonism.

### 2.11 RNA Isolation and Reverse Transcription Quantitative PCR (RT‐qPCR)

Total RNA was extracted from cells using an RNA extraction reagent according to the manufacturer's protocol. RNA was reverse‐transcribed into cDNA, and RT‐qPCR was performed using SYBR Green‐based detection on a real‐time PCR system. Relative mRNA expression was calculated using the 2−ΔΔCt method and normalized to the internal control gene. Primer sequences for RT‐qPCR are listed in Table S4.

### 2.12 Immunohistochemistry (IHC)

Formalin‐fixed paraffin‐embedded tissue sections were deparaffinized, rehydrated, subjected to antigen retrieval, and blocked. Sections were incubated with primary antibodies against EDNRA, YAP, or Ki‐67, followed by secondary antibody incubation. Signals were developed with 3,3′‐diaminobenzidine (DAB), and nuclei were counterstained with hematoxylin. Staining was evaluated independently by investigators blinded to clinicopathological information. The staining index was calculated by multiplying the staining intensity score by the positive‐cell proportion score. Antibodies are listed in Table S5.

### 2.13 Cell Counting Kit‐8 (CCK‐8) Assay

Cell viability was measured using Cell Counting Kit‐8 (CCK‐8). Cells were seeded into 96‐well plates after the indicated transfection or treatment. CCK‐8 reagent was added at the indicated time points, and absorbance at 450 nm was measured using a microplate reader.

### 2.14 5‐Ethynyl‐2′‐Deoxyuridine (EdU) Incorporation Assay

Cells were incubated with EdU reagent after the indicated treatments. After fixation and permeabilization, EdU staining was performed according to the manufacturer's protocol. Nuclei were counterstained with 4′,6‐diamidino‐2‐phenylindole (DAPI) or Hoechst 33342, as indicated. Images were acquired by fluorescence microscopy, and EdU‐positive cells were quantified from randomly selected fields.

### 2.15 Transwell Migration Assay

Cell migration was evaluated using Transwell chambers with 8‐µm pores. Cells suspended in serum‐free medium were seeded into the upper chamber, and medium containing serum was added to the lower chamber. After incubation, cells remaining on the upper surface were removed. Migrated cells on the lower membrane surface were fixed, stained, photographed, and counted from randomly selected fields.

### 2.16 Wound‐Healing Assay

Cells were grown to near confluence, and a linear wound was generated using a sterile pipette tip. Detached cells were removed by washing. Images were captured at 0 h and at the indicated time point. Wound closure was calculated as [1 − wound width at the indicated time/wound width at 0 h] × 100%.

### 2.17 Colony Formation Assay

Cells were seeded into culture plates at low density after plasmid transfection. After incubation, colonies were fixed, stained, photographed, and counted. Colonies were quantified from independent wells as indicated in the figure legends.

### 2.18 Apoptosis Assay

Apoptosis was analyzed by flow cytometry using Annexin V and propidium iodide or an equivalent apoptosis detection kit. Cells were harvested after the indicated treatments, washed, stained according to the manufacturer's protocol, and analyzed using a flow cytometer. Early and late apoptotic cells were included in the total apoptotic population.

### 2.19 CD24/CD44 Flow Cytometry

Cells were collected after the indicated treatments and stained with fluorophore‐conjugated antibodies against CD44 and CD24. Single viable cells were first selected according to forward and side scatter profiles, followed by doublet exclusion. Gates were established using unstained, single‐stained, and fluorescence‐minus‐one controls. The CD44^high/CD24^low/− fraction was quantified as the CD24/CD44‐defined stem‐like population. The percentage of this population was calculated among live single cells. Antibodies are listed in Table S5.

### 2.20 Western Blotting

Cells or tumor tissues were lysed with western and immunoprecipitation lysis buffer supplemented with protease and phosphatase inhibitors. Protein concentration was measured, and equal amounts of protein were separated by sodium dodecyl sulfate–polyacrylamide gel electrophoresis (SDS‐PAGE) and transferred onto polyvinylidene difluoride (PVDF) membranes. Membranes were blocked, incubated with primary antibodies overnight at 4°C, and then incubated with horseradish peroxidase (HRP)‐conjugated secondary antibodies. Signals were visualized using enhanced chemiluminescence. β‐Actin was used as the loading control for whole‐cell lysates. Antibodies are listed in Table S5. Immunoblot band intensities were quantified using ImageJ software. Total protein levels were normalized to β‐Actin. Phosphorylated protein levels were normalized to their corresponding total protein levels where applicable. For nuclear and cytoplasmic fractionation experiments, nuclear and cytoplasmic YAP signals were normalized to Histone H3 and α‐Tubulin, respectively. Quantified values were expressed relative to the indicated control group.

### 2.21 Nuclear and Cytoplasmic Fractionation

Nuclear and cytoplasmic proteins were extracted using a nuclear/cytoplasmic fractionation kit according to the manufacturer's protocol. α‐Tubulin and Histone H3 were used as cytoplasmic and nuclear markers, respectively. YAP localization in each fraction was analyzed by western blotting.

### 2.22 Co‐Immunoprecipitation

For co‐immunoprecipitation, cell lysates were precleared with protein A/G beads and control immunoglobulin G (IgG). Immunoprecipitation was performed using the indicated antibodies or tag‐affinity beads at 4°C. Immune complexes were washed, eluted, and analyzed by western blotting. Co‐immunoprecipitation was used to assess EDNRA–Gαq/11 interaction and LATS1‐associated YAP or phosphorylated YAP (p‐YAP) signals. Antibodies are listed in Table S5.

### 2.23 TEAD Luciferase Reporter Assay

Cells were co‐transfected with a TEAD‐responsive firefly luciferase reporter and a Renilla luciferase control plasmid. After the indicated genetic manipulation or drug treatment, luciferase activity was measured using a dual‐luciferase reporter system. Firefly luciferase activity was normalized to Renilla luciferase activity.

### 2.24 Immunofluorescence Staining

Cells grown on coverslips were fixed with 4% paraformaldehyde, permeabilized, and blocked. Cells were incubated with primary antibodies against YAP or EDNRA, followed by fluorophore‐conjugated secondary antibodies. Nuclei were counterstained with DAPI. Negative controls were incubated with secondary antibodies alone. Images were acquired by fluorescence or confocal microscopy. YAP localization was quantified by the proportion of cells with nuclear YAP staining or by the nuclear/cytoplasmic fluorescence intensity ratio. Antibodies are listed in Table S5.

### 2.25 RNA Sequencing (RNA‐seq) and Data Analysis

Total RNA was extracted from siControl and siEDNRA MDA‐MB‐231 cells. RNA quality and integrity were assessed before library construction and high‐throughput RNA sequencing. The processed gene expression matrix generated from RNA‐seq data was used for downstream analysis. Differentially expressed genes between siEDNRA and siControl cells were identified using an adjusted P‐value < 0.05 and an absolute log2 fold change > 1 as the cutoff. Adjusted P‐values were calculated using the Benjamini–Hochberg method. Volcano plots, heatmaps, Gene Ontology (GO) enrichment analysis, and Kyoto Encyclopedia of Genes and Genomes (KEGG) pathway enrichment analysis were performed using R‐based tools.

### 2.26 Chromatin Immunoprecipitation Sequencing (ChIP‐seq) Analysis

YAP and TEAD4 chromatin immunoprecipitation sequencing (ChIP‐seq) data were retrieved from Gene Expression Omnibus (GEO) datasets GSE61852 and GSE131687. Normalized signal tracks in bigWig (bw) format aligned to the human reference genome GRCh38/hg38 were directly downloaded. ChIP‐seq signals around the EDNRA locus were visualized using genome browser software to evaluate YAP and TEAD4 chromatin occupancy.

### 2.27 Chromatin Immunoprecipitation Quantitative PCR (ChIP‐qPCR)

Cells were crosslinked with formaldehyde and quenched. Chromatin was fragmented by sonication and immunoprecipitated with anti‐YAP antibody or control IgG. After reverse crosslinking and DNA purification, enrichment at candidate EDNRA regulatory regions was measured by quantitative PCR (qPCR) and normalized to input DNA.

### 2.28 Molecular Docking

Protein structures of EDNRA and GNAQ were obtained from public structural databases or predicted using validated modeling tools. Molecular docking was performed to predict EDNRA–Gαq interaction interfaces. Candidate binding residues were visualized using molecular graphics software and selected for mutagenesis.

### 2.29 Xenograft Tumor Assay

All animal experiments were approved by the Animal Ethics Committee of Xinjiang Medical University (ethics no. A240301‐194). Female immunodeficient mice were maintained under specific pathogen‐free conditions with controlled temperature, humidity, light/dark cycle, and free access to food and water.

For the genetic knockdown model, MDA‐MB‐231 cells stably expressing shControl or shEDNRA were subcutaneously injected into the flank region. For pharmacological and ligand‐stimulation experiments, MDA‐MB‐231 xenograft‐bearing mice were treated with vehicle, atrasentan, ET‐1, or the indicated intervention. Tumor length and width were measured at the indicated time points, and tumor volume was calculated as length × width^2^/2. At the endpoint, mice were humanely euthanized. Tumors were collected, photographed, weighed, fixed, and subjected to histological and immunohistochemical analyses.

### 2.30 Statistical Analysis

Statistical analyses were performed using GraphPad Prism and R software. Data are presented as mean ± standard deviation (SD) unless otherwise indicated. For experimental assays, two‐group comparisons were analyzed using two‐tailed unpaired Student's *t*‐test, and comparisons among three or more groups were analyzed using one‐way analysis of variance (ANOVA) followed by Tukey's or Dunnett's post hoc test, as appropriate. Tumor growth curves were analyzed using two‐way ANOVA. Correlations were assessed using Spearman correlation analysis unless otherwise indicated. Categorical variables were analyzed using the χ^2^ test or Fisher's exact test. Survival curves were generated using the Kaplan–Meier method and compared using the log‐rank test. Statistical significance was defined as *p* < 0.05.

## 3 Results

### 3.1 EDNRA Is Identified as a Candidate Regulator of the Hippo/YAP Pathway in TNBC

**FIGURE 1:**
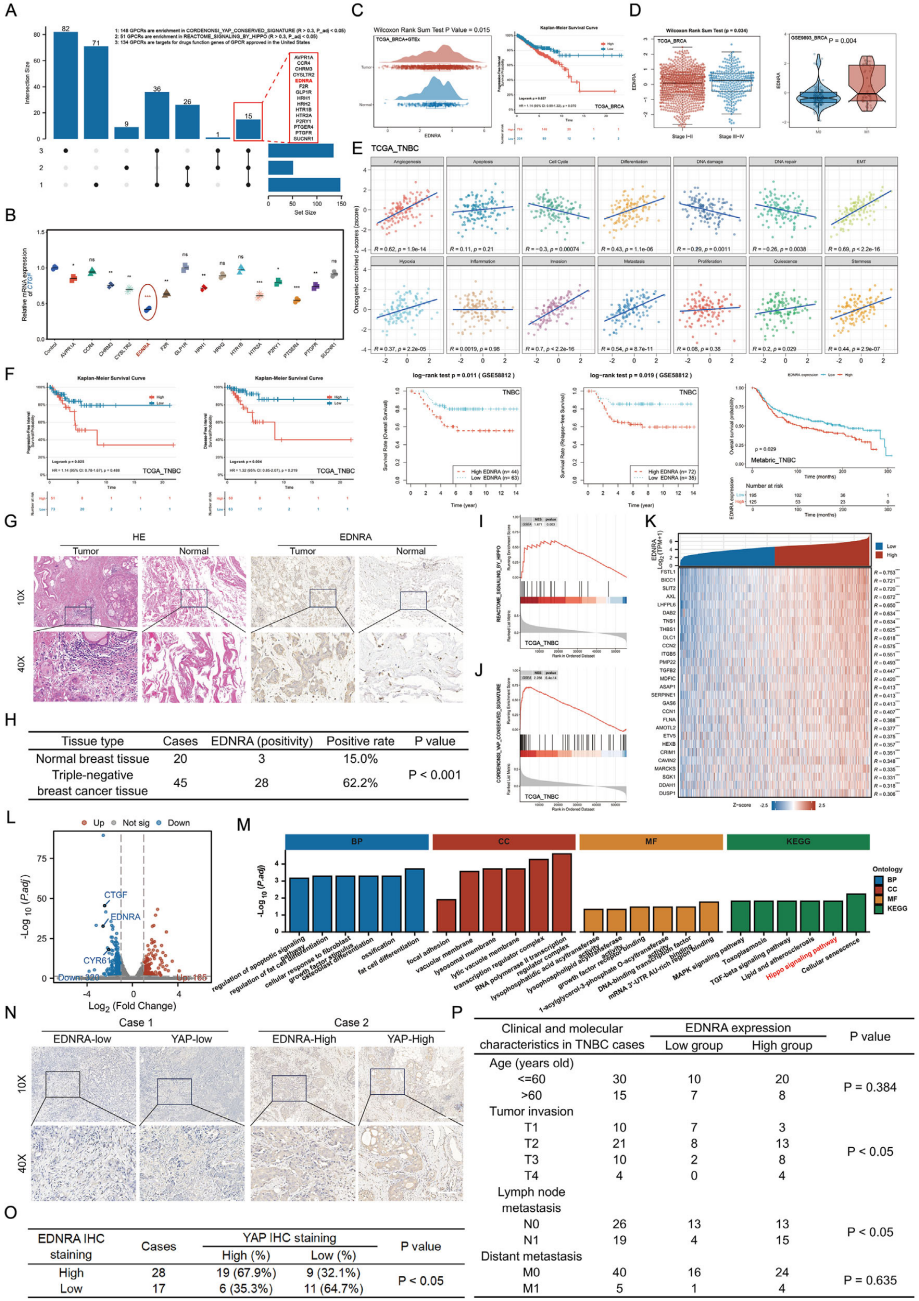
EDNRA is identified as a candidate G protein‐coupled receptor (GPCR) regulator associated with Hippo/YAP signaling and triple‐negative breast cancer (TNBC) progression. (A) UpSet plot showing the intersection of GPCRs whose expression correlated with the CORDENONSI_YAP_CONSERVED_SIGNATURE gene set, GPCRs whose expression correlated with the REACTOME_SIGNALING_BY_HIPPO gene set, and GPCRs targeted by marketed drugs. Fifteen overlapping candidate GPCRs were identified. (B) Reverse transcription quantitative polymerase chain reaction (RT‐qPCR) analysis of CTGF mRNA expression after siRNA‐mediated depletion of the 15 candidate GPCRs in MDA‐MB‐231 cells. CTGF was used as a transcriptional readout of Hippo/YAP pathway activity. (C) EDNRA expression in breast cancer tissues and normal breast tissues based on TCGA‐BRCA and GTEx datasets, together with Kaplan–Meier analysis of overall survival in TCGA‐BRCA patients stratified by EDNRA expression. (D) EDNRA expression in TCGA‐BRCA samples stratified by tumor stage and in GSE9893_BRCA samples stratified by distant metastasis status. (E) Correlation analysis between EDNRA expression and functional signatures in TCGA‐TNBC samples. (F) Kaplan–Meier survival analyses of TNBC patients stratified by EDNRA expression. The evaluated endpoints included progression‐free interval and disease‐free interval in TCGA‐TNBC, overall survival and relapse‐free survival in the GSE58812 TNBC cohort, and overall survival in the METABRIC‐TNBC cohort. (G) Representative hematoxylin and eosin staining and EDNRA immunohistochemistry staining in TNBC tissues and normal breast tissues. Images are shown at 10× and 40× magnification. (H) Quantification of EDNRA positivity in normal breast tissues and TNBC tissues from Xinjiang Medical University Cancer Hospital. (I, J) Gene set enrichment analysis plots showing enrichment of Hippo/YAP‐related gene sets in EDNRA‐high versus EDNRA‐low TCGA‐TNBC samples. (K) Heatmap showing the association between EDNRA expression and YAP target genes in TCGA‐BRCA samples. (L) Volcano plot showing differentially expressed genes in siEDNRA versus siControl MDA‐MB‐231 cells. EDNRA, CTGF, and CYR61 are highlighted. (M) Gene Ontology and Kyoto Encyclopedia of Genes and Genomes enrichment analysis of differentially expressed genes after EDNRA depletion. (N) Representative immunohistochemistry staining of EDNRA and YAP in TNBC samples with low or high EDNRA/YAP expression. Images are shown at 10× and 40× magnification. (O) Association between EDNRA immunohistochemistry staining and YAP immunohistochemistry staining in TNBC samples. (P) Association between EDNRA expression and clinicopathological characteristics in TNBC patients from Xinjiang Medical University Cancer Hospital. Data are presented as the mean ± standard deviation where applicable. ns, not significant; ^*^ *p* < 0.05; ^**^ *p* < 0.01; ^***^ *p* < 0.001.

To identify druggable upstream regulators of Hippo/YAP signaling in breast cancer, we first performed an integrated GPCR‐focused screening analysis using TCGA‐BRCA transcriptomic data. GPCRs whose expression was positively associated with both the CORDENONSI_YAP_CONSERVED_SIGNATURE and REACTOME_SIGNALING_BY_HIPPO gene sets were intersected with GPCRs targeted by marketed drugs. This strategy identified 15 candidate druggable GPCRs, including EDNRA, AVPR1A, CCR4, CHRM3, CYSLTR2, F2R, GLP1R, HRH1, HRH2, HTR1B, HTR2A, P2RY1, PTGER4, PTGFR, and SUCNR1 (Figure 1A). To functionally prioritize these candidates, we individually silenced the 15 GPCRs in MDA‐MB‐231 TNBC cells and measured CTGF expression as a canonical transcriptional readout of YAP activity. Among these candidates, EDNRA knockdown caused the most pronounced decrease in CTGF expression, suggesting that EDNRA may act as a leading GPCR regulator of Hippo/YAP signaling in TNBC cells (Figure 1B). We next examined whether EDNRA showed clinical relevance in breast cancer at the pan‐breast cancer level. Analysis of TCGA‐BRCA combined with GTEx normal breast tissues showed that EDNRA expression was significantly higher in breast cancer tissues than in normal breast tissues. In TCGA‐BRCA, high EDNRA expression was also associated with poorer overall survival, supporting its potential clinical relevance in breast cancer (Figure 1C). Further clinicopathological analysis showed that EDNRA expression was increased in advanced‐stage tumors in TCGA‐BRCA and in M1 samples in the GSE9893_BRCA cohort, indicating that EDNRA expression may be associated with more aggressive breast cancer features and distant metastasis‐related status in breast cancer (Figure 1D).

Because the present study focuses on TNBC, and because Hippo/YAP signaling can display subtype‐dependent biological functions, we further re‐analyzed the candidate GPCRs specifically within the TCGA‐TNBC cohort. Using TNBC‐restricted samples, we evaluated the association between the 15 candidate GPCRs and Hippo/YAP activity by gene set enrichment analysis and correlation analysis with combined Hippo/YAP signature z‐scores. EDNRA remained one of the candidates meeting both selection criteria, with a normalized enrichment score greater than 2 and a correlation coefficient greater than 0.3, together with HRH1 and HTR2A (Figure S1A,B). These TNBC‐specific analyses confirmed that the prioritization of EDNRA was not merely driven by pan‐breast cancer heterogeneity, but remained evident within the TNBC subtype. In parallel, EDNRA‐high tumors showed enrichment of the CORDENONSI_YAP_CONSERVED_SIGNATURE and REACTOME_SIGNALING_BY_HIPPO gene sets in COAD, LIHC, LUAD, and PAAD cohorts, supporting a broader association between EDNRA expression and Hippo/YAP‐related signatures across additional cancer types (Figure S2A,B). We then assessed the biological and prognostic significance of EDNRA specifically in TNBC. In TCGA‐TNBC samples, EDNRA expression was positively correlated with malignant and microenvironment‐related functional signatures, including angiogenesis, epithelial–mesenchymal transition, hypoxia, invasion, metastasis, quiescence, and stemness, whereas negative correlations were observed with cell‐cycle, DNA damage, and DNA repair signatures (Figure 1E). Survival analyses further showed that high EDNRA expression was associated with unfavorable clinical outcomes in TNBC. Specifically, high EDNRA expression predicted shorter progression‐free interval and disease‐free interval in TCGA‐TNBC, poorer overall survival and relapse‐free survival in the GSE58812 TNBC cohort, and reduced overall survival in the METABRIC‐TNBC cohort (Figure 1F). These results indicate that EDNRA has prognostic relevance across independent TNBC datasets.

Consistent with these public dataset findings, immunohistochemical analysis of clinical specimens showed stronger EDNRA staining in TNBC tissues than in normal breast tissues, with a significantly higher EDNRA‐positive rate in TNBC samples (Figure 1G,H). Moreover, EDNRA‐high TNBC tumors were enriched for Hippo/YAP‐related gene sets, including REACTOME_SIGNALING_BY_HIPPO and CORDENONSI_YAP_CONSERVED_SIGNATURE (Figure 1I,J). EDNRA expression was also positively associated with multiple YAP target genes, including CTGF and CYR61 (Figure 1K). RNA sequencing after EDNRA depletion in MDA‐MB‐231 cells further showed downregulation of EDNRA, CTGF, and CYR61, together with enrichment of Hippo signaling‐related alterations (Figure 1L,M). Finally, EDNRA protein expression was positively associated with YAP expression in TNBC tissues and was significantly associated with tumor invasion and lymph node metastasis (Figure 1N–P). Collectively, these findings identify EDNRA as a druggable GPCR candidate with TNBC‐specific relevance to Hippo/YAP activity, malignant functional programs, and adverse clinical outcome.

### 3.2 EDNRA Depletion Suppresses TNBC Cell Growth, Migration, Stem‐Like Properties, and Tumorigenesis

**FIGURE 2:**
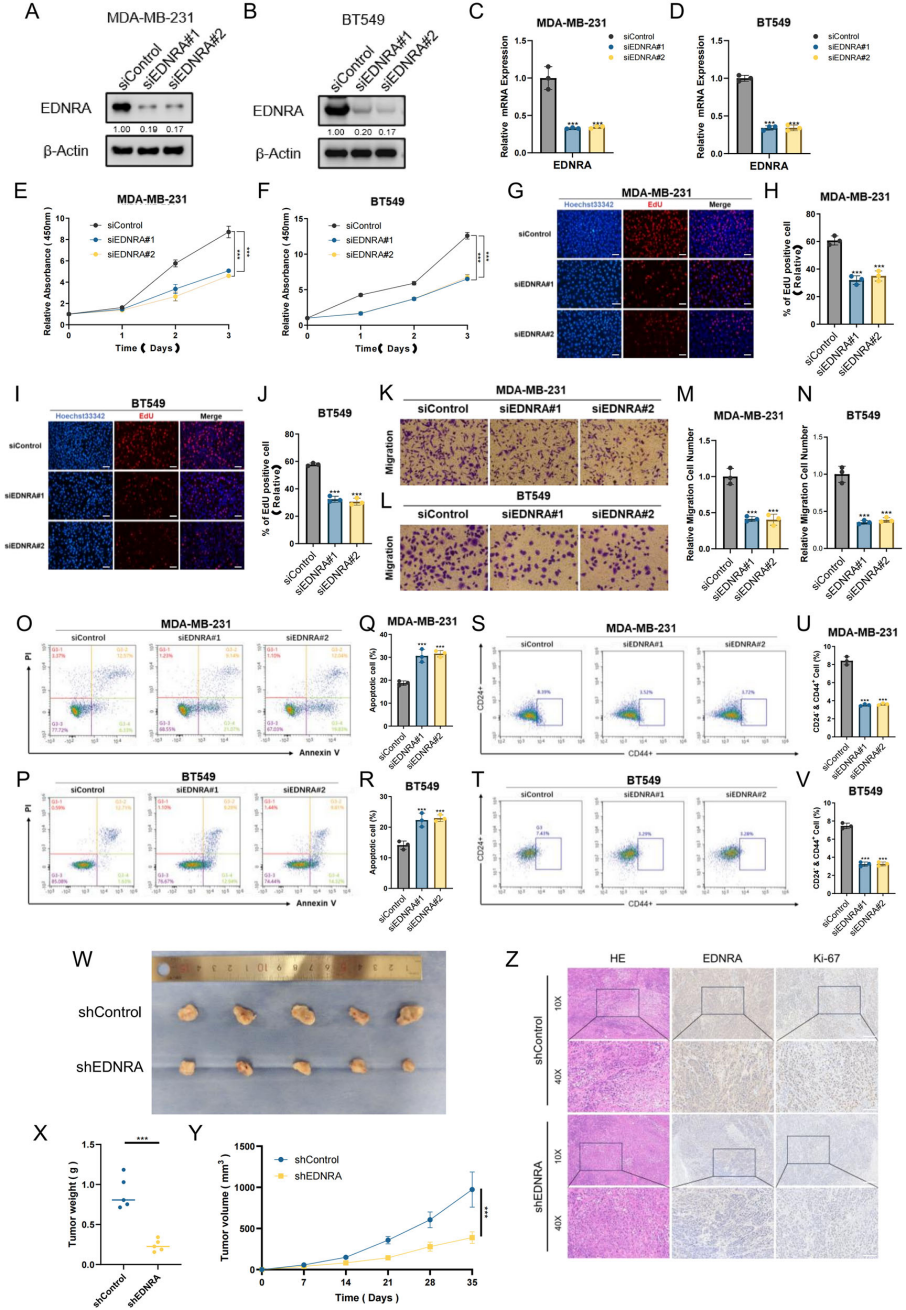
Loss of EDNRA inhibits TNBC cell growth, migration, stem‐like properties, and xenograft tumor formation. (A, B) Western blot analysis of EDNRA protein expression after siRNA‐mediated EDNRA knockdown in MDA‐MB‐231 and BT549 cells. β‐Actin was used as the loading control. (C, D) RT‐qPCR analysis of EDNRA mRNA expression in MDA‐MB‐231 and BT549 cells transfected with siControl or two independent siEDNRA sequences. (E, F) Cell Counting Kit‐8 (CCK‐8) assays showing the proliferation of MDA‐MB‐231 and BT549 cells after EDNRA knockdown. (G–J) Representative 5‐ethynyl‐2′‐deoxyuridine (EdU) staining images and quantification of EdU‐positive cells in MDA‐MB‐231 and BT549 cells transfected with siControl or siEDNRA (Hoechst 33342, blue; EdU, red). (K–N) Representative Transwell migration images and quantification of migrated MDA‐MB‐231 and BT549 cells after EDNRA depletion. (O–R) Flow cytometric analysis and quantification of apoptotic cells after EDNRA knockdown in MDA‐MB‐231 and BT549 cells. (S–V) Flow cytometric analysis and quantification of the CD44^high/CD24^low/− stem‐like population in MDA‐MB‐231 and BT549 cells following EDNRA knockdown. (W) Representative images of xenograft tumors derived from shControl and shEDNRA MDA‐MB‐231 cells. (X, Y) Quantification of tumor weight and tumor volume in the shControl and shEDNRA groups. (Z) Representative H&E, EDNRA, and Ki‐67 staining of xenograft tumor sections from the shControl and shEDNRA groups. Images are shown at 10× and 40× magnification. Data are presented as the mean ± standard deviation (SD). For xenograft experiments, n = 5 mice per group. ^**^ *p* < 0.01; ^***^ *p* < 0.001.

Before functional characterization, we examined basal EDNRA expression across a panel of TNBC cell lines. Western blotting and RT‐qPCR showed relatively high EDNRA expression in BT549, MDA‐MB‐231, and HCC1806 cells; therefore, MDA‐MB‐231 and BT549 cells were used for the main functional assays, and HCC1806 cells were included as an additional validation model (Figure S3A,B). We also confirmed efficient EDNRA depletion by both siRNA and shRNA approaches, with comparable knockdown efficiency between the transient and stable silencing systems (Figure S3C,D). We next examined whether EDNRA was functionally required for malignant progression in TNBC cells. Two independent siRNAs were used to deplete EDNRA in MDA‐MB‐231 and BT549 cells, and western blotting and RT‐qPCR confirmed efficient reduction of EDNRA protein and mRNA expression in both cell lines (Figure 2A–D). Functionally, EDNRA knockdown markedly impaired TNBC cell proliferation, as shown by reduced CCK‐8 absorbance and a decreased proportion of EdU‐positive proliferating cells in MDA‐MB‐231 and BT549 cells (Figure 2E–J). EDNRA depletion also significantly decreased Transwell migration and increased apoptotic cell populations, indicating that EDNRA supports the migratory and survival capacities of TNBC cells (Figure 2K–R). In addition, EDNRA knockdown reduced the CD44^high/CD24^low/− fraction, suggesting that EDNRA contributes to the maintenance of CD24/CD44‐defined stem‐like features in TNBC cells (Figure 2S–V). To further validate these findings in an additional TNBC model, we performed the same loss‐of‐function experiments in HCC1806 cells. Consistently, EDNRA knockdown reduced EDNRA protein expression, suppressed cell proliferation as indicated by CCK‐8 and EdU proliferation assays, impaired cell migration, and increased apoptosis in HCC1806 cells (Figure S4A–H). These results support that the pro‐malignant function of EDNRA is reproducible across multiple TNBC cell lines. To further assess the role of EDNRA in vivo, MDA‐MB‐231 cells stably expressing shControl or shEDNRA were injected into nude mice. EDNRA depletion significantly inhibited xenograft tumor growth, as shown by reduced tumor size, tumor weight, and tumor volume (Figure 2W–Y). Immunohistochemical staining confirmed decreased EDNRA expression and reduced Ki‐67 positivity in shEDNRA tumors (Figure 2Z). Together, these findings demonstrate that EDNRA depletion suppresses TNBC progression in vitro and in vivo.

### 3.3 Atrasentan‐Mediated Blockade of Ednra Restrains TNBC Progression

**FIGURE 3:**
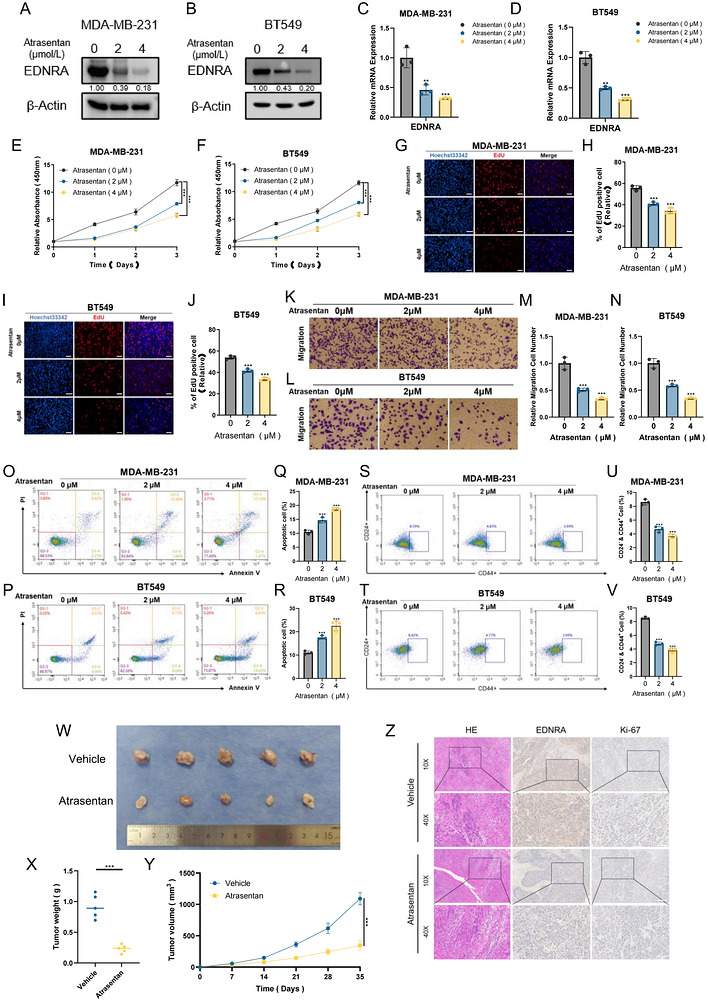
Atrasentan treatment inhibits EDNRA‐associated malignant phenotypes in TNBC cells and xenograft tumors. (A, B) Western blot analysis of EDNRA protein expression in MDA‐MB‐231 and BT549 cells treated with atrasentan at the indicated concentrations. β‐Actin was used as the loading control. (C, D) RT‐qPCR analysis of EDNRA mRNA expression in MDA‐MB‐231 and BT549 cells after atrasentan treatment. (E, F) CCK‐8 assays showing the proliferation of MDA‐MB‐231 and BT549 cells treated with atrasentan. (G–J) Representative EdU staining images and quantification of EdU‐positive cells after atrasentan treatment in MDA‐MB‐231 and BT549 cells (Hoechst 33342, blue; EdU, red). (K–N) Representative Transwell migration images and quantification of migrated MDA‐MB‐231 and BT549 cells following atrasentan treatment. (O–R) Flow cytometric analysis and quantification of apoptotic cells after atrasentan treatment in MDA‐MB‐231 and BT549 cells. (S–V) Flow cytometric analysis and quantification of the CD44^high/CD24^low/− stem‐like population in MDA‐MB‐231 and BT549 cells treated with atrasentan. (W) Representative images of xenograft tumors from the vehicle and atrasentan‐treated groups. (X, Y) Quantification of xenograft tumor weight and tumor volume in the vehicle and atrasentan‐treated groups. (Z) Representative H&E, EDNRA, and Ki‐67 staining of xenograft tumor sections from the vehicle and atrasentan‐treated groups. Images are shown at 10× and 40× magnification. Data are presented as the mean ± SD. For xenograft experiments, n = 5 mice per group. ^**^ *p* < 0.01; ^***^ *p* < 0.001.

Given that EDNRA is a druggable GPCR, we next investigated whether pharmacological inhibition of EDNRA could reproduce the suppressive effects observed after genetic depletion. MDA‐MB‐231 and BT549 cells were treated with the EDNRA antagonist atrasentan. Western blotting and RT‐qPCR analyses showed that EDNRA protein and mRNA levels decreased after atrasentan treatment in both TNBC cell lines (Figure 3A–D). Because atrasentan primarily functions as an EDNRA antagonist, this reduction was interpreted as a likely consequence of disrupting the EDNRA–YAP positive feedback loop rather than as direct transcriptional inhibition by atrasentan. Functionally, atrasentan markedly suppressed TNBC cell proliferation, as reflected by decreased CCK‐8 absorbance and fewer EdU‐positive proliferating cells (Figure 3E–J). Atrasentan also reduced the migratory capacity of MDA‐MB‐231 and BT549 cells, increased apoptotic cell populations, and decreased the CD44^high/CD24^low/− fraction (Figure 3K–V). These findings indicate that pharmacological blockade of EDNRA restrains TNBC cell proliferation, migration, and CD24/CD44‐defined stem‐like features while promoting apoptosis. We further validated the effect of atrasentan in HCC1806 cells. Consistent with the results in MDA‐MB‐231 and BT549 cells, atrasentan treatment was accompanied by reduced EDNRA protein expression and suppressed HCC1806 cell proliferation, as shown by CCK‐8 and EdU proliferation assays. Atrasentan also impaired HCC1806 cell migration and increased apoptosis (Figure S5A–H). Thus, the inhibitory effect of EDNRA blockade was consistently observed across multiple TNBC cell models. Finally, we evaluated the antitumor effect of atrasentan in vivo. Compared with vehicle treatment, atrasentan administration significantly reduced xenograft tumor size, tumor weight, and tumor volume (Figure 3W–Y). Histological analysis showed decreased EDNRA staining and reduced Ki‐67 positivity in tumors from the atrasentan‐treated group (Figure 3Z). Together, these data demonstrate that atrasentan‐mediated EDNRA blockade effectively restrains TNBC malignant progression in vitro and in vivo.

### 3.4 ET‐1 Stimulation Enhances EDNRA‐Dependent TNBC Cell Phenotypes

**FIGURE 4:**
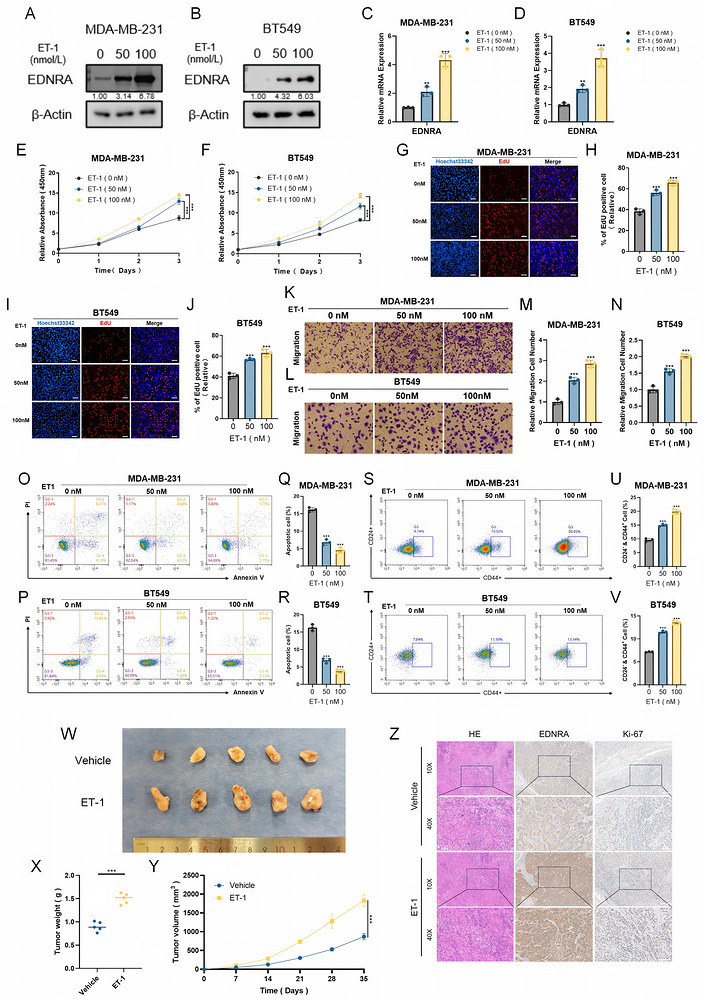
Endothelin‐1 (ET‐1) stimulation increases EDNRA expression and promotes malignant phenotypes of TNBC cells. (A, B) Western blot analysis of EDNRA protein expression in MDA‐MB‐231 and BT549 cells treated with ET‐1 at the indicated concentrations. β‐Actin was used as the loading control. (C, D) RT‐qPCR analysis of EDNRA mRNA expression in MDA‐MB‐231 and BT549 cells after ET‐1 stimulation. (E, F) CCK‐8 assays showing the proliferation of MDA‐MB‐231 and BT549 cells treated with ET‐1. (G–J) Representative EdU staining images and quantification of EdU‐positive MDA‐MB‐231 and BT549 cells following ET‐1 treatment (Hoechst 33342, blue; EdU, red). (K–N) Representative Transwell migration images and quantification of migrated MDA‐MB‐231 and BT549 cells after ET‐1 stimulation. (O–R) Flow cytometric analysis and quantification of apoptotic cells in MDA‐MB‐231 and BT549 cells treated with ET‐1. (S–V) Flow cytometric analysis and quantification of the CD44^high/CD24^low/− stem‐like population after ET‐1 treatment in MDA‐MB‐231 and BT549 cells. (W) Representative images of xenograft tumors from the vehicle and ET‐1‐treated groups. (X, Y) Quantification of xenograft tumor weight and tumor volume in the vehicle and ET‐1‐treated groups. (Z) Representative H&E, EDNRA, and Ki‐67 staining of xenograft tumor sections from the vehicle and ET‐1‐treated groups. Images are shown at 10× and 40× magnification. Data are presented as the mean ± SD. For xenograft experiments, n = 5 mice per group. ^**^ *p* < 0.01; ^***^ *p* < 0.001.

To complement the genetic depletion and antagonist‐based inhibition of EDNRA, we next examined whether activation of EDNRA signaling could promote TNBC progression. MDA‐MB‐231 and BT549 cells were treated with endothelin‐1 (ET‐1), the endogenous ligand of EDNRA. Western blotting and RT‐qPCR analyses showed that ET‐1 stimulation increased EDNRA protein and mRNA expression in a dose‐dependent manner in both TNBC cell lines (Figure 4A–D). Functionally, ET‐1 enhanced TNBC cell proliferation, as indicated by increased CCK‐8 absorbance and a higher proportion of EdU‐positive proliferating cells (Figure 4E–J). ET‐1 treatment also promoted Transwell migration, reduced apoptotic cell populations, and increased the CD44^high/CD24^low/− fraction in MDA‐MB‐231 and BT549 cells (Figure 4K–V). These results indicate that activation of the ET‐1/EDNRA axis is sufficient to enhance multiple malignant phenotypes, including CD24/CD44‐defined stem‐like features, in TNBC cells. We further validated the ligand‐stimulation effect in HCC1806 cells. Consistent with the findings above, ET‐1 increased EDNRA protein expression and promoted HCC1806 cell proliferation, as shown by CCK‐8 and EdU proliferation assays. ET‐1 also enhanced migratory ability and reduced apoptosis in HCC1806 cells (Figure S6A–H). These results provide additional support that ET‐1‐induced EDNRA activation promotes malignant TNBC phenotypes across distinct cellular models. Finally, we examined the effect of ET‐1 in vivo. Compared with the vehicle group, ET‐1 treatment significantly increased xenograft tumor size, tumor weight, and tumor volume (Figure 4W–Y). Histological analysis further showed stronger EDNRA staining and increased Ki‐67 positivity in ET‐1‐treated tumors (Figure 4Z). Together with the EDNRA knockdown and atrasentan experiments, these data demonstrate that activation of the ET‐1/EDNRA signaling axis facilitates TNBC malignant progression in vitro and in vivo.

### 3.5 EDNRA Gain of Function Reinforces Aggressive TNBC Cell Behavior

To further determine whether EDNRA itself is sufficient to enhance malignant phenotypes, we performed gain‐of‐function experiments by transfecting Flag‐EDNRA into MDA‐MB‐231 and HCC1806 cells. Western blotting confirmed dose‐dependent Flag‐EDNRA overexpression in both cell lines (Figure S7A,B). Functionally, EDNRA overexpression increased cell proliferation, as shown by CCK‐8 assays and the increased proportion of EdU‐positive proliferating cells (Figure S7C,D,M–P). In parallel, Flag‐EDNRA overexpression enhanced Transwell migration and reduced apoptotic cell populations in both MDA‐MB‐231 and HCC1806 cells (Figure S7E–L). These gain‐of‐function findings complement the EDNRA knockdown, atrasentan blockade, and ET‐1 stimulation results, further supporting that EDNRA activation is sufficient to promote malignant TNBC phenotypes across multiple EDNRA‐expressing TNBC cell models.

### 3.6 EDNRA Activates Hippo/YAP Signaling in TNBC Cells

**FIGURE 5:**
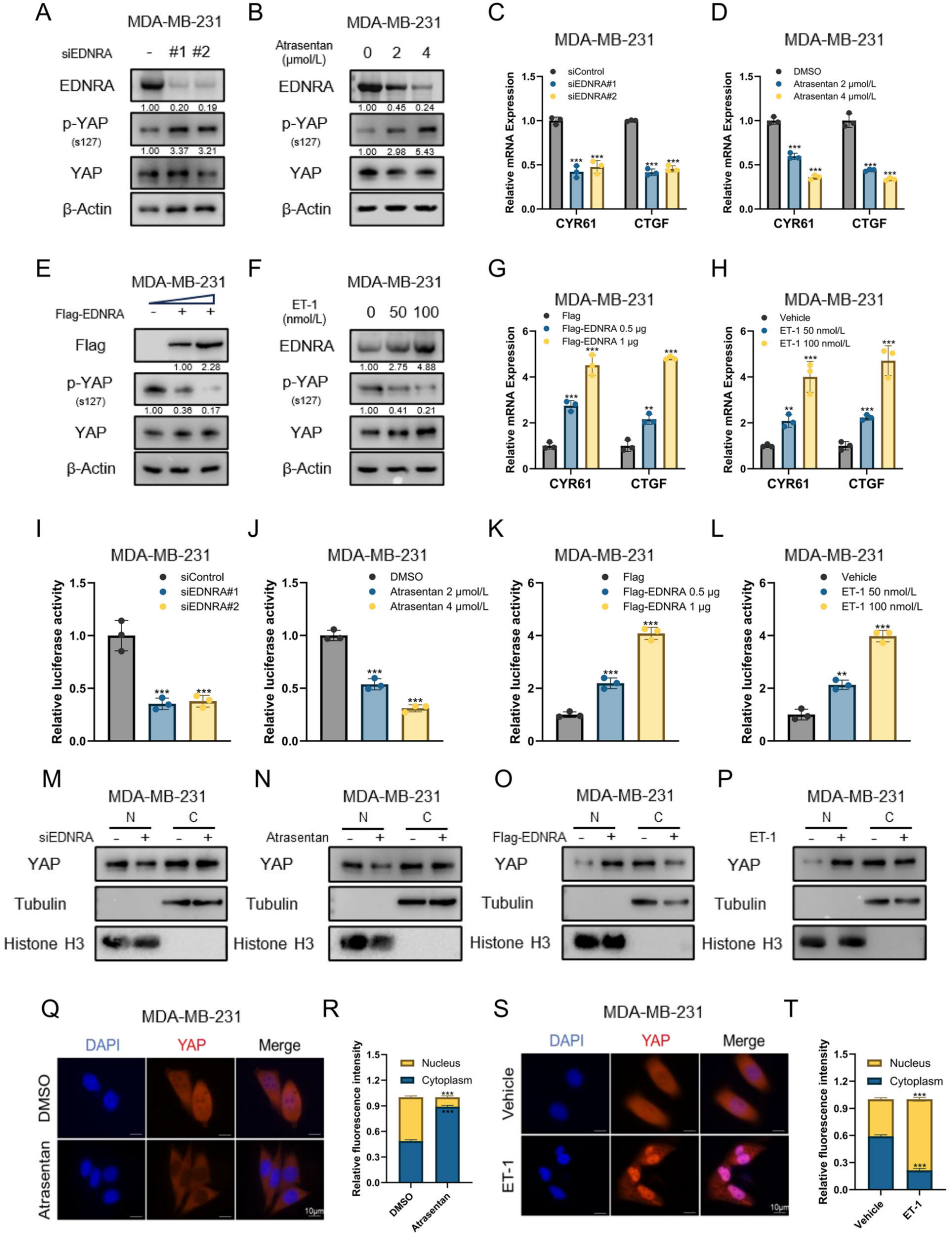
EDNRA regulates YAP phosphorylation, nuclear localization, and TEAD transcriptional activity. (A) Western blot analysis of EDNRA, phosphorylated YAP (p‐YAP) Ser127, and total YAP expression in MDA‐MB‐231 cells transfected with siControl or siEDNRA. β‐Actin was used as the loading control. (B) Western blot analysis of EDNRA, p‐YAP Ser127 and total YAP in MDA‐MB‐231 cells treated with atrasentan at the indicated concentrations. (C, D) RT‐qPCR analysis of CYR61 and CTGF mRNA expression after EDNRA knockdown or atrasentan treatment in MDA‐MB‐231 cells. (E) Western blot analysis of Flag, p‐YAP Ser127, and total YAP in MDA‐MB‐231 cells transfected with increasing amounts of Flag‐EDNRA plasmid. (F) Western blot analysis of EDNRA, p‐YAP Ser127 and total YAP in MDA‐MB‐231 cells treated with ET‐1 at the indicated concentrations. (G, H) RT‐qPCR analysis of CYR61 and CTGF mRNA expression after EDNRA overexpression or ET‐1 stimulation. (I–L) TEAD luciferase reporter assays in MDA‐MB‐231 cells after EDNRA knockdown, atrasentan treatment, EDNRA overexpression or ET‐1 stimulation. (M–P) Nuclear and cytoplasmic fractionation analysis of YAP localization after EDNRA knockdown, atrasentan treatment, EDNRA overexpression, or ET‐1 stimulation. Tubulin and Histone H3 were used as cytoplasmic and nuclear markers, respectively. (Q, R) Representative immunofluorescence staining and quantification of YAP localization in MDA‐MB‐231 cells treated with dimethyl sulfoxide (DMSO) or atrasentan. (S, T) Representative immunofluorescence staining and quantification of YAP localization in MDA‐MB‐231 cells treated with vehicle or ET‐1. Scale bars, 10 µm. Data are presented as the mean ± SD. ns, not significant; ^**^ *p* < 0.01; ^***^ *p* < 0.001.

After confirming the pro‐tumorigenic function of EDNRA, we next investigated whether EDNRA regulates the Hippo/YAP pathway in TNBC cells. In MDA‐MB‐231 cells, siRNA‐mediated EDNRA depletion increased YAP phosphorylation at Ser127, whereas total YAP expression was not markedly changed (Figure 5A). Similar results were observed after treatment with the EDNRA antagonist atrasentan, which increased p‐YAP levels in a dose‐dependent manner (Figure 5B). Consistently, EDNRA knockdown or atrasentan treatment significantly reduced the expression of the classical YAP target genes CYR61 and CTGF (Figure 5C,D). Conversely, Flag‐EDNRA overexpression or ET‐1 stimulation decreased YAP Ser127 phosphorylation and increased CYR61 and CTGF expression (Figure 5E–H). TEAD luciferase reporter assays further showed that EDNRA depletion or atrasentan treatment impaired TEAD transcriptional activity, whereas EDNRA overexpression or ET‐1 stimulation enhanced TEAD reporter activity (Figure 5I–L). Because YAP phosphorylation at Ser127 regulates its subcellular localization, we next examined YAP distribution. Nuclear and cytoplasmic fractionation showed that EDNRA knockdown and atrasentan treatment reduced nuclear YAP accumulation, whereas EDNRA overexpression and ET‐1 stimulation promoted YAP nuclear localization (Figure 5M–P). Immunofluorescence staining further confirmed that atrasentan decreased nuclear YAP, while ET‐1 increased YAP nuclear accumulation (Figure 5Q–T). These findings indicate that EDNRA activates YAP signaling by promoting YAP dephosphorylation, nuclear localization, and TEAD‐dependent transcription.

To further determine whether this regulatory pattern was reproducible in an additional TNBC model, we performed parallel analyses in HCC1806 cells. Consistent with the results in MDA‐MB‐231 cells, EDNRA depletion or atrasentan treatment increased YAP Ser127 phosphorylation and reduced CYR61 and CTGF expression, whereas EDNRA overexpression or ET‐1 stimulation produced the opposite effects (Figure S8A–H). TEAD luciferase reporter assays similarly showed that EDNRA inhibition suppressed, while EDNRA activation enhanced, TEAD transcriptional activity in HCC1806 cells (Figure S8I–L). Immunofluorescence analysis further confirmed that atrasentan reduced YAP nuclear localization, whereas ET‐1 promoted YAP nuclear accumulation in HCC1806 cells (Figure S8M–P). These results support that EDNRA‐mediated YAP activation is consistently observed across distinct TNBC cell models. Given the functional relationship between YAP and TAZ and the potential overlapping roles of LATS1 and LATS2 within the Hippo kinase cascade, we further examined the LATS2/TAZ branch as a compensatory pathway. Under EDNRA knockdown or Flag‐EDNRA overexpression, total LATS2 and total TAZ levels were not substantially altered in either MDA‐MB‐231 or HCC1806 cells. In contrast to the clear regulation of YAP Ser127 phosphorylation observed above, phosphorylated TAZ at Ser89 did not show a consistent or substantial reciprocal change following EDNRA depletion or overexpression (Figure S8Q–T). These data suggest that EDNRA‐mediated Hippo pathway output in the present TNBC models is mainly supported by changes in YAP phosphorylation, YAP subcellular localization, and TEAD‐dependent transcription, without an obvious compensatory alteration in the LATS2/TAZ branch. Collectively, these findings demonstrate that EDNRA activates a YAP‐centered Hippo signaling program in TNBC cells.

### 3.7 YAP Functionally Mediates EDNRA‐Driven Malignant Phenotypes in TNBC

Having established that EDNRA regulates YAP phosphorylation, nuclear localization, and TEAD‐dependent transcription, we next investigated whether YAP functionally mediates the malignant phenotypes driven by EDNRA. In rescue experiments, Myc‐YAP was re‐expressed in EDNRA‐depleted MDA‐MB‐231 and HCC1806 cells. Western blotting and RT‐qPCR confirmed EDNRA knockdown and Myc‐YAP expression in both cell lines (Figure S9A–D). Functionally, YAP re‐expression restored CYR61 and CTGF expression and rescued TEAD reporter activity suppressed by EDNRA depletion (Figure S9E–H). Consistently, Myc‐YAP largely reversed the inhibitory effects of EDNRA knockdown on cell proliferation, Transwell migration, and EdU‐positive proliferating cells, while reducing the apoptosis induced by EDNRA depletion in both MDA‐MB‐231 and HCC1806 cells (Figure S9I–V). In vivo, YAP re‐expression also restored xenograft tumor growth suppressed by shEDNRA, as reflected by increased tumor size, tumor weight, and tumor volume (Figure S9W–Y). To further determine whether YAP is required for EDNRA gain‐of‐function effects, we performed the reciprocal experiment by knocking down YAP in Flag‐EDNRA‐overexpressing MDA‐MB‐231 cells. YAP depletion attenuated EDNRA overexpression‐induced activation of CYR61 and CTGF expression and markedly reduced TEAD reporter activity (Figure S10A–D). Moreover, YAP knockdown weakened the proliferative and migratory advantages conferred by EDNRA overexpression and restored apoptotic cell populations (Figure S10E–I). These complementary rescue and blockade experiments demonstrate that YAP is a critical downstream effector required for EDNRA‐driven malignant phenotypes in TNBC cells.

### 3.8 EDNRA Activates YAP Through the Gαq/11–Rho/ROCK–LATS Cascade

**FIGURE 6:**
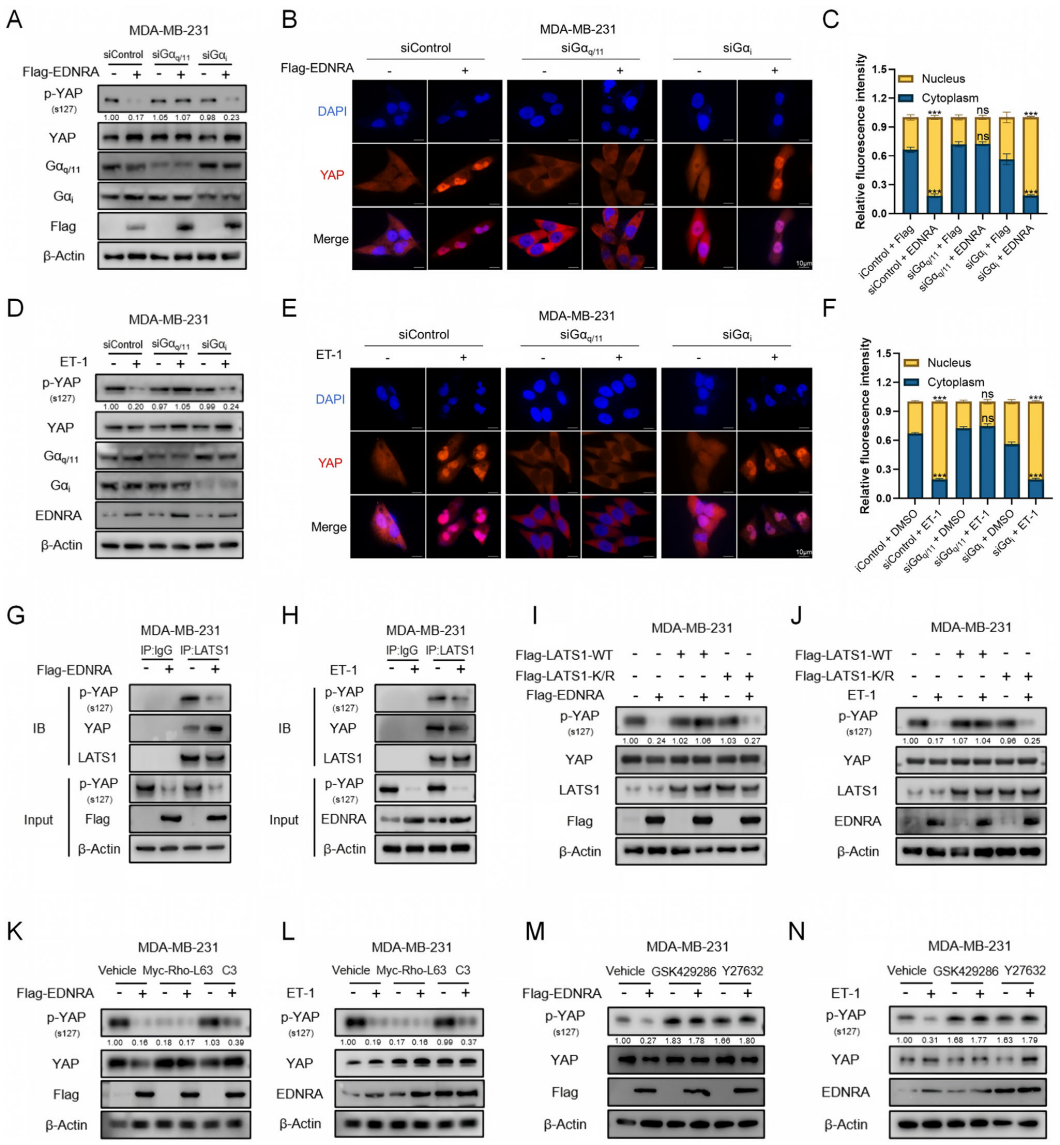
Gαq/11, Rho/ROCK (Rho‐associated coiled‐coil‐containing protein kinase) and LATS1 mediate EDNRA‐induced YAP dephosphorylation. (A) Western blot analysis of p‐YAP Ser127 and total YAP in MDA‐MB‐231 cells transfected with Flag‐EDNRA together with siControl, siGαq/11 or siGαi. β‐Actin was used as the loading control. (B, C) Representative immunofluorescence staining and quantification of YAP localization in MDA‐MB‐231 cells transfected with Flag‐EDNRA under siControl, siGαq/11 or siGαi conditions. Scale bars, 10 µm. (D) Western blot analysis of p‐YAP Ser127 and total YAP in MDA‐MB‐231 cells treated with ET‐1 after siRNA‐mediated depletion of Gαq/11 or Gαi. (E, F) Representative immunofluorescence staining and quantification of YAP localization in MDA‐MB‐231 cells treated with ET‐1 under siControl, siGαq/11 or siGαi conditions. Scale bars, 10 µm. (G, H) Immunoprecipitation analysis of LATS1‐associated YAP and p‐YAP in MDA‐MB‐231 cells after Flag‐EDNRA overexpression or ET‐1 stimulation. (I, J) Western blot analysis of p‐YAP Ser127 and total YAP in MDA‐MB‐231 cells transfected with wild‐type LATS1 or kinase‐defective LATS1‐K/R, with or without Flag‐EDNRA overexpression or ET‐1 stimulation. (K, L) Western blot analysis of p‐YAP Ser127 and total YAP in MDA‐MB‐231 cells treated with Myc‐Rho‐L63 or C3 in the presence or absence of Flag‐EDNRA overexpression or ET‐1 stimulation. (M, N) Western blot analysis of p‐YAP Ser127 and total YAP in MDA‐MB‐231 cells treated with ROCK inhibitors GSK429286 or Y27632, with or without Flag‐EDNRA overexpression or ET‐1 stimulation. Data are presented as the mean ± SD. ns, not significant; ^***^ *p* < 0.001.

GPCRs can transmit extracellular signals through distinct G‐protein subunits. We therefore investigated which G‐protein branch is required for EDNRA‐mediated YAP activation. In MDA‐MB‐231 cells, Flag‐EDNRA overexpression reduced YAP phosphorylation at Ser127. However, depletion of Gαq/11 largely abolished the effect of EDNRA on YAP dephosphorylation, whereas knockdown of Gαi did not produce a comparable effect (Figure 6A). Immunofluorescence staining further showed that EDNRA overexpression promoted YAP nuclear accumulation in control and siGαi‐treated cells, but this effect was impaired after Gαq/11 depletion (Figure 6B,C). These findings suggest that EDNRA activates YAP mainly through Gαq/11 rather than Gαi signaling. We next examined whether ligand‐induced EDNRA activation follows the same mechanism. ET‐1 stimulation reduced p‐YAP levels and promoted YAP nuclear localization in control cells. In contrast, Gαq/11 knockdown markedly blocked ET‐1‐induced YAP dephosphorylation and nuclear accumulation, while Gαi knockdown showed a weaker effect (Figure 6D–F). Thus, both EDNRA overexpression and ET‐1 stimulation require Gαq/11 to activate YAP. Because LATS1 directly phosphorylates YAP at Ser127, we further explored whether EDNRA regulates YAP through LATS1. Immunoprecipitation assays showed that EDNRA overexpression or ET‐1 stimulation reduced LATS1‐associated p‐YAP signals, indicating impaired LATS1‐mediated YAP phosphorylation (Figure 6G,H). Moreover, overexpression of wild‐type LATS1 restored YAP phosphorylation and attenuated the effects of EDNRA or ET‐1, whereas kinase‐defective LATS1‐K/R failed to produce a similar effect (Figure 6I,J). These results support that LATS1 kinase activity is involved in EDNRA‐mediated regulation of YAP. We then examined the downstream signaling events linking Gαq/11 to LATS1. Activation of Rho signaling by Myc‐Rho‐L63 reduced YAP phosphorylation, while inhibition of Rho by C3 increased p‐YAP and blocked the effect of EDNRA overexpression or ET‐1 stimulation (Figure 6K,L). Similarly, treatment with the ROCK inhibitors GSK429286 and Y27632 increased YAP phosphorylation and weakened EDNRA‐ or ET‐1‐induced YAP dephosphorylation (Figure 6M,N). Taken together, these data demonstrate that EDNRA activates YAP through a Gαq/11–Rho/ROCK–LATS signaling cascade in TNBC cells.

### 3.9 Gαq/11‐Binding Residues Are Required for EDNRA‐mediated YAP Activation

**FIGURE 7:**
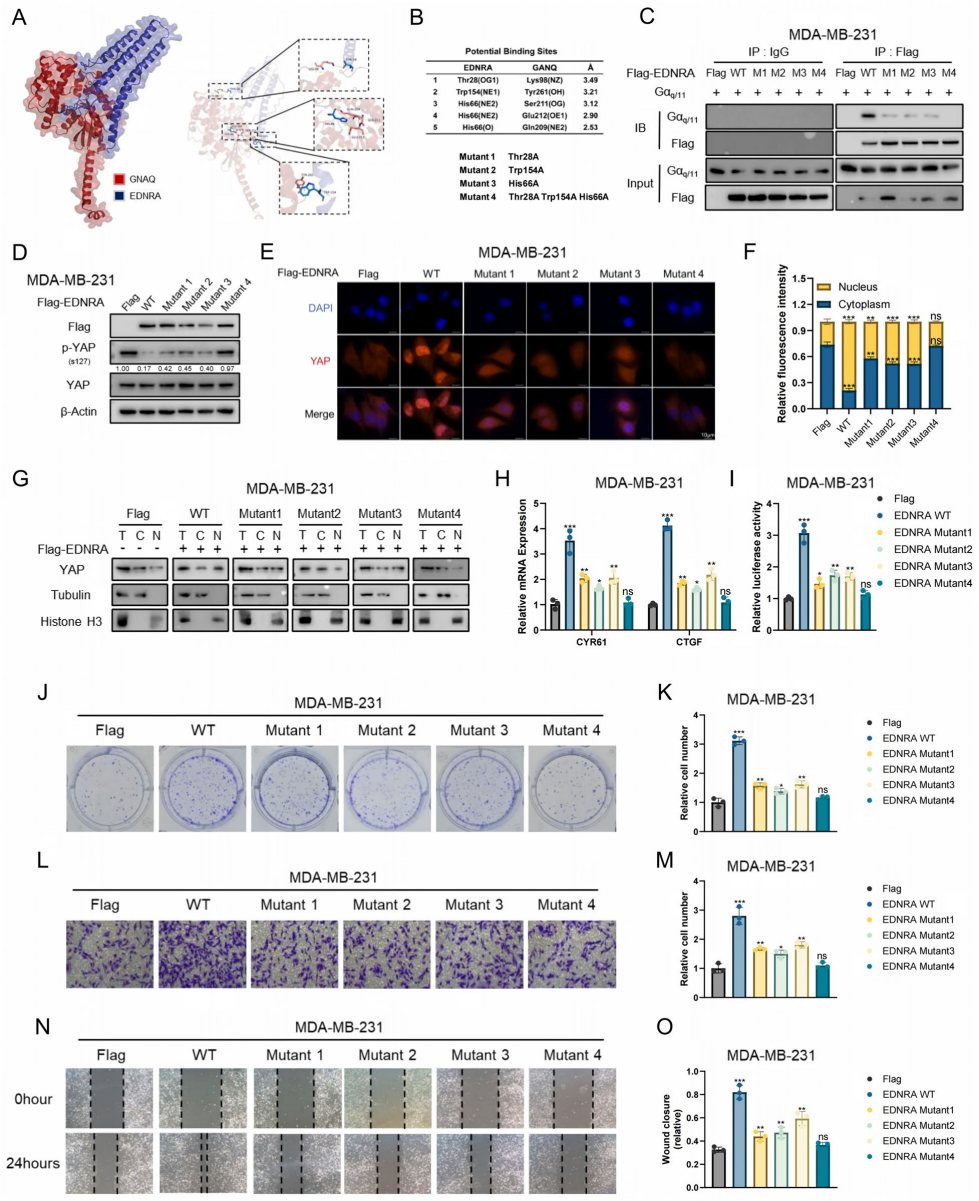
EDNRA mutants defective in Gαq/11 binding show impaired YAP activation and tumor‐promoting activity. (A) Molecular docking analysis showing the predicted interaction model between EDNRA and GNAQ. EDNRA is shown in blue, and GNAQ is shown in red. Enlarged views indicate the predicted binding interfaces. (B) Predicted EDNRA–GNAQ contact residues identified by molecular docking analysis and the corresponding EDNRA mutants used in this study. Mutant 1, Thr28A; Mutant 2, Trp154A; Mutant 3, His66A; Mutant 4, Thr28A/Trp154A/His66A. (C) Co‐immunoprecipitation analysis of the interaction between Gαq/11 and wild‐type or mutant Flag‐EDNRA in MDA‐MB‐231 cells. (D) Western blot analysis of p‐YAP Ser127 and total YAP in MDA‐MB‐231 cells expressing wild‐type or mutant EDNRA. β‐Actin was used as the loading control. (E, F) Representative immunofluorescence staining and quantification of YAP localization in MDA‐MB‐231 cells expressing wild‐type or mutant EDNRA. Scale bars, 10 µm. (G) Nuclear and cytoplasmic fractionation analysis of YAP localization in MDA‐MB‐231 cells expressing wild‐type or mutant EDNRA. Tubulin and Histone H3 were used as cytoplasmic and nuclear markers, respectively. (H) RT‐qPCR analysis of CYR61 and CTGF mRNA expression in MDA‐MB‐231 cells expressing wild‐type or mutant EDNRA. (I) TEAD luciferase reporter assay in MDA‐MB‐231 cells expressing wild‐type or mutant EDNRA. (J, K) Representative colony formation images and quantification in MDA‐MB‐231 cells expressing wild‐type or mutant EDNRA. (L, M) Representative Transwell migration images and quantification of migrated MDA‐MB‐231 cells expressing wild‐type or mutant EDNRA. (N, O) Representative wound‐healing images and quantification of wound closure in MDA‐MB‐231 cells expressing wild‐type or mutant EDNRA. Data are presented as the mean ± SD. ns, not significant; ^**^ *p* < 0.01; ^***^ *p* < 0.001.

After demonstrating that EDNRA activates YAP mainly through Gαq/11 signaling, we next sought to define whether the interaction between EDNRA and Gαq/11 is required for this regulatory process. Molecular docking analysis of EDNRA with GNAQ predicted several potential interaction interfaces between the receptor and Gαq. Among the predicted contact sites, Thr28, Trp154, and His66 of EDNRA were located at the putative EDNRA–GNAQ binding interface (Figure 7A,B). On the basis of these docking results, we generated EDNRA mutants carrying Thr28A, Trp154A, His66A, or a combined triple mutation to disrupt the predicted binding sites. Co‐immunoprecipitation assays showed that wild‐type EDNRA interacted with Gαq/11, whereas the EDNRA mutants exhibited weakened binding ability. Notably, the combined mutant showed the most evident reduction in Gαq/11 association (Figure 7C). We then examined whether these mutations affected EDNRA‐mediated YAP activation. Compared with wild‐type EDNRA, single EDNRA mutants showed a reduced ability to decrease YAP phosphorylation at Ser127, while the combined mutant largely failed to suppress YAP phosphorylation (Figure 7D). Consistently, immunofluorescence staining showed that wild‐type EDNRA markedly promoted YAP nuclear accumulation, whereas the EDNRA mutants, especially the combined mutant, showed impaired capacity to induce YAP nuclear localization (Figure 7E,F). Nuclear and cytoplasmic fractionation further confirmed that disruption of the predicted Gαq/11‐binding residues weakened EDNRA‐induced YAP nuclear translocation (Figure 7G). We further evaluated the transcriptional activity of YAP. Wild‐type EDNRA strongly increased the expression of CYR61 and CTGF and enhanced TEAD luciferase reporter activity. In contrast, EDNRA mutants displayed reduced effects on YAP target gene expression and TEAD activity, with the combined mutant showing minimal activity (Figure 7H,I). These data indicate that the predicted Gαq/11‐interacting residues are necessary for EDNRA‐dependent activation of the YAP transcriptional program. Finally, we investigated whether these residues are required for EDNRA‐associated malignant phenotypes. Wild‐type EDNRA increased colony formation, Transwell migration and wound closure in MDA‐MB‐231 cells. However, these effects were attenuated by the single mutations and were almost abolished by the combined mutation (Figure 7J–O). Taken together, these findings suggest that the predicted Gαq/11‐interacting residues are important for EDNRA‐mediated YAP activation and for the malignant phenotypes associated with EDNRA activation in TNBC cells.

### 3.10 YAP/TEAD4 Reinforces EDNRA Transcription Through an Enhancer‐Dependent Mechanism

**FIGURE 8:**
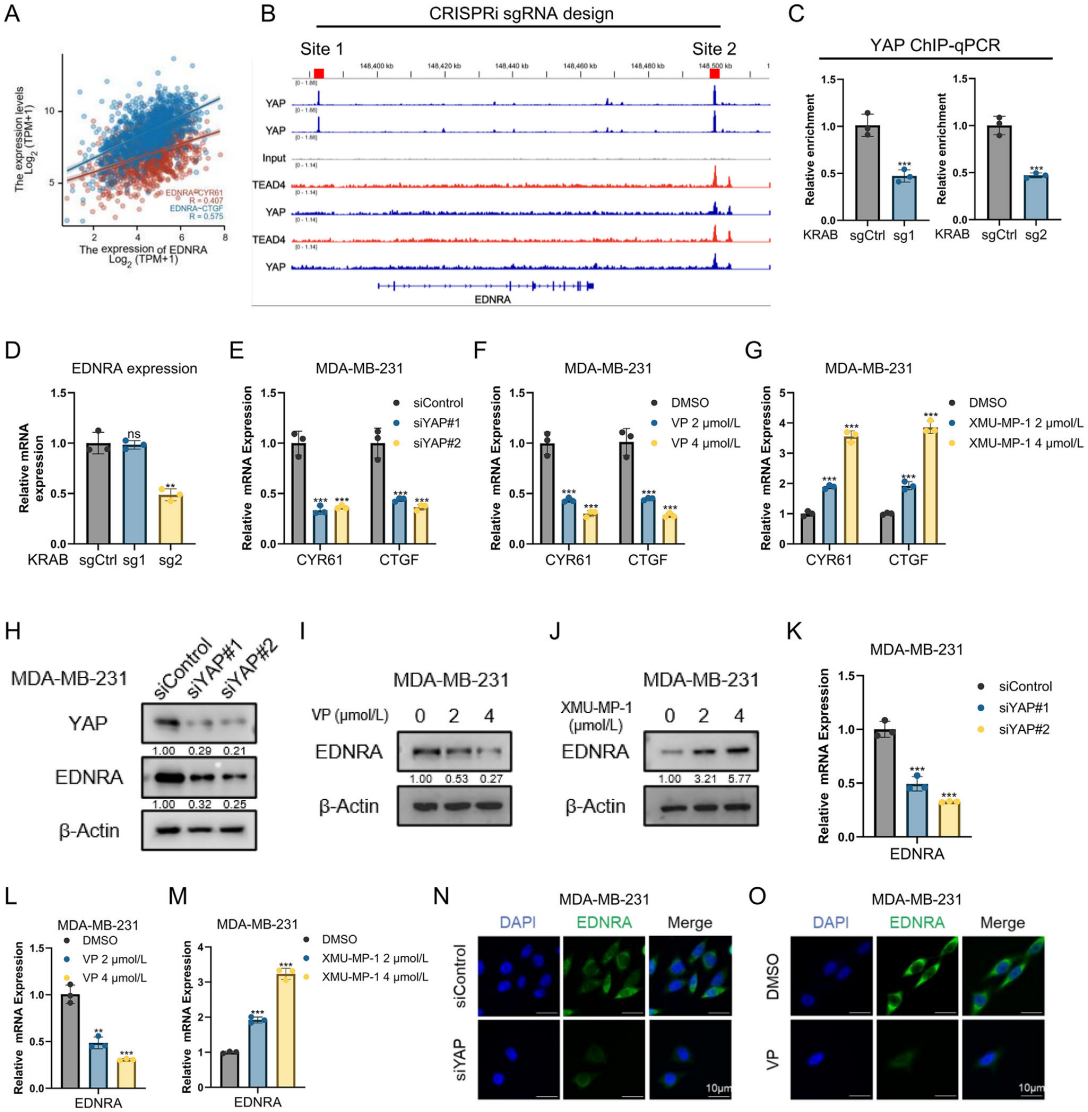
YAP/TEAD4 promotes EDNRA expression through an enhancer‐associated regulatory region. (A) Correlation analysis between EDNRA expression and the expression of YAP target genes CYR61 and CTGF in breast cancer samples. Correlation coefficients are indicated in the plot. (B) Chromatin immunoprecipitation sequencing (ChIP‐seq) tracks showing YAP and TEAD4 occupancy around the EDNRA genomic locus. Candidate promoter‐ and enhancer‐associated regulatory regions were selected for CRISPR interference (CRISPRi) single‐guide RNA (sgRNA) design. (C) YAP chromatin immunoprecipitation quantitative PCR (ChIP‐qPCR) analysis of the indicated EDNRA regulatory regions after CRISPRi‐mediated targeting. (D) RT‐qPCR analysis of EDNRA mRNA expression in MDA‐MB‐231 cells expressing KRAB with sgControl or sgRNAs targeting the selected regulatory regions. Repression of the enhancer‐associated region significantly reduced EDNRA expression. (E) RT‐qPCR analysis of CYR61 and CTGF mRNA expression after YAP depletion in MDA‐MB‐231 cells. (F) RT‐qPCR analysis of CYR61 and CTGF mRNA expression in MDA‐MB‐231 cells treated with verteporfin at the indicated concentrations. (G) RT‐qPCR analysis of CYR61 and CTGF mRNA expression in MDA‐MB‐231 cells treated with XMU‐MP‐1 at the indicated concentrations. (H) Western blot analysis of YAP and EDNRA expression after YAP knockdown in MDA‐MB‐231 cells. β‐Actin was used as the loading control. (I) Western blot analysis of EDNRA expression after verteporfin treatment in MDA‐MB‐231 cells. (J) Western blot analysis of EDNRA expression after XMU‐MP‐1 treatment in MDA‐MB‐231 cells. (K) RT‐qPCR analysis of EDNRA mRNA expression after YAP knockdown. (L) RT‐qPCR analysis of EDNRA mRNA expression after verteporfin treatment. (M) RT‐qPCR analysis of EDNRA mRNA expression after XMU‐MP‐1 treatment. (N) Representative immunofluorescence staining of EDNRA in MDA‐MB‐231 cells after YAP knockdown. Scale bars, 10 µm. (O) Representative immunofluorescence staining of EDNRA in MDA‐MB‐231 cells after verteporfin treatment. Scale bars, 10 µm. Data are presented as the mean ± SD. ns, not significant; ^**^ *p* < 0.01; ^***^ *p* < 0.001.

Having demonstrated that EDNRA activates YAP signaling through the Gαq/11–Rho/ROCK–LATS cascade, we next investigated whether YAP/TEAD4 could in turn regulate EDNRA expression. In TCGA‐BRCA samples, EDNRA expression was positively correlated with the expression of classical YAP target genes, including CYR61 and CTGF, supporting a close transcriptional association between EDNRA and YAP activity (Figure 8A). To determine whether YAP/TEAD4 directly participates in EDNRA transcriptional regulation, we analyzed YAP and TEAD4 chromatin occupancy around the EDNRA locus. ChIP‐seq tracks showed YAP and TEAD4 enrichment at regulatory regions near EDNRA, including candidate promoter‐ and enhancer‐associated sites. We therefore designed CRISPRi sgRNAs targeting these regions to determine which regulatory element was functionally required for EDNRA transcription (Figure 8B). YAP ChIP‐qPCR confirmed YAP enrichment at the selected regions (Figure 8C). Notably, CRISPRi‐mediated repression of the candidate enhancer‐associated region significantly reduced EDNRA mRNA expression, whereas repression of the other tested site showed no obvious effect (Figure 8D). These data suggest that YAP/TEAD4 promotes EDNRA expression, at least partly, through this enhancer‐associated regulatory region. We further examined whether changing YAP activity could modulate EDNRA expression. YAP knockdown significantly reduced the expression of CYR61 and CTGF, confirming suppression of YAP transcriptional activity (Figure 8E). Similarly, verteporfin treatment decreased CYR61 and CTGF expression, whereas XMU‐MP‐1 treatment increased their expression (Figure 8F,G). Under the same conditions, EDNRA expression changed consistently with YAP activity. YAP depletion reduced EDNRA protein and mRNA levels, and verteporfin also decreased EDNRA expression (Figure 8H,I,K,L). In contrast, XMU‐MP‐1 increased EDNRA protein and mRNA expression (Figure 8J,M). Immunofluorescence staining further confirmed that EDNRA protein abundance was reduced after YAP knockdown or verteporfin treatment in MDA‐MB‐231 cells (Figure 8N,O). Taken together, these results indicate that EDNRA functions not only as an upstream activator of Hippo/YAP signaling but also as a downstream transcriptional target of YAP/TEAD4. Thus, EDNRA activates YAP, while YAP/TEAD4 in turn reinforces EDNRA expression through a candidate enhancer‐associated regulatory region, supporting a positive feedback loop in TNBC cells.

### 3.11 Atrasentan Sensitizes TNBC Cells to Paclitaxel Treatment

**FIGURE 9:**
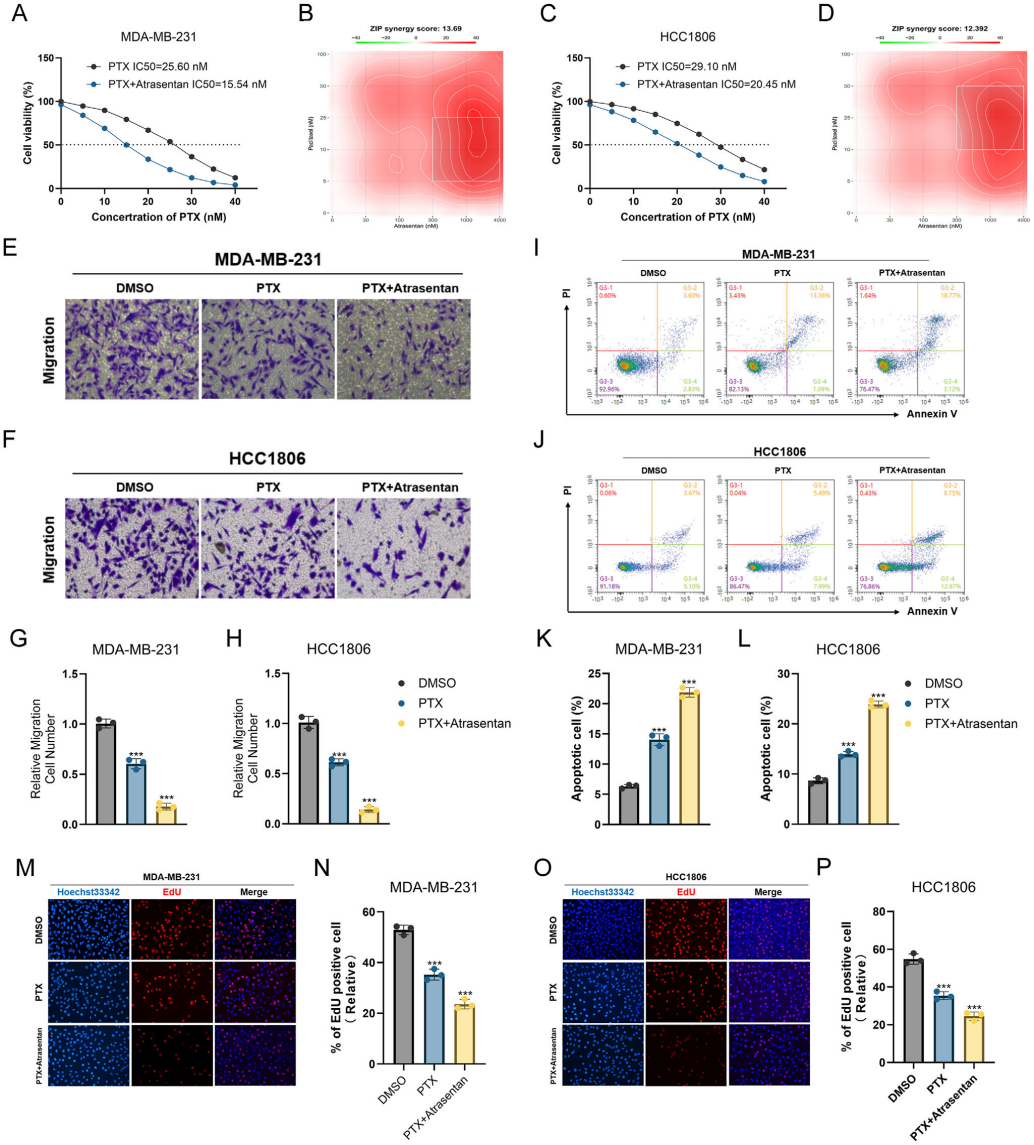
Atrasentan sensitizes TNBC cells to paclitaxel and enhances paclitaxel‐induced anti‐tumor cellular effects. (A) Cell viability dose–response curve of MDA‐MB‐231 cells treated with increasing concentrations of paclitaxel (PTX) alone or in combination with a fixed low dose of atrasentan at 300 nM. The calculated IC50 values are shown. (B) Zero interaction potency (ZIP) synergy heatmap showing the interaction between PTX and atrasentan in MDA‐MB‐231 cells. The overall ZIP synergy score is shown. (C) Cell viability dose–response curve of HCC1806 cells treated with increasing concentrations of PTX alone or in combination with 300 nM atrasentan. The calculated IC50 values are shown. (D) ZIP synergy heatmap showing the interaction between PTX and atrasentan in HCC1806 cells. The overall ZIP synergy score is shown. (E–H) Representative Transwell migration images and quantification of migrated MDA‐MB‐231 and HCC1806 cells after treatment with DMSO, PTX, or PTX plus 300 nM atrasentan. (I–L) Representative Annexin V/propidium iodide flow cytometry plots and quantification of apoptotic MDA‐MB‐231 and HCC1806 cells after treatment with DMSO, PTX, or PTX plus 300 nM atrasentan. (M–P) Representative EdU staining images and quantification of EdU‐positive proliferating cells in MDA‐MB‐231 and HCC1806 cells after treatment with DMSO, PTX, or PTX plus 300 nM atrasentan (Hoechst 33342, blue; EdU, red). Data are presented as the mean ± SD. ns, not significant; ^**^ *p* < 0.01; ^***^ *p* < 0.001.

Given the inhibitory effect of EDNRA blockade in TNBC cells, we next examined whether atrasentan could enhance the therapeutic response to paclitaxel (PTX), a commonly used chemotherapeutic agent for TNBC. A fixed low dose of atrasentan at 300 nM was selected for combination analysis because this concentration exerted only a limited inhibitory effect as a single agent in the dose–response matrix, allowing evaluation of its chemosensitizing activity. In MDA‐MB‐231 cells, 300 nM atrasentan reduced the IC50 of PTX from 25.60 to 15.54 nM (Figure 9A). Similarly, in HCC1806 cells, atrasentan reduced the IC50 of PTX from 29.10 to 20.45 nM (Figure 9C). Dose–response matrix analysis further showed that the combination of PTX and atrasentan produced positive synergistic interactions in both cell lines, with zero interaction potency (ZIP) synergy scores of 13.69 in MDA‐MB‐231 cells and 12.392 in HCC1806 cells (Figure 9B,D and Figure S11A–D). To further validate the drug interaction using an independent pharmacological model, we performed Chou–Talalay combination index (CI) analysis. The CI values at ED50 were 0.503 in MDA‐MB‐231 cells and 0.497 in HCC1806 cells, both below 1, further supporting a synergistic interaction between PTX and atrasentan (Figure S11E, F) [24, 25]. We next evaluated whether this combination enhanced PTX‐induced cellular effects beyond cell viability inhibition. Compared with PTX alone, PTX plus 300 nM atrasentan further suppressed Transwell migration in both MDA‐MB‐231 and HCC1806 cells (Figure 9E–H). Flow cytometric analysis showed that the combination treatment induced a higher proportion of apoptotic cells than PTX alone (Figure 9I–L). In addition, EdU proliferation assays showed that PTX plus atrasentan further reduced the proportion of EdU‐positive proliferating cells compared with PTX treatment alone (Figure 9M–P). Together, these findings indicate that low‐dose atrasentan sensitizes TNBC cells to PTX and enhances PTX‐induced suppression of malignant cellular phenotypes.

## 4 Discussion

In the present study, we identified EDNRA as a druggable GPCR associated with sustained Hippo/YAP activation and aggressive TNBC phenotypes. Integrated GPCR correlation analysis, marketed‐drug‐targeted GPCR annotation, and siRNA screening using CTGF as a YAP readout nominated EDNRA as a candidate upstream regulator of Hippo/YAP signaling. EDNRA was elevated in breast cancer and TNBC tissues, associated with adverse clinicopathological features, and positively correlated with YAP target signatures and YAP protein expression. Functionally, EDNRA depletion or atrasentan‐mediated blockade suppressed proliferation, migration, stem‐like features, survival, and xenograft tumor growth, whereas EDNRA overexpression or ET‐1 stimulation produced the opposite effects. Mechanistically, EDNRA activated Hippo pathway output through the Gαq/11–Rho/ROCK–LATS cascade, leading to reduced YAP Ser127 phosphorylation, YAP nuclear accumulation, and enhanced TEAD‐dependent transcription, whereas the LATS2/TAZ branch did not show an obvious compensatory alteration under these conditions. Importantly, YAP/TEAD4 in turn reinforced EDNRA transcription through an enhancer‐associated regulatory region. These findings define an EDNRA–YAP positive feedback loop that links extracellular endothelin signaling to persistent YAP transcriptional output in TNBC.

This loop should be considered within the current therapeutic landscape of TNBC. Current strategies, including chemotherapy, immune checkpoint blockade, PARP inhibition, and antibody–drug conjugates, have improved outcomes in selected patient subsets but have not eliminated recurrence or metastatic progression [1, 7]. YAP/TAZ signaling is relevant in this context because it supports metastatic plasticity, cancer stem‐like states, immune remodeling, and resistance to multiple anticancer therapies [26, 27]. A central unresolved question is why YAP remains active in tumors without recurrent genetic loss of MST/LATS kinases. The EDNRA–YAP loop provides one explanation: TNBC cells can use an extracellular ligand‐responsive receptor to maintain YAP activity and then use YAP/TEAD4 to increase expression of the same receptor. This architecture converts a membrane signal into a self‐reinforcing transcriptional state.

The GPCR–Hippo connection offers a mechanistic framework for this finding. GPCRs can regulate YAP/TAZ through G‐protein branches, Rho GTPases, actin remodeling, and LATS kinase activity [12, 13]. EDNRA fits this framework but adds TNBC‐specific detail. Our data show that EDNRA relies mainly on Gαq/11 rather than Gαi to reduce YAP phosphorylation, and that Rho or ROCK inhibition restores YAP phosphorylation. Molecular docking and mutational analysis further show that disrupting predicted EDNRA–Gαq/11 contact residues weakens YAP activation and malignant phenotypes. Thus, EDNRA is not only correlated with Hippo/YAP activity but also provides a defined membrane‐to‐nucleus signaling route. This distinguishes EDNRA from general pathway associations and supports its functional role as an upstream YAP activator in TNBC.

Although our mechanistic analyses focused primarily on the Gαq/11–Rho/ROCK–LATS–YAP signaling cascade, the Hippo pathway functions through closely related YAP and TAZ effectors, and LATS1 and LATS2 may exert overlapping or compensatory roles. Therefore, we additionally examined the LATS2/TAZ branch. In contrast to the robust regulation of YAP Ser127 phosphorylation, YAP nuclear localization, and TEAD transcriptional activity, EDNRA depletion or overexpression did not cause a consistent or substantial change in total LATS2, total TAZ, or TAZ Ser89 phosphorylation in the examined TNBC models. These findings suggest that the EDNRA‐dependent Hippo pathway output observed in this study is predominantly YAP‐centered, rather than being accompanied by an overt compensatory alteration of the LATS2/TAZ branch. This result also narrows the mechanistic interpretation of EDNRA signaling and supports focusing the proposed positive feedback loop on EDNRA and YAP/TEAD4.

The endothelin pathway also links tumor cells and their microenvironment. ET‐1 can be generated by tumor, endothelial, fibroblast, and stromal compartments, allowing endothelin receptors to integrate vascular, hypoxic, and matrix‐associated signals [16, 17]. In colorectal cancer, endothelin activates YAP/TAZ to promote tumorigenesis, whereas in high‐grade serous ovarian cancer, ET‐1 signaling cooperates with YAP‐associated transcriptional programs to support invasive plasticity [18, 19]. Additional ovarian cancer studies further indicate that endothelin‐dependent tumor–stroma communication can promote fibroblast‐driven invasion, stromal feed‐forward loops, and invadopodia formation [28, 29]. Our findings extend this endothelin–YAP concept to TNBC but define a distinct Gαq/11–Rho/ROCK–LATS mechanism and an enhancer‐dependent EDNRA transcriptional feedback loop. This suggests that endothelin–YAP signaling is not a fixed pathway but a modular oncogenic architecture that can be rewired according to tumor lineage and microenvironmental state.

The enhancer‐associated feedback component is an important mechanistic observation. YAP/TAZ‐driven transcription is shaped by enhancer landscapes and TEAD‐centered enhancer occupancy [30, 31]. Functional epigenomic studies also indicate that YAP‐responsive elements are highly context dependent, which may explain why the same Hippo effector drives different oncogenic programs across cancer types [30, 31]. In our model, ET‐1/EDNRA signaling suppresses LATS‐mediated YAP phosphorylation and promotes YAP nuclear activity; activated YAP/TEAD4 then reinforces EDNRA transcription through a candidate enhancer‐associated regulatory region. This suggests that EDNRA may function not only as a receptor input but also as part of a YAP‐regulated amplification circuit. Similar regulatory logic has been observed in cancer enhancer biology, where enhancer reprogramming stabilizes malignant transcriptional identity [32, 33]. The EDNRA–YAP loop may therefore help TNBC cells transform intermittent extracellular endothelin stimulation into a sustained YAP‐dependent state.

This model also provides a biological explanation for the stem‐like and migratory phenotypes observed in our study. YAP/TAZ have been repeatedly linked to cancer stem‐like traits, epithelial–mesenchymal plasticity, extracellular matrix remodeling, and therapy resistance [26, 27]. EDNRA depletion or atrasentan treatment reduced proliferative, migratory, and stem‐like phenotypes, whereas ET‐1 stimulation and EDNRA overexpression had the opposite effects. Because EDNRA expression was associated with angiogenesis, invasion, hypoxia, metastasis, and stemness‐related signatures in our analyses, EDNRA may sit at the convergence of multiple aggressive programs. This is analogous to how inflammatory receptors can coordinate chronic inflammatory signaling with oncogenic transcriptional networks in other tumors; in TNBC, the corresponding environmental framework may involve endothelin‐rich, hypoxic, vascular, and stromal niches rather than classical chronic inflammation alone.

From a translational perspective, the EDNRA–YAP/TAZ loop provides a receptor‐level entry point for targeting YAP‐driven TNBC. Direct inhibition of YAP/TAZ–TEAD remains promising but challenging, although TEAD inhibitors and YAP/TEAD‐disrupting agents have shown encouraging preclinical and early clinical progress [34, 35]. Additional agents that disrupt the YAP–TEAD interface have provided early proof of concept for drugging this transcriptional module [36]. EDNRA blockade may offer a complementary approach by suppressing a tumor‐relevant upstream input rather than broadly inhibiting nuclear Hippo output. Atrasentan is particularly relevant because it is a selective endothelin A receptor antagonist with prior clinical testing in renal disease [37, 38]. In our models, atrasentan phenocopied EDNRA depletion, reduced YAP activity, and inhibited xenograft growth, suggesting that EDNRA antagonism may disrupt upstream YAP activation and thereby weaken downstream YAP/TEAD‐dependent reinforcement of EDNRA expression. Thus, the decrease in EDNRA expression observed after atrasentan treatment is best interpreted as a feedback‐loop consequence rather than as direct receptor downregulation by the antagonist. Importantly, low‐dose atrasentan also sensitized TNBC cells to paclitaxel and produced positive zero interaction potency synergy scores, indicating that EDNRA inhibition may enhance chemotherapy response rather than acting only as a single‐agent intervention. The observed synergy between low‐dose atrasentan and paclitaxel further supports the potential value of EDNRA blockade as a chemosensitizing strategy, although in vivo combination studies and pharmacodynamic validation will be required before clinical translation.

Several limitations remain. First, our in vivo experiments were based on subcutaneous xenograft models established in immunodeficient mice. Although these models support the role of EDNRA in tumor growth, they cannot fully recapitulate orthotopic breast tumor architecture, immune interactions, stromal ET‐1 sources, or the vascular niche. Therefore, the translational implications of EDNRA blockade should be interpreted with appropriate caution. Future studies using orthotopic breast cancer models, patient‐derived organoids, patient‐derived xenografts, immune‐competent models, and pharmacodynamic analyses will be needed to more rigorously evaluate the therapeutic relevance of EDNRA inhibition. Higher‐resolution single‐cell and spatial analyses may also help define whether EDNRA–YAP activity is enriched in specific malignant, vascular, stromal, or immune niches in TNBC [39, 40]. The enhancer‐dependent regulation of EDNRA should be further mapped using assays for transposase‐accessible chromatin using sequencing (ATAC‐seq), cleavage under targets and tagmentation (CUT&Tag), enhancer deletion, or single‐cell multi‐omics. Spatial profiling studies in treatment‐naïve and residual breast cancer further support the value of resolving tumor–stroma heterogeneity when evaluating targetable signaling programs [41, 42]. Finally, EDNRA may cooperate with MAPK, phosphoinositide 3‐kinase/protein kinase B (PI3K/AKT), focal adhesion, hypoxia, and metabolic pathways, which could modify drug response. Future studies should determine whether EDNRA‐high, nuclear YAP‐high, or YAP‐signature‐high TNBC tumors are most sensitive to EDNRA blockade, validate the paclitaxel‐sensitizing effect of atrasentan in orthotopic, patient‐derived xenograft, or pharmacodynamic models, and further examine whether EDNRA antagonists can enhance immunotherapy, PARP inhibition, or antibody–drug conjugates. Taken as a whole, EDNRA coordinates extracellular endothelin signaling with nuclear YAP transcriptional activity through a positive feedback loop, providing a mechanistic explanation for sustained YAP activation and a potential therapeutic vulnerability in TNBC.

## 5 Conclusions

**FIGURE 10:**
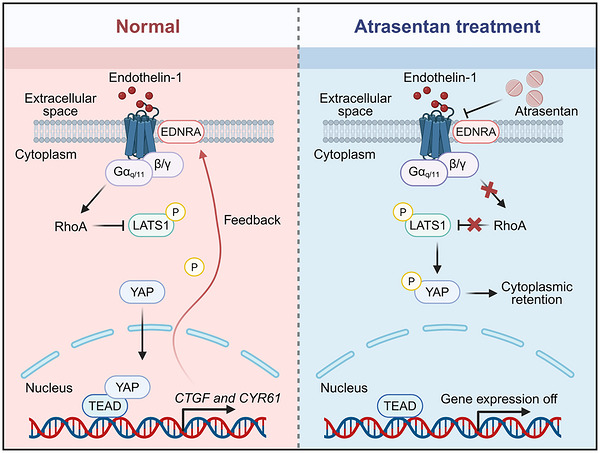
Proposed model of the EDNRA–YAP positive feedback loop and its blockade by atrasentan in TNBC. Under normal EDNRA‐active conditions, endothelin‐1 (ET‐1) binds to EDNRA and activates Gαq/11‐dependent signaling. This promotes RhoA activation and suppresses LATS1‐mediated YAP phosphorylation, allowing YAP to translocate into the nucleus. Nuclear YAP cooperates with TEAD family members to induce the expression of YAP target genes, including CTGF and CYR61, and further reinforces EDNRA transcription, thereby establishing a positive feedback loop that sustains Hippo/YAP pathway activation and promotes TNBC progression. Upon atrasentan treatment, EDNRA activation is blocked, leading to inhibition of the Gαq/11–RhoA signaling cascade and restoration of LATS1‐dependent YAP phosphorylation. Phosphorylated YAP is retained in the cytoplasm, TEAD‐dependent transcription is suppressed, and the EDNRA–YAP feedback circuit is disrupted.

These findings place EDNRA at the center of a membrane‐to‐nucleus oncogenic signaling circuit in TNBC. Rather than functioning only as a clinically associated GPCR, EDNRA acts as an upstream amplifier of YAP activity by converting ET‐1 stimulation into Gαq/11–Rho/ROCK–dependent suppression of LATS1‐mediated YAP phosphorylation. This signaling event promotes YAP nuclear accumulation and TEAD‐driven transcriptional output, thereby supporting proliferative, migratory, stem‐like and tumorigenic phenotypes in TNBC cells. Notably, EDNRA is also reinforced by YAP/TEAD4 through an enhancer‐associated regulatory mechanism, creating a self‐sustaining feedback architecture that maintains Hippo/YAP pathway activation. Pharmacological blockade with atrasentan interrupts this circuit by restoring YAP phosphorylation, limiting nuclear YAP signaling, and suppressing EDNRA‐associated malignant phenotypes (Figure 10). Together, these results support the EDNRA–YAP feedback loop as a mechanistically important contributor to TNBC malignant behavior and highlight EDNRA as a therapeutically accessible vulnerability for restraining YAP‐dependent tumor behavior.

## Author Contributions

Conceptualization: ZH, BL, YC. Methodology: ZH, BL, JX, RL, BZ. Software: BL, LYL. Validation: ZH, JX, RL, JP, CF. Formal analysis: ZH, BL, JX, LYL, RJA, EAW. Investigation: ZH, BL, JX, RL, JP, CF, BZ. Resources: BZ, YC. Data curation: BL, ZH, JX, LYL, EAW. Writing – original draft: ZH, BL. Writing – review & editing: YC, ZH, BL, LYL, RJA, EAW, BZ, and all authors. Visualization: BL, ZH, JX. Supervision: YC. Project administration: YC.

## Funding

The authors have nothing to report.

## Ethics Statement

All procedures involving human tissue samples were conducted in accordance with the ethical principles of the Declaration of Helsinki. This study was approved by the Ethics Committee of the Affiliated Cancer Hospital of Xinjiang Medical University (approval no. K‐2024014). Written informed consent was obtained from all participants or their legal guardians, and all clinical information was anonymized before analysis. All animal experiments were performed in strict compliance with institutional guidelines and were approved by the Animal Ethics Committee of Xinjiang Medical University (ethics no. A240301‐194). Mice were maintained under standard conditions, and every effort was made to minimize animal suffering. At the experimental endpoint, animals were humanely euthanized according to approved procedures.

## Conflicts of Interest

The authors declare no conflicts of interest.

## Supporting information




**Supporting File 1**: advs76784‐sup‐0001‐SuppMat.docx.

**Supporting File 2**: advs76784‐sup‐0002‐Source_Data_Western_Blot_Densitometry.xlsx.

**Supporting File 3**: advs76784‐sup‐0003‐data.zip.

## Data Availability

Public datasets analyzed in this study were obtained from TCGA, GTEx, METABRIC, and GEO. TCGA‐BRCA transcriptomic and clinicopathological data were used for the initial breast cancer‐level GPCR screening, EDNRA expression analysis, clinicopathological comparisons, functional signature correlation, Hippo/YAP signature enrichment, YAP target‐gene correlation, and survival analysis. GTEx normal breast tissue data were integrated with TCGA‐BRCA for tumor‐versus‐normal expression comparison. TCGA‐TNBC samples were used for TNBC‐restricted validation of candidate GPCRs, functional signature analysis, Hippo/YAP enrichment, YAP target‐gene correlation, and survival analysis. METABRIC‐TNBC and the GEO dataset GSE58812 were used as independent TNBC validation cohorts for EDNRA prognostic analysis. The GEO dataset GSE9893_BRCA was used for distant metastasis‐related EDNRA expression analysis. TCGA‐COAD, TCGA‐LIHC, TCGA‐LUAD, and TCGA‐PAAD were used to assess the association between EDNRA expression and Hippo/YAP‐related signatures across additional cancer types. GEO datasets GSE61852 and GSE131687 were used to analyze YAP and TEAD4 chromatin occupancy around the EDNRA locus. All data generated or analyzed during this study are included in this article and/or its supplementary material files. Further inquiries can be directed to the corresponding author.
