# Supplementary material for: EDNRA Forms a Positive Feedback Loop with the Hippo/YAP Axis to Drive Triple‐Negative Breast Cancer Progression
Source: Adv Sci (Weinh). 2026 Jul 24;13(58):e76784. doi: 10.1002/advs.76784 (PMC13398129; doi:10.1002/advs.76784)
Supplement: Supplementary file 1 [file ADVS-13-e76784-s002.docx]

**Supplementary Figure S1. TCGA-TNBC subtype-restricted analysis supports EDNRA as a Hippo/YAP-associated GPCR candidate.**

A. Schematic workflow of the TNBC-specific re-analysis of the 15 candidate GPCRs identified from the initial druggability-oriented screen. Candidate GPCRs were evaluated in TCGA-TNBC samples by gene set enrichment analysis using the CORDENONSI_YAP_CONSERVED_SIGNATURE and by correlation analysis with combined Hippo/YAP signature z-scores. Candidates meeting both criteria, normalized enrichment score greater than 2 and correlation coefficient greater than 0.3, are shown on the right. EDNRA remained a qualified TNBC-associated candidate.

B. Summary table showing the normalized enrichment score and correlation coefficient for each of the 15 candidate GPCRs in TCGA-TNBC samples. Red values indicate that the corresponding criterion was met. EDNRA showed a normalized enrichment score of 2.288 and a correlation coefficient of 0.45.

**Supplementary Figure S2. EDNRA expression is positively associated with Hippo/YAP signatures in multiple cancer types.**

A. Gene set enrichment analysis (GSEA) plots showing enrichment of the CORDENONSI_YAP_CONSERVED_SIGNATURE gene set in EDNRA-high versus EDNRA-low samples from colon adenocarcinoma (COAD), liver hepatocellular carcinoma (LIHC), lung adenocarcinoma (LUAD), and pancreatic adenocarcinoma (PAAD) cohorts.

B. GSEA plots showing enrichment of the REACTOME_SIGNALING_BY_HIPPO gene set in EDNRA-high versus EDNRA-low samples from COAD, LIHC, LUAD, and PAAD cohorts.

**Supplementary Figure S3. EDNRA expression across TNBC cell lines and validation of EDNRA knockdown efficiency.**

A. Western blot analysis of EDNRA protein expression in a panel of TNBC cell lines, including BT549, MDA-MB-231, MDA-MB-157, HCC1806, Hs578T, SUM149PT, MDA-MB-436, CAL-51, MDA-MB-468, and HCC1937. β-Actin was used as the loading control. Cell lines selected for subsequent functional experiments are highlighted in red.

B. Reverse transcription quantitative polymerase chain reaction (RT-qPCR) analysis of EDNRA mRNA expression across the indicated TNBC cell lines.

C, D. RT-qPCR analysis of EDNRA mRNA expression in MDA-MB-231 and HCC1806 cells transfected or infected with siControl, shControl, siEDNRA, or shEDNRA. Both siRNA-mediated and shRNA-mediated EDNRA depletion efficiently reduced EDNRA expression, whereas no significant difference was observed between siControl and shControl or between siEDNRA and shEDNRA groups.

**Supplementary Figure S4. EDNRA depletion suppresses malignant phenotypes in HCC1806 TNBC cells.**

A. Western blot analysis confirming EDNRA knockdown efficiency in HCC1806 cells transfected with siControl, siEDNRA#1, or siEDNRA#2. β-Actin was used as the loading control.

B. Cell Counting Kit-8 (CCK-8) assay showing the proliferation of HCC1806 cells after EDNRA knockdown.

C, D. Representative Transwell migration images and quantification of migrated HCC1806 cells after EDNRA depletion.

E, F. Representative Annexin V/propidium iodide flow cytometry plots and quantification of apoptotic HCC1806 cells after EDNRA knockdown.

G, H. Representative 5-ethynyl-2′-deoxyuridine (EdU) staining images and quantification of EdU-positive HCC1806 cells after EDNRA depletion (Hoechst 33342, blue; EdU, red).

**Supplementary Figure S5. Atrasentan inhibits EDNRA expression and malignant phenotypes in HCC1806 TNBC cells.**

A. Western blot analysis of EDNRA protein expression in HCC1806 cells treated with increasing concentrations of atrasentan. β-Actin was used as the loading control.

B. Cell Counting Kit-8 (CCK-8) assay showing the proliferation of HCC1806 cells treated with 0, 2, or 4 μM atrasentan.

C, D. Representative Transwell migration images and quantification of migrated HCC1806 cells after atrasentan treatment.

E, F. Representative Annexin V/propidium iodide flow cytometry plots and quantification of apoptotic HCC1806 cells after atrasentan treatment.

G, H. Representative EdU staining images and quantification of EdU-positive HCC1806 cells after atrasentan treatment (Hoechst 33342, blue; EdU, red).

**Supplementary Figure S6. ET-1 stimulation enhances EDNRA expression and malignant phenotypes in HCC1806 TNBC cells.**

A. Western blot analysis of EDNRA protein expression in HCC1806 cells treated with 0, 50, or 100 nM endothelin-1 (ET-1). β-Actin was used as the loading control.

B. Cell Counting Kit-8 (CCK-8) assay showing the proliferation of HCC1806 cells treated with 0, 50, or 100 nM ET-1.

C, D. Representative Transwell migration images and quantification of migrated HCC1806 cells after ET-1 stimulation.

E, F. Representative Annexin V/propidium iodide flow cytometry plots and quantification of apoptotic HCC1806 cells after ET-1 stimulation.

G, H. Representative EdU staining images and quantification of EdU-positive HCC1806 cells after ET-1 stimulation (Hoechst 33342, blue; EdU, red).

**Supplementary Figure S7. EDNRA overexpression promotes malignant phenotypes in MDA-MB-231 and HCC1806 TNBC cells.**

A, B. Western blot analysis confirming Flag-EDNRA overexpression in MDA-MB-231 and HCC1806 cells transfected with increasing amounts of Flag-EDNRA plasmid. β-Actin was used as the loading control.

C, D. Cell Counting Kit-8 (CCK-8) assays showing the proliferation of MDA-MB-231 and HCC1806 cells after Flag-EDNRA overexpression.

E–H. Representative Transwell migration images and quantification of migrated MDA-MB-231 and HCC1806 cells after Flag-EDNRA overexpression.

I–L. Representative Annexin V/propidium iodide flow cytometry plots and quantification of apoptotic MDA-MB-231 and HCC1806 cells after Flag-EDNRA overexpression.

M–P. Representative EdU staining images and quantification of EdU-positive proliferating cells in MDA-MB-231 and HCC1806 cells after Flag-EDNRA overexpression (Hoechst 33342, blue; EdU, red).

**Supplementary Figure S8. EDNRA regulates Hippo/YAP/TAZ signaling in TNBC cells.**

A. Western blot analysis of EDNRA, phosphorylated YAP at Ser127, and total YAP expression in HCC1806 cells after siRNA-mediated EDNRA depletion. β-Actin was used as the loading control.

B. Western blot analysis of EDNRA, phosphorylated YAP at Ser127, and total YAP expression in HCC1806 cells treated with 0, 2, or 4 μM atrasentan. β-Actin was used as the loading control.

C. Reverse transcription quantitative polymerase chain reaction (RT-qPCR) analysis of CYR61 and CTGF mRNA expression in HCC1806 cells after EDNRA knockdown.

D. RT-qPCR analysis of CYR61 and CTGF mRNA expression in HCC1806 cells after atrasentan treatment.

E. Western blot analysis of Flag, phosphorylated YAP at Ser127, and total YAP expression in HCC1806 cells transfected with increasing amounts of Flag-EDNRA plasmid. β-Actin was used as the loading control.

F. Western blot analysis of EDNRA, phosphorylated YAP at Ser127, and total YAP expression in HCC1806 cells treated with 0, 50, or 100 nM endothelin-1 (ET-1). β-Actin was used as the loading control.

G. RT-qPCR analysis of CYR61 and CTGF mRNA expression in HCC1806 cells after Flag-EDNRA overexpression.

H. RT-qPCR analysis of CYR61 and CTGF mRNA expression in HCC1806 cells after ET-1 stimulation.

I. TEA domain transcription factor (TEAD) luciferase reporter assay showing the effect of EDNRA knockdown on TEAD transcriptional activity in HCC1806 cells.

J. TEAD luciferase reporter assay showing the effect of atrasentan treatment on TEAD transcriptional activity in HCC1806 cells.

K. TEAD luciferase reporter assay showing the effect of Flag-EDNRA overexpression on TEAD transcriptional activity in HCC1806 cells.

L. TEAD luciferase reporter assay showing the effect of ET-1 stimulation on TEAD transcriptional activity in HCC1806 cells.

M, N. Representative immunofluorescence images and quantification of YAP subcellular localization in HCC1806 cells treated with DMSO or atrasentan. DAPI was used for nuclear staining. Scale bar, 10 μm.

O, P. Representative immunofluorescence images and quantification of YAP subcellular localization in HCC1806 cells treated with vehicle or ET-1. DAPI was used for nuclear staining. Scale bar, 10 μm.

Q, R. Western blot analysis of EDNRA, LATS2, TAZ, and phosphorylated TAZ at Ser89 in MDA-MB-231 and HCC1806 cells after EDNRA knockdown. β-Actin was used as the loading control.

S, T. Western blot analysis of Flag, LATS2, TAZ, and phosphorylated TAZ at Ser89 in MDA-MB-231 and HCC1806 cells after Flag-EDNRA overexpression. β-Actin was used as the loading control.

**Supplementary Figure S9. YAP re-expression rescues the suppressive effects caused by EDNRA depletion.**

A, B. Western blot analysis confirming EDNRA knockdown and Myc-YAP overexpression in MDA-MB-231 and HCC1806 cells. β-Actin was used as the loading control.

C, D. Reverse transcription quantitative polymerase chain reaction (RT-qPCR) analysis of EDNRA mRNA expression in MDA-MB-231 and HCC1806 cells transfected with siControl + Myc, siEDNRA + Myc, or siEDNRA + Myc-YAP.

E, F. RT-qPCR analysis of CYR61 and CTGF mRNA expression in MDA-MB-231 and HCC1806 cells after EDNRA depletion with or without Myc-YAP overexpression.

G, H. TEA domain transcription factor (TEAD) luciferase reporter assays showing the effect of Myc-YAP overexpression on TEAD transcriptional activity in EDNRA-depleted MDA-MB-231 and HCC1806 cells.

I, J. Cell Counting Kit-8 (CCK-8) assays showing the proliferation of MDA-MB-231 and HCC1806 cells after EDNRA depletion with or without Myc-YAP overexpression.

K–N. Representative Transwell migration images and quantification of migrated MDA-MB-231 and HCC1806 cells after EDNRA depletion with or without Myc-YAP overexpression.

O–R. Representative Annexin V/propidium iodide flow cytometry plots and quantification of apoptotic MDA-MB-231 and HCC1806 cells after EDNRA depletion with or without Myc-YAP overexpression.

S–V. Representative EdU staining images and quantification of EdU-positive proliferating cells in MDA-MB-231 and HCC1806 cells after EDNRA depletion with or without Myc-YAP overexpression (Hoechst 33342, blue; EdU, red).

W–Y. Representative xenograft tumor images, tumor weight, and tumor volume in the shControl, shEDNRA, and shEDNRA + YAP groups. For xenograft experiments, n = 5 mice per group.

**Supplementary Figure S10. YAP depletion attenuates EDNRA overexpression-induced malignant phenotypes.**

A. Western blot analysis confirming Flag-EDNRA overexpression and YAP knockdown in MDA-MB-231 cells. β-Actin was used as the loading control.

B. RT-qPCR analysis of EDNRA mRNA expression in MDA-MB-231 cells transfected with Flag + siControl, Flag-EDNRA + siControl, or Flag-EDNRA + siYAP.

C. RT-qPCR analysis of CTGF and CYR61 mRNA expression in MDA-MB-231 cells after Flag-EDNRA overexpression with or without YAP knockdown.

D. TEAD luciferase reporter assay showing the effect of YAP knockdown on TEAD transcriptional activity induced by Flag-EDNRA overexpression.

E. CCK-8 assay showing the proliferation of MDA-MB-231 cells after Flag-EDNRA overexpression with or without YAP knockdown.

F, G. Representative Transwell migration images and quantification of migrated MDA-MB-231 cells after Flag-EDNRA overexpression with or without YAP knockdown.

H, I. Representative Annexin V/propidium iodide flow cytometry plots and quantification of apoptotic MDA-MB-231 cells after Flag-EDNRA overexpression with or without YAP knockdown.

**Supplementary Figure S11. Dose–response matrix, ZIP synergy landscape, and combination index analysis of paclitaxel plus atrasentan treatment in TNBC cells.**

A, B. Drug-combination response matrices showing the inhibition rates of MDA-MB-231 and HCC1806 cells treated with increasing concentrations of paclitaxel (PTX) and atrasentan. Atrasentan concentrations ranged from 0 to 4000 nM, and PTX concentrations ranged from 0 to 100 nM. The color intensity represents the inhibition percentage for each drug-combination condition.

C, D. Three-dimensional zero interaction potency (ZIP) synergy landscapes showing the interaction between PTX and atrasentan in MDA-MB-231 and HCC1806 cells. Positive ZIP scores indicate synergistic interactions between the two drugs. The overall ZIP synergy scores were 13.69 for MDA-MB-231 cells and 12.392 for HCC1806 cells.

E, F. Chou–Talalay combination index (CI) plots showing the interaction between PTX and atrasentan in MDA-MB-231 and HCC1806 cells. CI values below 1 indicate synergism. The CI values at ED50 were 0.503 for MDA-MB-231 cells and 0.497 for HCC1806 cells.

**Supplementary Table S1. Sequences of siGPCRs screen.**

| Target gene | siRNA name | Sense strand (5'-3') | Antisense strand (5'-3') |
| --- | --- | --- | --- |
| Control | siControl | UUCUCCGAACGUGUCACGUTT | ACGUGACACGUUCGGAGAATT |
| AVPR1A | siAVPR1A | CCUACAUGCUGGUAGUCAUTT | AUGACUACCAGCAUGUAGGTT |
| CCR4 | siCCR4 | GGACCUUGCAGCAUUGUAATT | UUACAAUGCUGCAAGGUCCTT |
| CHRM3 | siCHRM3 | GCAGCAGUUACGAACUUCATT | UGAAGUUCGUAACUGCUGCTT |
| CYSLTR2 | siCYSLTR2 | CUUCAGGGCUGACUAUUAUTT | AUAAUAGUCAGCCCUGAAGTT |
| EDNRA | siEDNRA | CUCCCUAUCAAUGUAUUUATT | UAAAUACAUUGAUAGGGAGTT |
| F2R | siF2R | GGCAGUUGAUGGCAAGUAATT | UUACUUGCCAUCAACUGCCTT |
| GLP1R | siGLP1R | GCAACUACAUCCUGAUCAATT | UUGAUCAGGAUGUAGUUGCTT |
| HRH1 | siHRH1 | GGCCUGGAUUACAUCAAGUTT | ACUUGAUGUAAUCCAGGCCTT |
| HRH2 | siHRH2 | AUCGUGUCCUUGGCUAUCATT | UGAUAGCCAAGGACACGAUTT |
| HTR1B | siHTR1B | CCAAGGACUACAUUUACCATT | UGGUAAAUGUAGUCCUUGGTT |
| HTR2A | siHTR2A | GGGCCAAAUUAGCUUCUUUTT | AAAGAAGCUAAUUUGGCCCTT |
| P2RY1 | siP2RY1 | GCGGGAGAUACUUUCAGAATT | UUCUGAAAGUAUCUCCCGCTT |
| PTGER4 | siPTGER4 | CAAGUUUGGAGCGAGAAGUTT | ACUUCUCGCUCCAAACUUGTT |
| PTGFR | siPTGFR | GCUACCUCAUCUUCAUCAATT | UUGAUGAAGAUGAGGUAGCTT |
| SUCNR1 | siSUCNR1 | GUGGGAGUCCUUGGAAAUATT | UAUUUCCAAGGACUCCCACTT |

**Supplementary Table S2. Sequences.**

| Type | Name | Sequence 1, 5'-3' | Sequence 2, 5'-3' |
| --- | --- | --- | --- |
| siRNA | siControl | UUCUCCGAACGUGUCACGUTT | ACGUGACACGUUCGGAGAATT |
| siRNA | siEDNRA-1 | CUCCCUAUCAAUGUAUUUATT | UAAAUACAUUGAUAGGGAGTT |
| siRNA | siEDNRA-2 | CUGGUUCCCUCUUCAUUUATT | UAAAUGAAGAGGGAACCAGTT |
| siRNA | siYAP | GUCAGAGAUACUUCUUAAATT | UUUAAGAAGUAUCUCUGACTT |
| siRNA | siGNAQ | GUACAGUCCCAGCACAUUUTT | AAAUGUGCUGGGACUGUACTT |
| siRNA | siGNA11 | AAAGGGUACUCGAUGAUGCTT | GCAUCAUCGAGUACCCUUUTT |
| siRNA | siGNAI1 | CCAUGAAGCUGGUUAUUCATT | UGAAUAACCAGCUUCAUGGTT |
| siRNA | siGNAI2 | AUCGUCAAGCAGAUGAAGATT | UCUUCAUCUGCUUGACGAUTT |
| siRNA | siGNAI3 | GCAGGAGUGAUUAAACGGUTT | ACCGUUUAAUCACUCCUGCTT |
| shRNA | shControl | CCGGCAACAAGATGAAGAGCACCAACTCGAGTTGGTGCTCTTCATCTTGTTGTTTTTG | AATTCAAAAACAACAAGATGAAGAGCACCAACTCGAGTTGGTGCTCTTCATCTTGTTG |
| shRNA | shEDNRA | CCGGCCACTCATCAACCCACTAATTCTCGAGAATTAGTGGGTTGATGAGTGGTTTTTG | AATTCAAAAACCACTCATCAACCCACTAATTCTCGAGAATTAGTGGGTTGATGAGTGG |
| sgRNA | sgControl | CACCGCGAGGTATTCGGCTCCGCG | AAACCGCGGAGCCGAATACCTCGCC |
| sgRNA | sgEDNRA-Promoter | CACCGCGCGGAGGAGGGCGAGGAG | AAACCTCCTCGCCCTCCTCCGCGCC |
| sgRNA | sgEDNRA-Enhancer | CACCGCTGAGACAGTGAGACTGCA | AAACTGCAGTCTCACTGTCTCAGCC |

**Supplementary Table S3. Chemicals.**

| Chemical / reagent | Abbreviation in figures | Target / purpose | Manufacturer | Catalog No. |
| --- | --- | --- | --- | --- |
| Atrasentan hydrochloride | Atrasentan | Selective EDNRA antagonist | MedChemExpress | HY-15403A |
| Endothelin 1, swine/human | ET-1 | Endogenous EDNRA ligand | MedChemExpress | HY-P0202 |
| Verteporfin | VP | YAP–TEAD transcriptional inhibitor | MedChemExpress | HY-B0146 |
| XMU-MP-1 | XMU-MP-1 | MST1/2 inhibitor; YAP pathway activator | MedChemExpress | HY-100526 |
| Exoenzyme C3, Clostridium botulinum | C3 | Rho inhibitor | MedChemExpress | HY-P2325 |
| GSK429286A | GSK429286 | ROCK inhibitor | MedChemExpress | HY-11000 |
| Y-27632 dihydrochloride | Y27632 | ROCK inhibitor | MedChemExpress | HY-10583 |
| Dimethyl sulfoxide | DMSO | Vehicle control | Sigma-Aldrich | D2650 |

**Supplementary Table S4. Primer sequences for RT-qPCR.**

| Gene symbol | Forward primer (5'-3') | Reverse primer (5'-3') |
| --- | --- | --- |
| EDNRA | CGATTGGCTTCGTCATGGTAC | GATCGCAGTGCACACCAAG |
| CTGF | ACCGACTGGAAGACACGTTTG | CCAGGTCAGCTTCGCAAGG |
| CYR61 | GGTCAAAGTTACCGGGCAGT | GGAGGCATCGAATCCCAGC |
| YAP | CAAGAAAGCAGGCTCACAGAA | GCTGGGTGTTAGGGCTTCG |

**Supplementary Table S5. Antibodies.**

| Antibody | Manufacturer | Catalog No. |
| --- | --- | --- |
| Anti-EDNRA | Abcam | ab117521 |
| Anti-YAP | Cell Signaling Technology | 14074 |
| Anti-phospho-YAP Ser127 | Cell Signaling Technology | 13008 |
| Anti-LATS1 | Cell Signaling Technology | 3477 |
| Anti-Gαq/11 | Santa Cruz Biotechnology | sc-515689 |
| Anti-Gαi | Cell Signaling Technology | 5290 |
| Anti-Flag M2 | Sigma-Aldrich / MilliporeSigma | F1804 |
| Anti-Myc tag | Cell Signaling Technology | 2276 |
| Anti-Ki-67 | Cell Signaling Technology | 9449 |
| PE Mouse Anti-Human CD24 | BD Pharmingen / BD Biosciences | 555428 |
| FITC Mouse Anti-Human CD44 | BD Pharmingen / BD Biosciences | 555478 |
| Anti-β-Actin | Proteintech | 66009-1-Ig |
| Anti-α-Tubulin | Proteintech | 66031-1-Ig |
| Anti-Histone H3 | Cell Signaling Technology | 4499 |
| Normal Rabbit IgG | Cell Signaling Technology | 2729 |
| Mouse IgG1 isotype control | Cell Signaling Technology | 5415 |
| HRP-linked anti-rabbit IgG | Cell Signaling Technology | 7074 |
| HRP-linked anti-mouse IgG | Cell Signaling Technology | 7076 |
| Goat anti-rabbit IgG H&L, Alexa Fluor 488 | Invitrogen / Thermo Fisher Scientific | A-11008 |
| Goat anti-mouse IgG H&L, Alexa Fluor 488 | Invitrogen / Thermo Fisher Scientific | A-11001 |
| LATS2 | Cell Signaling Technology | #5888 |
| TAZ | Cell Signaling Technology | #83669 |
| p-TAZ Ser89 | Invitrogen / Thermo Fisher Scientific | PA5-105066 |
