# Supplementary material for: EDNRA Forms a Positive Feedback Loop with the Hippo/YAP Axis to Drive Triple‐Negative Breast Cancer Progression
Source: Adv Sci (Weinh). 2026 Jul 24:e76784. Online ahead of print. doi: 10.1002/advs.76784 (PMC13398129; doi:10.1002/advs.76784)
Supplement: Supplementary file 3 — Supporting File 3: advs76784‐sup‐0003‐data.zip. [file ADVS-9999-e76784-s001.zip › advs76784-sup-0003-data/EDNRA row data.pdf]

Figure2A

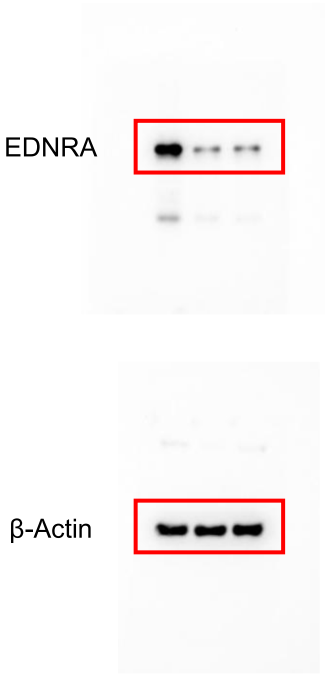

Figure2B

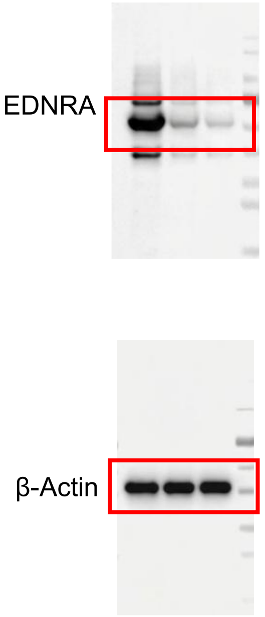

Figure2C

	siControl			siEDNRA#1			siEDNRA#2		
EDNRA	0.849687	0.996625	1.153688	0.321583	0.337779	0.325863	0.325839	0.351687	0.355057

siC	EDNRA	UNKNOW	SYBR	None				24.96257	10.54750916595460	3.05856E-08	0.849686983
siC	EDNRA	UNKNOW	SYBR	None				24.73245	10.34844775134090	3.58748E-08	0.996625294
siC	EDNRA	UNKNOW	SYBR	None				24.52132	9.92249626007377	4.15285E-08	1.153687722
siEDNRA#	EDNRA	UNKNOW	SYBR	None				26.36431	11.95058267187490	1.15758E-08	0.321583012
siEDNRA#	EDNRA	UNKNOW	SYBR	None				26.29342	11.91017507083740	1.21588E-08	0.337779116
siEDNRA#	EDNRA	UNKNOW	SYBR	None				26.34524	11.96450852538700	1.17299E-08	0.325862951
siEDNRA#	EDNRA	UNKNOW	SYBR	None				26.34535	12.24266125632960	1.1729E-08	0.325838551
siEDNRA#	EDNRA	UNKNOW	SYBR	None				26.23521	12.19508665179300	1.26594E-08	0.351686531
siEDNRA#	EDNRA	UNKNOW	SYBR	None				26.22145	12.13656383131370	1.27807E-08	0.355056912

Figure2D

	siControl			siEDNRA#1			siEDNRA#2		
EDNRA	0.96018	1.00021	1.03961	0.35788	0.30694	0.35796	0.381	0.33756	0.30544

siC	EDNRA	UNKNOW	SYBR	None				25.6544	#############	0.00041365	0.960180894
siC	EDNRA	UNKNOW	SYBR	None				25.5644	#############	0.0004309	1.000211264
siC	EDNRA	UNKNOW	SYBR	None				25.7235	#############	0.00044787	1.039607843
siEDNRA#	EDNRA	UNKNOW	SYBR	None				27.0768	#############	0.00015418	0.357881043
siEDNRA#	EDNRA	UNKNOW	SYBR	None				27.2679	#############	0.00013223	0.306941749
siEDNRA#	EDNRA	UNKNOW	SYBR	None				27.0435	#############	0.00015421	0.357958077
siEDNRA#	EDNRA	UNKNOW	SYBR	None				26.6755	#############	0.00016414	0.381003984
siEDNRA#	EDNRA	UNKNOW	SYBR	None				26.7876	#############	0.00014543	0.337563486
siEDNRA#	EDNRA	UNKNOW	SYBR	None				26.9766	#############	0.00013159	0.305444275

Figure2E

Time(Day	siControl			siEDNRA#1			siEDNRA#2		
0	0.99706	1.11482	0.88813	0.97934	0.89915	1.12151	1.00212	1.02442	0.97346
1	1.53288	1.58292	1.75074	1.56987	1.40583	1.37667	1.37155	1.32378	1.3811
2	5.43474	5.76153	6.09127	3.40097	3.77643	2.90523	2.66242	2.24204	3.06369
3	9.01276	9.03631	8.07655	5.10814	4.96962	5.14459	4.5966	4.6603	4.52017

Figure2F

Time(Day	siControl			siEDNRA#1			siEDNRA#2		
0	1.06434	1.07273	0.86294	1.00882	1.03971	0.95147	1.00722	1.0938	0.89899
1	4.29511	4.42937	4.06014	1.88677	1.63529	1.43677	1.84271	1.59163	1.66522
2	5.63916	5.97063	6.1049	3.67059	3.51618	3.93088	3.66234	3.69264	3.84416
3	13.1427	12.3539	12.2993	6.40735	6.47794	6.65441	6.32612	6.93218	6.97114

Figure2H

siControl			siEDNRA#1			siEDNRA#2		
60.45	57.68	64.23	34.76	32.64	28.67	38.64	35.16	31.33

Figure2J

siControl			siEDNRA#1			siEDNRA#2		
56.78	58.86	57.73	34.82	30.76	31.67	29.76	28.63	33.45

Figure2M

siControl			siEDNRA#1			siEDNRA#2		
1.12	0.9	0.98	0.38	0.45	0.41	0.42	0.32	0.47

Figure2N

siControl			siEDNRA#1			siEDNRA#2		
1.09	1.03	0.88	0.35	0.38	0.33	0.42	0.37	0.36

Figure2Q

siControl			siEDNRA#1			siEDNRA#2		
18.9	17.67	19.63	30.21	33.62	28.01	31.87	30.07	32.76

Figure2R

siControl			siEDNRA#1			siEDNRA#2		
14.34	15.37	12.68	22.23	24.63	20.17	24.13	22.76	22.02

Figure2U

siControl			siEDNRA#1			siEDNRA#2		
8.39	8.87	7.97	3.52	3.63	3.42	3.72	3.51	3.61

Figure2V

siControl			siEDNRA#1			siEDNRA#2		
7.43	7.79	7.17	3.29	2.98	3.45	3.28	3.47	3.03

Figure2X

shControl					shEDNRA				
1.184	1.029	0.806	0.753	0.714	0.342	0.281	0.225	0.188	0.156

Figure2Y

ime (Days	shControl					shEDNRA				
0	0	0	0	0	0	0	0	0	0	0
7	56.2341	61.8902	48.1563	55.4321	63.2109	35.6742	41.2389	38.9012	45.6721	33.4567
14	142.678	158.342	132.901	165.432	148.211	82.3451	91.6723	74.8901	88.2345	69.1234
21	352.411	389.235	295.678	342.109	412.563	134.432	145.678	162.342	118.901	157.211
28	721.901	545.678	492.342	588.432	682.123	298.345	342.672	215.89	226.235	312.123
35	1285.43	822.123	756.678	915.342	1084.9	382.235	425.678	458.901	272.432	405.211

Figure3A

EDNRA

β-Actin

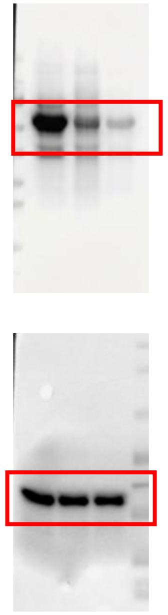

Figure3B

EDNRA

β-Actin

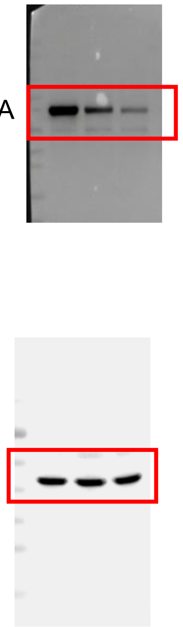

Figure3C

Atrasentan (0 μM)			Atrasentan (2 μM)			Atrasentan (4 μM)		
0.92578	0.88177	1.19245	0.38963	0.55278	0.43341	0.31747	0.29844	0.32513

EDNRA	UNKNOV	SYBR	None				24.02086105	9.605798149	0.001283414	0.92578
EDNRA	UNKNOV	SYBR	None				24.06006203	9.676057434	0.001222409	0.88177
EDNRA	UNKNOV	SYBR	None				23.83942413	9.240597725	0.001653114	1.19245
EDNRA	UNKNOV	SYBR	None				25.26809349	10.85436382	0.000540146	0.38963
EDNRA	UNKNOV	SYBR	None				24.73300018	10.34975185	0.000766327	0.55278
EDNRA	UNKNOV	SYBR	None				25.08145943	10.70072975	0.000600841	0.43341
EDNRA	UNKNOV	SYBR	None				25.25250244	11.14981747	0.000440119	0.31747
EDNRA	UNKNOV	SYBR	None				25.27916908	11.23904228	0.000413724	0.29844
EDNRA	UNKNOV	SYBR	None				25.20031853	11.11542912	0.000450736	0.32513

Figure3D

Atrasentan (0 μM)			Atrasentan (2 μM)			Atrasentan (4 μM)		
1.03337	0.88632	1.08031	0.49341	0.52194	0.47029	0.28471	0.32477	0.3237

EDNRA	UNKNOV	SYBR	None				23.84387207	7.354616165	0.006109982	1.03337
EDNRA	UNKNOV	SYBR	None				23.69643211	7.576072693	0.005240526	0.88632
EDNRA	UNKNOV	SYBR	None				23.48362923	7.290527344	0.006387525	1.08031
EDNRA	UNKNOV	SYBR	None				25.2586834	8.42110939	0.002917383	0.49341
EDNRA	UNKNOV	SYBR	None				24.95394669	8.340009308	0.003086079	0.52194
EDNRA	UNKNOV	SYBR	None				25.08543968	8.490343094	0.002780687	0.47029
EDNRA	UNKNOV	SYBR	None				25.28763657	9.214400101	0.001683407	0.28471
EDNRA	UNKNOV	SYBR	None				25.10698929	9.024492645	0.001920247	0.32477
EDNRA	UNKNOV	SYBR	None				25.00673065	9.02923069	0.001913951	0.3237

Figure3E

Time (Days	Atrasentan (0 μM)			Atrasentan (2 μM)			Atrasentan (4 μM)		
0	1.02564	1.15432	0.88457	0.98451	1.05623	0.91246	0.95234	1.01246	0.82457
1	4.15678	4.38901	3.82456	1.72456	1.55623	1.48901	1.32457	1.45678	1.25634
2	6.24567	6.95623	5.92341	3.62345	3.48901	3.82457	3.25623	3.72456	2.95623
3	11.1246	12.2568	11.8246	7.62345	8.15623	7.85623	6.15623	5.82456	5.42457

Figure3F

Time (Days	Atrasentan (0 μM)			Atrasentan (2 μM)			Atrasentan (4 μM)		
0	1.05432	0.98215	1.12457	0.96541	1.02346	0.89451	0.92345	0.98451	0.81235
1	4.25678	3.94561	4.41235	1.85641	1.62451	1.54234	1.42346	1.31246	1.28457
2	6.54123	6.82345	5.95671	4.82456	4.51235	4.95641	3.12457	3.65412	2.85641
3	11.5641	12.0235	11.2457	7.85412	8.32456	7.91235	6.25412	5.91235	5.54234

Figure3H

Atrasentan (0 μM)			Atrasentan (2 μM)			Atrasentan (4 μM)		
56.42	52.98	57.53	40.51	42.62	39.52	33.42	37.32	32.16

Figure3J

Atrasentan (0 μM)			Atrasentan (2 μM)			Atrasentan (4 μM)		
55.23	52.16	54.38	40.12	43.21	41.11	32.16	33.42	35.17

Figure3M

Atrasentan (0 μM)			Atrasentan (2 μM)			Atrasentan (4 μM)		
1.11	1.01	0.88	0.47	0.52	0.53	0.33	0.37	0.32

Figure3N

Atrasentan (0 μM)			Atrasentan (2 μM)			Atrasentan (4 μM)		
1.07	1.03	0.9	0.61	0.58	0.56	0.33	0.34	0.37

Figure3Q

Atrasentan (0 μM)			Atrasentan (2 μM)			Atrasentan (4 μM)		
10.67	11.03	9.67	14.26	15.78	13.98	18.6	19.03	18.01

Figure3R

Atrasentan (0 μM)			Atrasentan (2 μM)			Atrasentan (4 μM)		
11.01	11.76	10.32	17.21	18.43	16.97	23.86	20.12	23.67

Figure3U

Atrasentan (0 μM)			Atrasentan (2 μM)			Atrasentan (4 μM)		
8.59	8.21	9.03	4.83	4.21	5.02	3.99	3.41	3.67

Figure3V

Atrasentan (0 μM)			Atrasentan (2 μM)			Atrasentan (4 μM)		
8.42	8.69	8.41	4.77	4.53	5.02	3.74	3.47	4.02

Figure3X

Vehicle					Atrasentan				
1.156	1.074	0.892	0.781	0.695	0.312	0.264	0.239	0.197	0.142

Figure3Y

Time (Days	Vehicle					Atrasentan				
0	0	0	0	0	0	0	0	0	0	0
7	52.1432	64.2561	58.9102	49.6734	61.2289	32.4561	44.1235	36.8902	40.2134	38.5672
14	138.564	162.109	145.432	158.901	132.678	75.4321	86.9123	92.4561	68.3245	81.1092
21	324.678	405.123	368.901	312.457	389.234	148.211	155.678	128.432	162.901	136.564
28	685.432	512.678	624.901	556.342	712.109	225.678	324.123	242.901	215.432	205.678
35	1156.9	995.432	1024.12	1048.68	1232.46	305.123	452.901	398.678	295.432	272.109

Figure4A

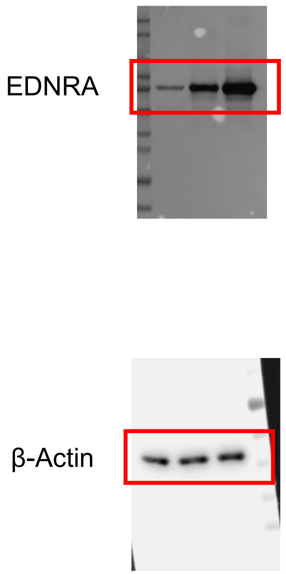

Figure4B

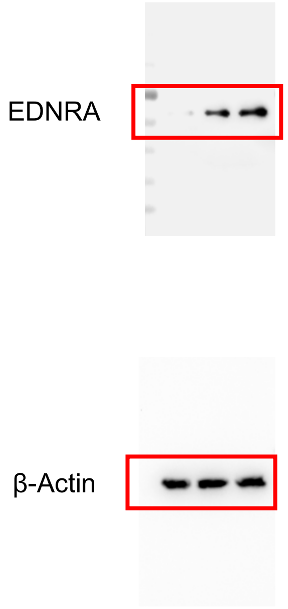

Figure4C

ET-1 (0 nM)			ET-1 (50 nM)			ET-1 (100 nM)		
0.98802	1.02958	0.9824	2.48028	2.00755	1.84122	4.58195	4.5772	3.79356

Sample Na	Target Nai	Task	Reporter	Quencher	RQ	RQ Min	RQ Max	CT	231		0.000199901
0	EDNRA	UNKNOWI	SYBR	None				27.390	12.30581056213380	0.000197507	0.988024208
0	EDNRA	UNKNOWI	SYBR	None				27.358	12.24637745666500	0.000205813	1.029576765
0	EDNRA	UNKNOWI	SYBR	None				27.281	12.31404782104490	0.000196382	0.982399027
50	EDNRA	UNKNOWI	SYBR	None				27.213	10.97792419433590	0.00049581	2.480282866
50	EDNRA	UNKNOWI	SYBR	None				27.375	11.28298970031740	0.000401312	2.007554505
50	EDNRA	UNKNOWI	SYBR	None				27.232	11.40776422119140	0.000368062	1.841223341
100	EDNRA	UNKNOWI	SYBR	None				25.233	10.09246710205080	0.000915935	4.581950128
100	EDNRA	UNKNOWI	SYBR	None				25.187	10.09396327209470	0.000914986	4.577200806
100	EDNRA	UNKNOWI	SYBR	None				25.347	10.36487728881840	0.000758335	3.79355793

Figure4D

ET-1 (0 nM)			ET-1 (50 nM)			ET-1 (100 nM)		
1.1101	0.95645	0.93344	2.16317	1.85449	1.75284	4.26407	3.27648	3.59794

0	EDNRA	UNKNOWI	SYBR	None				30.565	15.3339948120117	2.42107E-05	1.110102462
0	EDNRA	UNKNOWI	SYBR	None				30.656	15.5489200286865	2.08597E-05	0.956454091
0	EDNRA	UNKNOWI	SYBR	None				30.563	15.5840531311035	2.03579E-05	0.933443447
50	EDNRA	UNKNOWI	SYBR	None				29.474	14.3715389709473	4.71776E-05	2.163172452
50	EDNRA	UNKNOWI	SYBR	None				29.645	14.5936624755859	4.04455E-05	1.854493459
50	EDNRA	UNKNOWI	SYBR	None				29.882	14.6749898223877	3.82286E-05	1.752844277
100	EDNRA	UNKNOWI	SYBR	None				28.466	13.3924573669434	9.29969E-05	4.264067536
100	EDNRA	UNKNOWI	SYBR	None				28.857	13.7725425872803	7.14581E-05	3.276476236
100	EDNRA	UNKNOWI	SYBR	None				28.614	13.6375177612305	7.8469E-05	3.597936922

Figure4E

Times (Days	ET-1 (0 nM)			ET-1 (50 nM)			ET-1 (100 nM)		
0	0.93245	1.05671	1.01084	0.96541	1.08235	0.95224	0.91246	1.12453	0.96301
1	2.15433	2.32109	2.20457	2.45123	2.51235	2.3891	3.45612	3.61246	3.48975
2	5.86421	6.12355	5.9521	6.45123	6.78211	6.32146	8.41257	8.75612	8.39846
3	8.12454	8.87654	9.34561	13.7346	12.9823	12.1543	14.3215	14.8561	14.4123

Figure4F

Times (Days	ET-1 (0 nM)			ET-1 (50 nM)			ET-1 (100 nM)		
0	0.96345	1.02457	1.01198	0.97226	0.98542	1.04232	1.03322	1.01246	0.95432
1	2.24512	2.15643	2.43845	2.6364	2.81236	2.80125	3.65908	3.68451	3.75641
2	4.68451	5.01235	4.58314	6.07133	6.12454	6.25413	7.56333	6.52412	7.71255
3	8.05123	8.19451	8.63425	11.1944	11.2846	12.421	14.8244	13.8541	14.0215

Figure4H

ET-1 (0 nM)			ET-1 (50 nM)			ET-1 (100 nM)		
38.79	40.63	35.42	55.67	53.97	58.99	66.78	67.72	62.91

Figure4J

ET-1 (0 nM)			ET-1 (50 nM)			ET-1 (100 nM)		
41.27	38.38	43.65	55.74	57.89	56.72	63.45	66.51	59.32

Figure4M

ET-1 (0 nM)			ET-1 (50 nM)			ET-1 (100 nM)		
1.14	0.89	0.97	1.89	2.07	2.18	2.67	2.87	3.01

Figure4N

ET-1 (0 nM)			ET-1 (50 nM)			ET-1 (100 nM)		
1.12	0.93	0.96	1.58	1.47	1.62	2.01	1.95	2.08

Figure4Q

ET-1 (0 nM)			ET-1 (50 nM)			ET-1 (100 nM)		
16.21	15.48	16.77	6.78	7.68	6.04	4.5	5.32	3.86

Figure4R

ET-1 (0 nM)			ET-1 (50 nM)			ET-1 (100 nM)		
17.21	16.23	15.36	6.08	7.32	7.21	3.17	4.21	3.86

Figure4U

ET-1 (0 nM)			ET-1 (50 nM)			ET-1 (100 nM)		
9.74	8.93	10.07	15.02	14.58	15.37	20.01	19.04	20.76

Figure4V

ET-1 (0 nM)			ET-1 (50 nM)			ET-1 (100 nM)		
7.04	7.21	7.17	11.5	11.23	11.74	13.55	13.91	13.16

Figure4X

Vehicle					ET-1				
0.984	0.852	1.016	0.763	0.885	1.624	1.412	1.587	1.356	1.521

Figure4Y

Time (Days	Vehicle					ET-1				
0	0	0	0	0	0	0	0	0	0	0
7	45.3214	52.6781	48.9012	41.5643	55.2341	117.577	115.21	80.2701	103.651	86.1375
14	115.432	138.901	122.567	142.109	108.678	291.807	276.378	255.877	264.842	321.662
21	285.678	342.123	298.456	265.901	312.432	707.449	768.888	708.614	787.782	694.315
28	562.109	485.432	512.678	498.901	588.457	1119.27	1604.27	1283.84	1170.17	1219.67
35	924.567	756.901	884.234	812.678	965.432	1780.61	1787.36	1685.25	1787.11	2096.53

Figure5A

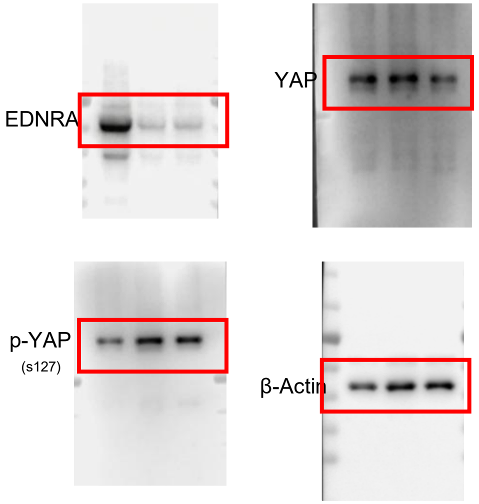

Figure5B

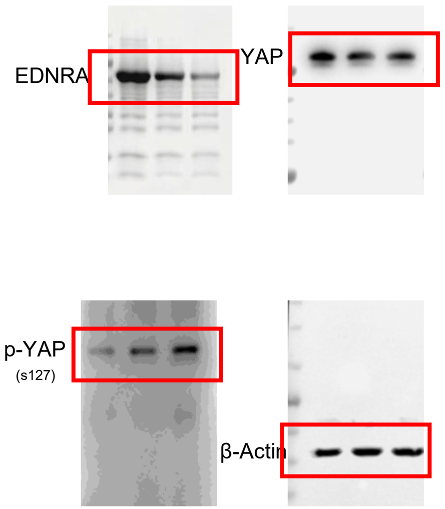

Figure5C

	siControl			siEDNRA#1			siEDNRA#2		
CYR61	0.953907	1.030072	1.016021	0.489011	0.417736	0.356014	0.397039	0.538244	0.492868
CTGF	1.007	1.001854	0.991146	0.457442	0.404939	0.385094	0.43138	0.49408	0.438797

Sample Na	Target Nar	Task	Reporter	Quencher	RQ	RQ Min	RQ Max	CT			0.082256
siControl	CYR61	UNKNOWI	SYBR	None				18.993	3.67181968688965	0.078464	0.953907
siControl	CYR61	UNKNOWI	SYBR	None				18.985	3.56099510192871	0.084729	1.030072
siControl	CYR61	UNKNOWI	SYBR	None				18.978	3.58080959320068	0.083574	1.016021
siEDNRA#	CYR61	UNKNOWI	SYBR	None				19.677	4.63580093383784	0.040224	0.489011
siEDNRA#	CYR61	UNKNOWI	SYBR	None				19.740	4.86307544708252	0.034361	0.417736
siEDNRA#	CYR61	UNKNOWI	SYBR	None				19.900	5.09373474121098	0.029284	0.356014
siEDNRA#	CYR61	UNKNOWI	SYBR	None				19.910	4.93638610839839	0.032659	0.397039
siEDNRA#	CYR61	UNKNOWI	SYBR	None				19.516	4.49740829467774	0.044274	0.538244
siEDNRA#	CYR61	UNKNOWI	SYBR	None				19.564	4.62446823120122	0.040541	0.492868
											0.06848
siControl	CTGF	UNKNOWI	SYBR	None				19.179	3.85811614990234	0.068959	1.007
siControl	CTGF	UNKNOWI	SYBR	None				19.290	3.86550712585449	0.068607	1.001854
siControl	CTGF	UNKNOWI	SYBR	None				19.278	3.88100910186768	0.067873	0.991146
siEDNRA#	CTGF	UNKNOWI	SYBR	None				20.038	4.99651718139644	0.031326	0.457442
siEDNRA#	CTGF	UNKNOWI	SYBR	None				20.049	5.17240200042722	0.02773	0.404939
siEDNRA#	CTGF	UNKNOWI	SYBR	None				20.052	5.24489784240718	0.026371	0.385094
siEDNRA#	CTGF	UNKNOWI	SYBR	None				20.054	5.08114814758301	0.029541	0.43138
siEDNRA#	CTGF	UNKNOWI	SYBR	None				19.904	4.88536148071294	0.033834	0.49408
siEDNRA#	CTGF	UNKNOWI	SYBR	None				19.996	5.05655509948732	0.030049	0.438797

Figure5D

	DMSO			Atrasentan 2 μmol/L			Atrasentan 4 μmol/L		
CYR61	0.970321	1.04619	0.983489	0.636575	0.575424	0.577801	0.383422	0.334954	0.362206
CTGF	0.939994	0.971671	1.088335	0.449432	0.444518	0.425855	0.323426	0.35397	0.334823

NControl	CYR61	UNKNOWI	SYBR	None				20.851	4.7182445526123	0.03799	0.970321
NControl	CYR61	UNKNOWI	SYBR	None				20.623	4.6096343994141	0.04096	1.04619
NControl	CYR61	UNKNOWI	SYBR	None				20.631	4.6987981796265	0.038505	0.983489
atrasentan	CYR61	UNKNOWI	SYBR	None				21.505	5.3263767242432	0.024923	0.636575
atrasentan	CYR61	UNKNOWI	SYBR	None				21.699	5.4720817565918	0.022529	0.575424
atrasentan	CYR61	UNKNOWI	SYBR	None				21.573	5.4661354064941	0.022622	0.577801
atrasentan	CYR61	UNKNOWI	SYBR	None				22.625	6.0577754974365	0.015012	0.383422
atrasentan	CYR61	UNKNOWI	SYBR	None				22.886	6.2527427673340	0.013114	0.334954
atrasentan	CYR61	UNKNOWI	SYBR	None				22.756	6.1398971557618	0.014181	0.362206
											0.156903
NControl	CTGF	UNKNOWI	SYBR	None				18.894	2.76132774353027	0.147488	0.939994
NControl	CTGF	UNKNOWI	SYBR	None				18.726	2.71351051330566	0.152459	0.971671
NControl	CTGF	UNKNOWI	SYBR	None				18.483	2.54992771148682	0.170764	1.088335
atrasentan	CTGF	UNKNOWI	SYBR	None				20.005	3.82587432861329	0.070518	0.449432
atrasentan	CTGF	UNKNOWI	SYBR	None				20.068	3.84173545837398	0.069746	0.444518
atrasentan	CTGF	UNKNOWI	SYBR	None				20.011	3.90361785888669	0.066818	0.425855
atrasentan	CTGF	UNKNOWI	SYBR	None				20.868	4.30054092407227	0.050747	0.323426
atrasentan	CTGF	UNKNOWI	SYBR	None				20.803	4.17035293579102	0.055539	0.35397
atrasentan	CTGF	UNKNOWI	SYBR	None				20.866	4.25057983398437	0.052535	0.334823

Figure5E

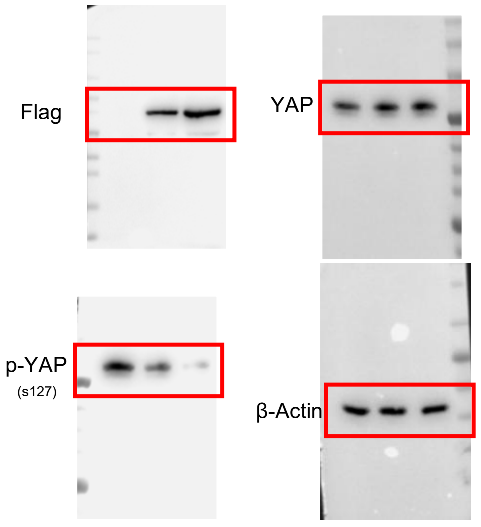

Figure5F

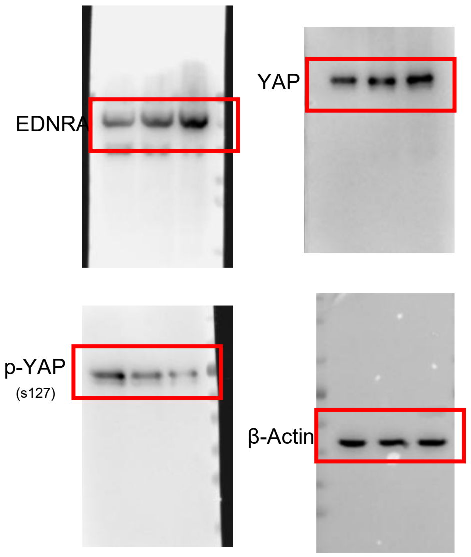

Figure5G

	Flag			Flag-EDNRA 0.5 μg			Flag-EDNRA 1 μg		
CYR61	0.928323	0.900189	1.171488	2.824913	2.495029	2.926187	4.067952	4.496604	4.965667
CTGF	0.910987	0.810158	1.278854	2.016304	2.410876	2.046384	4.780008	4.905974	4.776362

MDA-MB-2	CYR61	UNKNOWI	SYBR	None				20.265	4.626	0.040503	0.928323
MDA-MB-2	CYR61	UNKNOWI	SYBR	None				20.154	4.670	0.039276	0.900189
MDA-MB-2	CYR61	UNKNOWI	SYBR	None				20.089	4.290	0.051113	1.171488
MDA-MB-2	CYR61	UNKNOWI	SYBR	None				17.491	3.020	0.123253	2.824913
MDA-MB-2	CYR61	UNKNOWI	SYBR	None				17.815	3.199	0.10886	2.495029
MDA-MB-2	CYR61	UNKNOWI	SYBR	None				17.436	2.969	0.127672	2.926187
MDA-MB-2	CYR61	UNKNOWI	SYBR	None				16.497	1.994	0.251005	4.067952
MDA-MB-2	CYR61	UNKNOWI	SYBR	None				16.687	1.968	0.255551	4.496604
MDA-MB-2	CYR61	UNKNOWI	SYBR	None				16.280	1.607	0.328388	4.965667
											0.029322
MDA-MB-2	CTGF	UNKNOWI	SYBR	None				20.866	5.22636795043945	0.026712	0.910987
MDA-MB-2	CTGF	UNKNOWI	SYBR	None				20.879	5.39559459686279	0.023756	0.810158
MDA-MB-2	CTGF	UNKNOWI	SYBR	None				20.535	4.73701858520508	0.037499	1.278854
MDA-MB-2	CTGF	UNKNOWI	SYBR	None				18.551	4.08015727996828	0.059122	2.016304
MDA-MB-2	CTGF	UNKNOWI	SYBR	None				18.437	3.82231311798100	0.070692	2.410876
MDA-MB-2	CTGF	UNKNOWI	SYBR	None				18.526	4.05879402160642	0.060004	2.046384
MDA-MB-2	CTGF	UNKNOWI	SYBR	None				17.338	2.83485755920414	0.14016	4.780008
MDA-MB-2	CTGF	UNKNOWI	SYBR	None				17.516	2.79733085632324	0.143853	4.905974
MDA-MB-2	CTGF	UNKNOWI	SYBR	None				17.509	2.83595848083496	0.140053	4.776362

Figure5H

	Vehicle			ET-1 50 nmol/L			ET-1 100 nmol/L		
CYR61	0.960181	0.96118	1.078639	2.019526	2.383832	1.837558	4.518662	3.24321	4.250251
CTGF	1.01361	0.813258	1.173131	2.220159	2.122423	2.352577	5.134434	5.036568	3.956715

Vehicle	CYR61	UNKNOWI	SYBR	None				20.240	4.751724243164040	0.037118	0.960181
Vehicle	CYR61	UNKNOWI	SYBR	None				20.169	4.750224113464360	0.037157	0.96118
Vehicle	CYR61	UNKNOWI	SYBR	None				20.130	4.583890914916950	0.041698	1.078639
ET-1 50	CYR61	UNKNOWI	SYBR	None				18.783	3.679086112976070	0.07807	2.019526
ET-1 50	CYR61	UNKNOWI	SYBR	None				18.694	3.439820289611780	0.092153	2.383832
ET-1 50	CYR61	UNKNOWI	SYBR	None				18.916	3.815312957763650	0.071036	1.837558
ET-1 100	CYR61	UNKNOWI	SYBR	None				17.841	2.517207145690880	0.174681	4.518662
ET-1 100	CYR61	UNKNOWI	SYBR	None				18.335	2.995680236816420	0.125375	3.24321
ET-1 100	CYR61	UNKNOWI	SYBR	None				17.931	2.605554580688500	0.164305	4.250251
											0.073615
Vehicle	CTGF	UNKNOWI	SYBR	None				19.233	3.74435234069824	0.074617	1.01361
Vehicle	CTGF	UNKNOWI	SYBR	None				19.481	4.06206989288330	0.059868	0.813258
Vehicle	CTGF	UNKNOWI	SYBR	None				19.079	3.53349113464355	0.08636	1.173131
ET-1 50	CTGF	UNKNOWI	SYBR	None				17.717	2.61319255828857	0.163437	2.220159
ET-1 50	CTGF	UNKNOWI	SYBR	None				17.933	2.67814350128174	0.156242	2.122423
ET-1 50	CTGF	UNKNOWI	SYBR	None				17.630	2.52961349487305	0.173185	2.352577
ET-1 100	CTGF	UNKNOWI	SYBR	None				16.727	1.40365028381348	0.377972	5.134434
ET-1 100	CTGF	UNKNOWI	SYBR	None				16.770	1.43141460418701	0.370767	5.036568
ET-1 100	CTGF	UNKNOWI	SYBR	None				17.105	1.77955245971680	0.291274	3.956715

Figure5I

siControl			siEDNRA#1			siEDNRA#2		
0.852144	1.006951	1.140905	0.410424	0.345777	0.302195	0.388127	0.427377	0.317873

Figure5J

DMSO			Atrasentan 2 μmol/L			Atrasentan 4 μmol/L		
0.974201	0.969222	1.056577	0.596209	0.521988	0.493775	0.329708	0.328746	0.27241

Figure5K

Flag			Flag-EDNRA 0.5 μg			Flag-EDNRA 1 μg		
0.953251	0.924362	1.122388	2.198372	2.390456	1.982404	4.177187	4.252832	3.819415

Figure5L

Vehicle			ET-1 50 nmol/L			ET-1 100 nmol/L		
0.937757	0.833966	1.228277	1.963595	2.315528	2.106518	3.729021	4.101989	4.100706

Figure5M

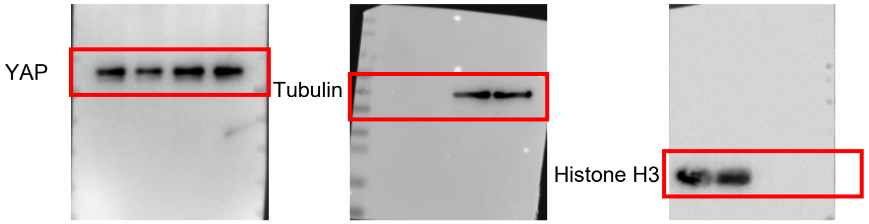

Figure5N

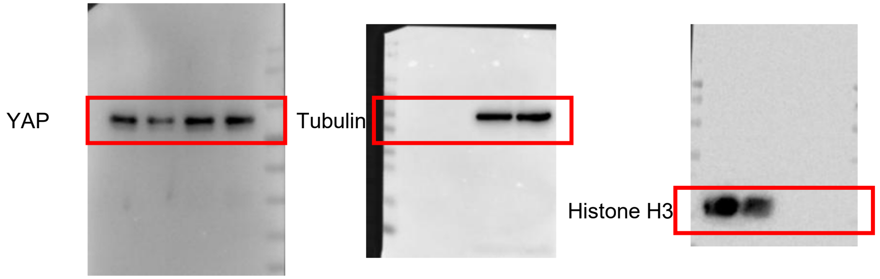

Figure5O

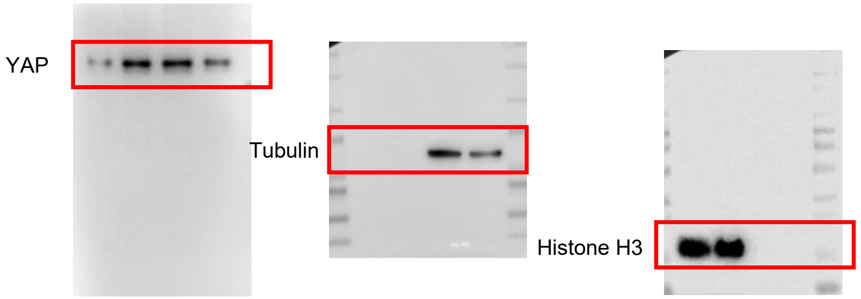

Figure5P

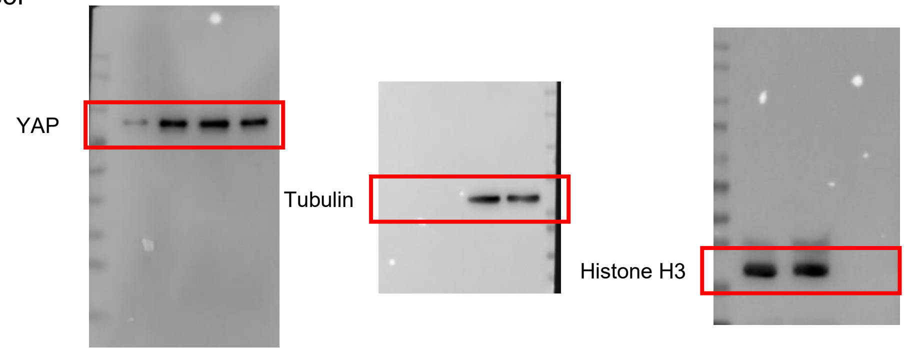

Figure5R

	Cytoplasm			Nucleus		
DMSO	0.471293	0.48523	0.502345	0.528707	0.51477	0.497655
Atrasentan	0.870572	0.903541	0.893624	0.129428	0.096459	0.106377

Figure5T

	Cytoplasm			Nucleus		
Vehicle	0.572353	0.605236	0.593834	0.427647	0.394764	0.406166
ET-1	0.237256	0.205713	0.199652	0.762744	0.794287	0.800348

Figure6A

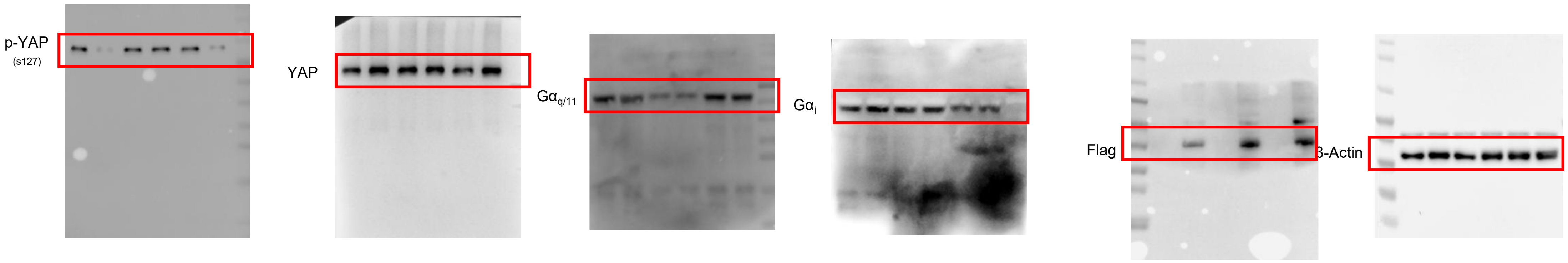

Figure6C

	Cytoplasm			Nucleus		
iControl +	0.653793	0.642562	0.695235	0.346207	0.357438	0.304765
siControl +	0.179548	0.168737	0.207602	0.820452	0.831263	0.792398
siGα$_{q/11}$ +	0.710235	0.752352	0.702256	0.289765	0.247648	0.297744
siGα$_{q/11}$ +	0.735641	0.702315	0.739827	0.26436	0.297685	0.260173
siGα$_i$ + Fla	0.602353	0.592373	0.502353	0.397647	0.407627	0.497647
siGα$_i$ + ED	0.175256	0.187269	0.196317	0.824744	0.812731	0.803683

Figure6D

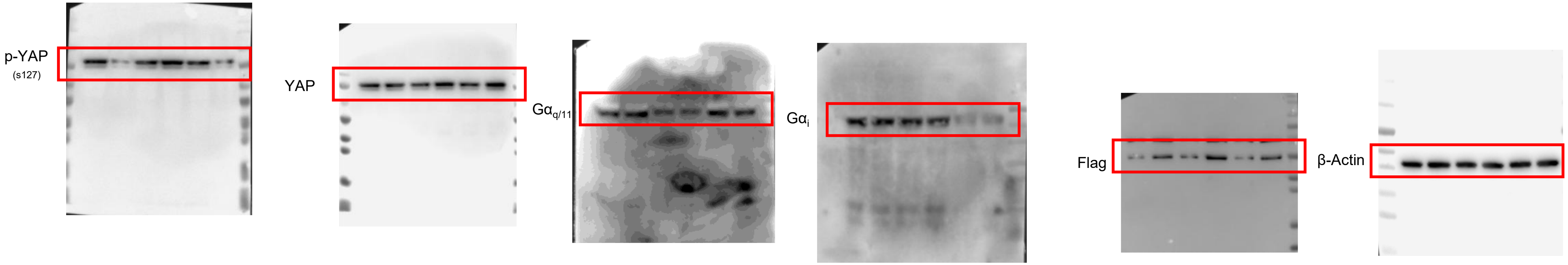

Figure6F

	Cytoplasm			Nucleus		
iControl +	0.672342	0.658219	0.68112	0.327658	0.341781	0.31888
siControl +	0.192385	0.184729	0.205634	0.807615	0.815271	0.794366
siGα$_{q/11}$ +	0.724591	0.741284	0.710295	0.275409	0.258716	0.289706
siGα$_{q/11}$ +	0.75124	0.723481	0.768213	0.24876	0.276519	0.231787
siGα$_i$ + DM	0.584322	0.562135	0.541298	0.415678	0.437865	0.458702
siGα$_i$ + ET	0.185623	0.194313	0.210235	0.814377	0.805687	0.789766

Figure6G

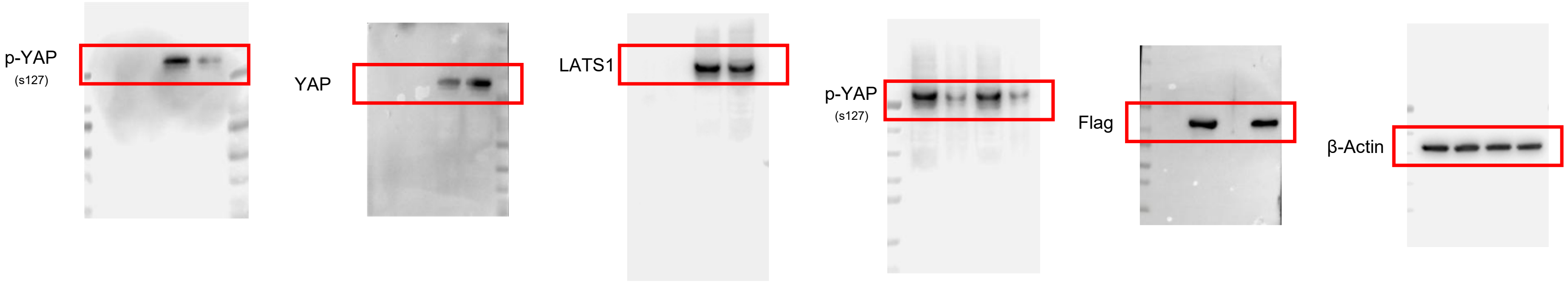

Figure6H

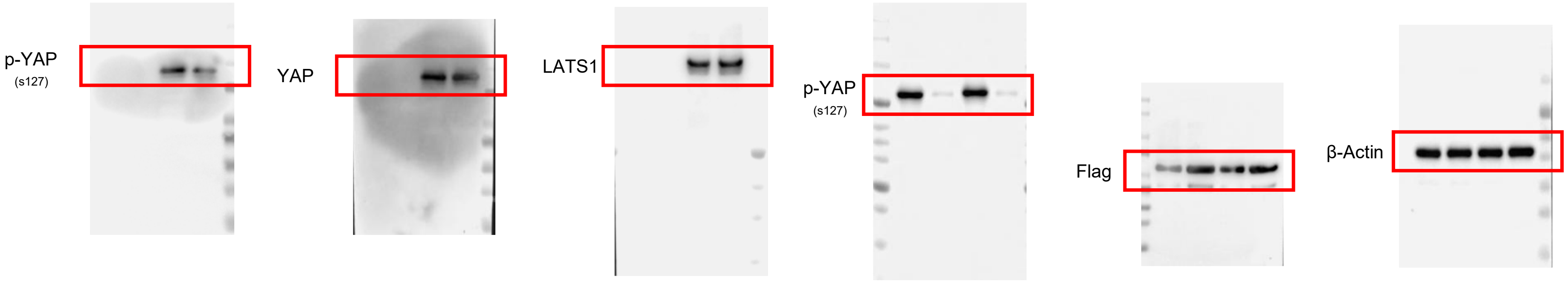

Figure6I

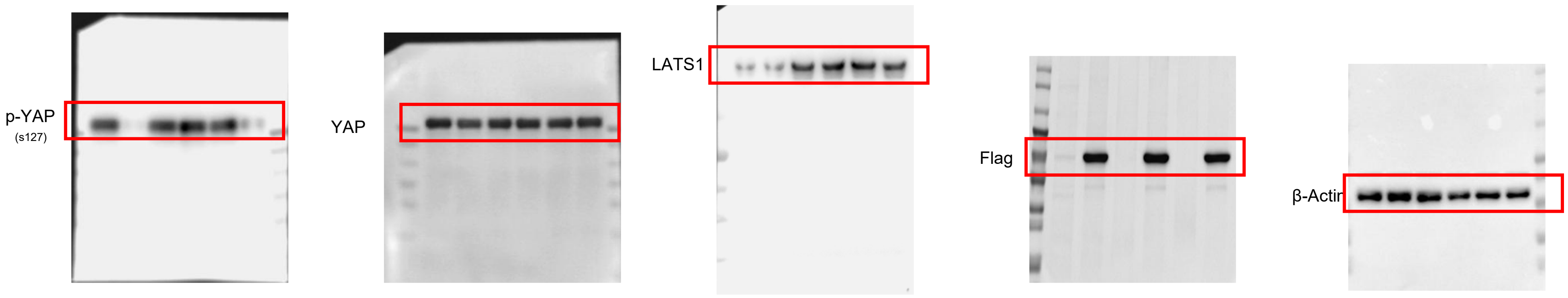

Figure6J

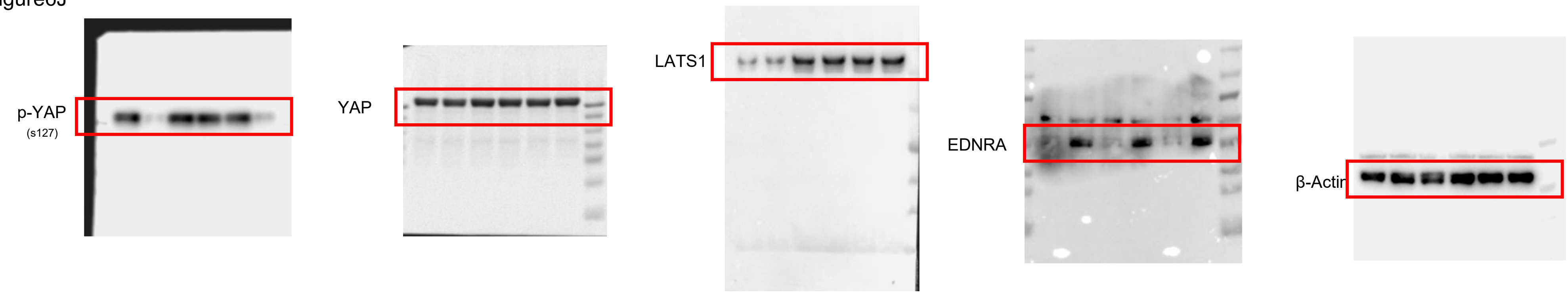

Figure6K

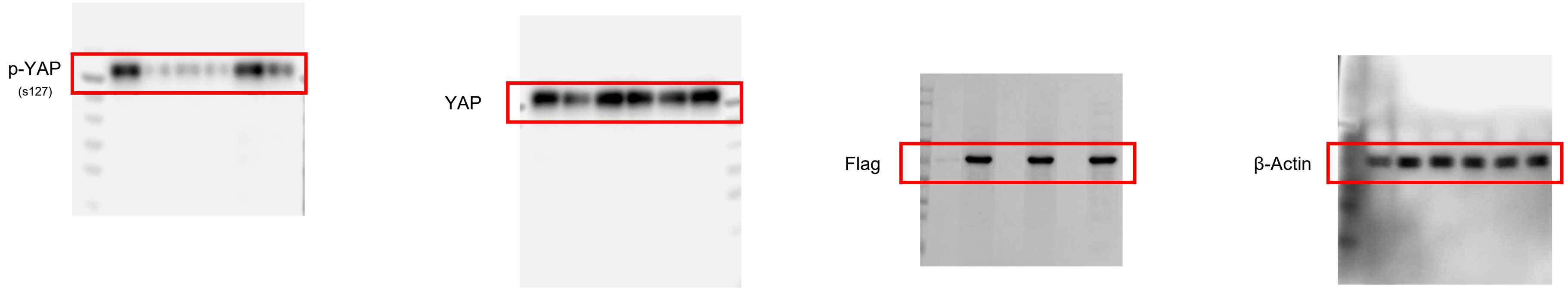

Figure6L

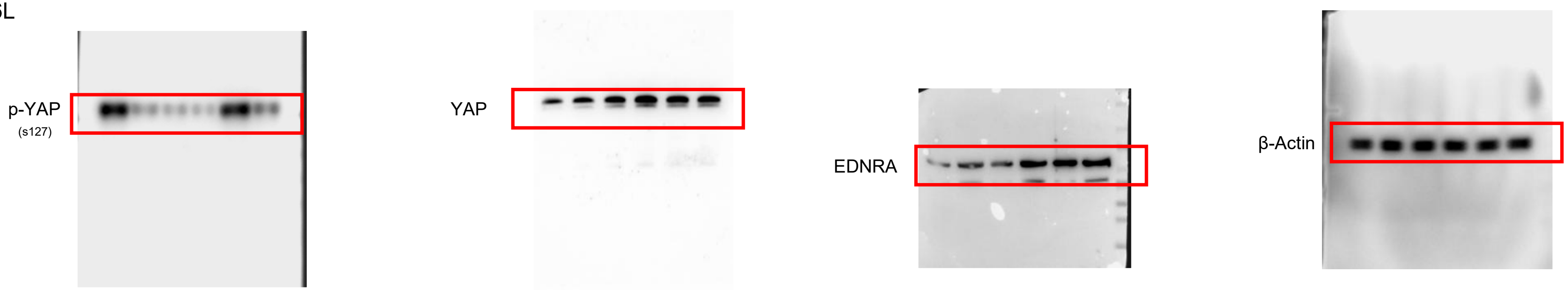

Figure6M

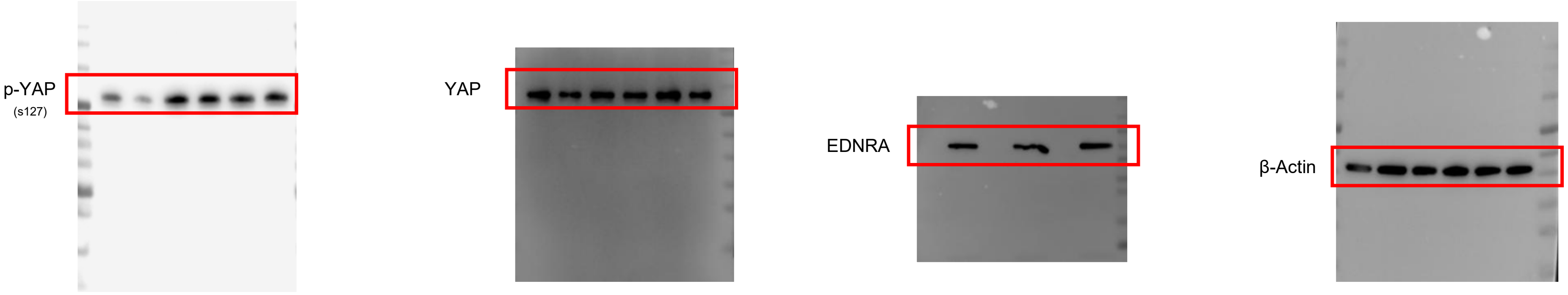

Figure6N

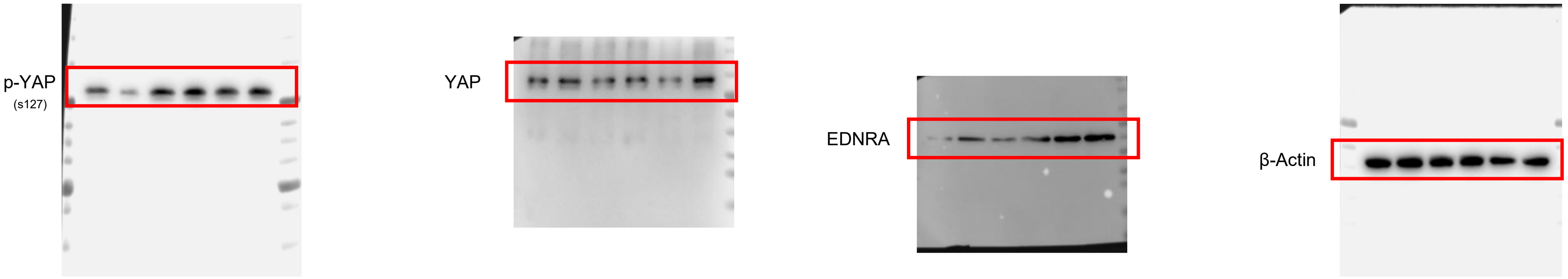

Figure7C

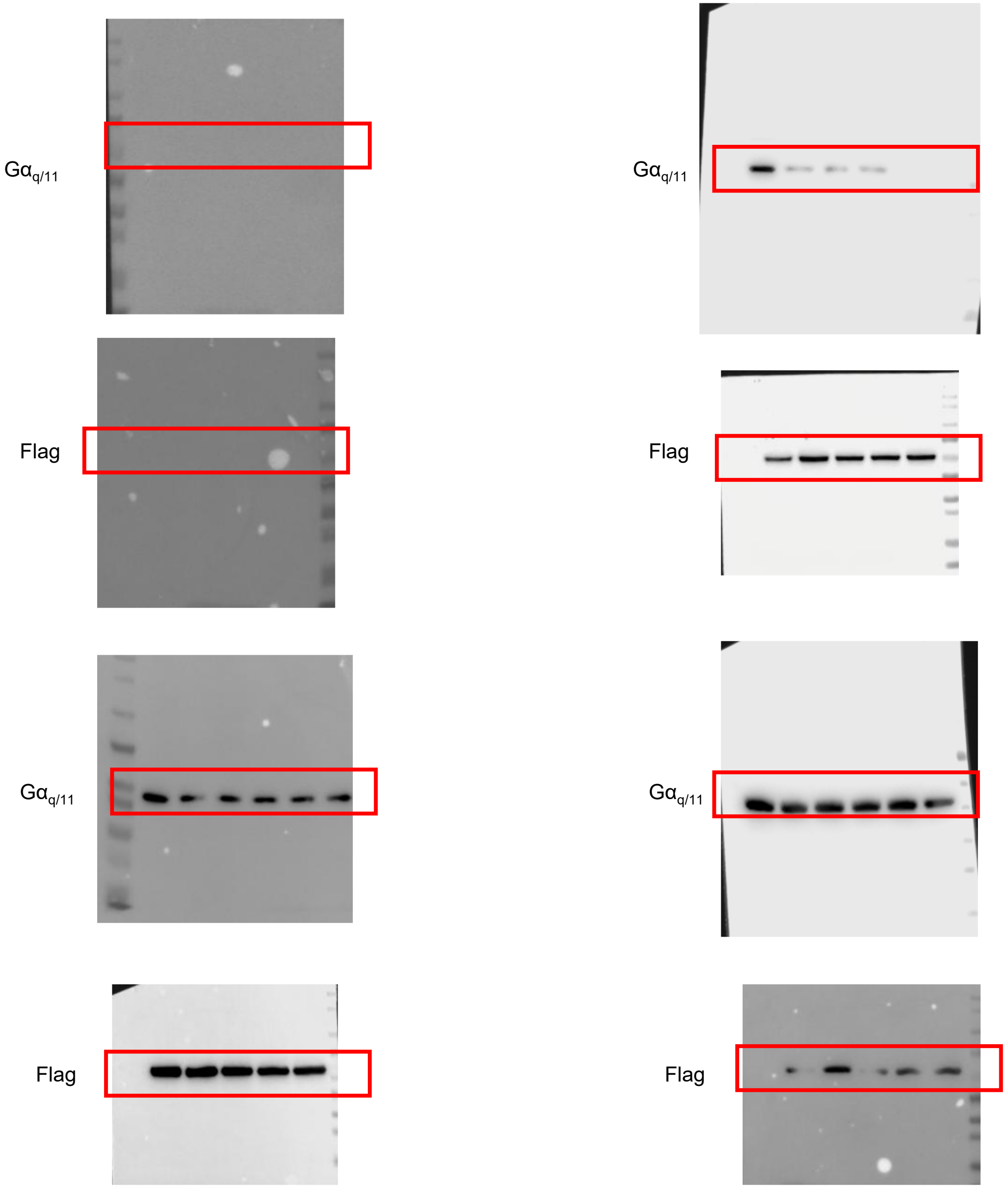
$G\alpha_{q/11}$
$G\alpha_{q/11}$
Flag
Flag
$G\alpha_{q/11}$
$G\alpha_{q/11}$
Flag
Flag

Figure7D

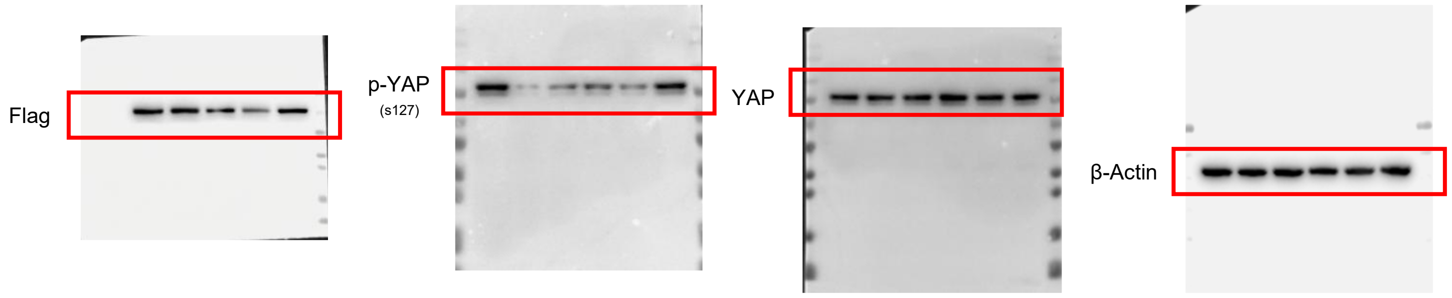

Figure7F

	Cytoplasm			Nucleus		
Flag	0.702453	0.742562	0.765287	0.297548	0.257438	0.234713
WT	0.201748	0.236821	0.194673	0.798252	0.763179	0.805328
Mutant1	0.597428	0.559754	0.576319	0.402573	0.440246	0.423681
Mutant2	0.523605	0.501788	0.538096	0.476395	0.498212	0.461904
Mutant3	0.541454	0.497865	0.512865	0.458546	0.502136	0.487135
Mutant4	0.721379	0.734329	0.714876	0.278621	0.265671	0.285124

Figure7G

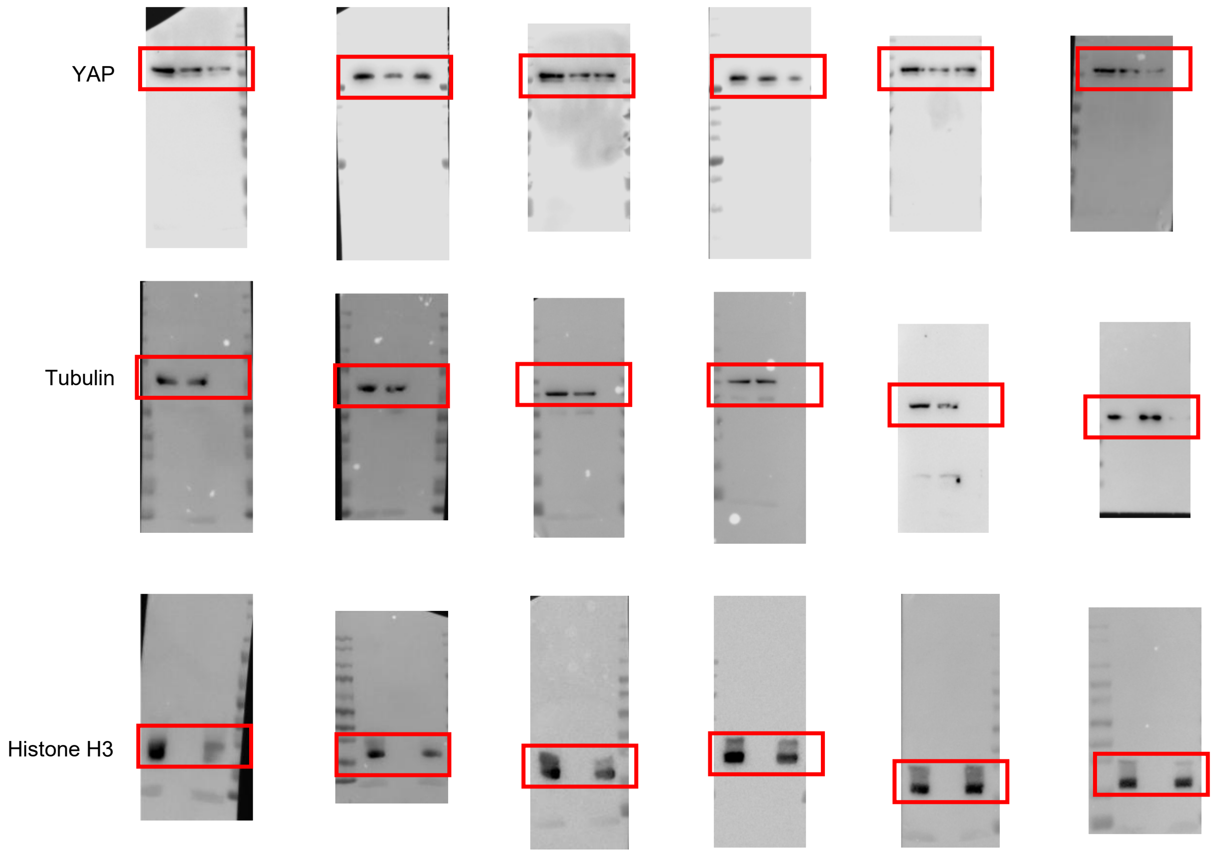

Figure7H

	Flag			EDNRA WT			EDNRA Mutant1			EDNRA Mutant2			EDNRA Mutant3			EDNRA Mutant4		
CYR61	0.897842	1.109965	0.992192	3.244299	3.75633	3.262233	1.741677	1.990452	1.94625	1.575137	1.740003	1.662895	2.328623	1.942744	1.991932	0.891641	1.212876	1.040078
CTGF	0.88813	1.072165	1.039706	3.804735	4.073762	3.888649	1.92621	1.80461	1.6179	1.442771	1.625098	1.598119	2.41596	2.013013	1.921275	0.832365	1.282765	1.104421

A1	FALSE	flag	CYR61	UNKNOW	SYBR	None				18.589	2.56533258056641	0.16895	0.89784
A2	FALSE	flag	CYR61	UNKNOW	SYBR	None				18.574	2.25935226440430	0.20887	1.10997
A3	FALSE	flag	CYR61	UNKNOW	SYBR	None				18.608	2.42117527770996	0.1867	0.99219
A4	FALSE	wt	CYR61	UNKNOW	SYBR	None				17.254	0.71196003723140	0.61049	3.2443
A5	FALSE	wt	CYR61	UNKNOW	SYBR	None				16.904	0.50054309082030	0.70684	3.75633
A6	FALSE	wt	CYR61	UNKNOW	SYBR	None				16.980	0.70400717163090	0.61386	3.26223
A7	FALSE	mutant1	CYR61	UNKNOW	SYBR	None				17.668	1.60938989257800	0.32774	1.74168
A8	FALSE	mutant1	CYR61	UNKNOW	SYBR	None				17.748	1.41677107238770	0.37455	1.99045
A9	FALSE	mutant1	CYR61	UNKNOW	SYBR	None				17.597	1.44916987609860	0.36623	1.94625
A1	FALSE	mutant2	CYR61	UNKNOW	SYBR	None				17.778	1.75438989257800	0.2964	1.57514
A2	FALSE	mutant2	CYR61	UNKNOW	SYBR	None				17.926	1.61077710723870	0.32742	1.74
A3	FALSE	mutant2	CYR61	UNKNOW	SYBR	None				17.863	1.67616987609860	0.31291	1.66289
A4	FALSE	mutant3	CYR61	UNKNOW	SYBR	None				17.732	1.19038989257800	0.43818	2.32862
A5	FALSE	mutant3	CYR61	UNKNOW	SYBR	None				17.855	1.45177107238770	0.36557	1.94274
A6	FALSE	mutant3	CYR61	UNKNOW	SYBR	None				17.692	1.41569876098600	0.37483	1.99193
A7	FALSE	mutant4	CYR61	UNKNOW	SYBR	None				18.634	2.57533258056640	0.16778	0.89164
A8	FALSE	mutant4	CYR61	UNKNOW	SYBR	None				18.462	2.13143522644040	0.22823	1.21288
A9	FALSE	mutant4	CYR61	UNKNOW	SYBR	None				18.501	2.35317527771000	0.19571	1.04008
													0.04898
B1	FALSE	flag	CTGF	UNKNOW	SYBR	None				20.547	4.52287881469726	0.0435	0.88813
B2	FALSE	flag	CTGF	UNKNOW	SYBR	None				20.566	4.25119453430176	0.05251	1.07216
B3	FALSE	flag	CTGF	UNKNOW	SYBR	None				20.483	4.29554585266113	0.05092	1.03971
B4	FALSE	wt	CTGF	UNKNOW	SYBR	None				18.966	2.42392521667480	0.18635	3.80473
B5	FALSE	wt	CTGF	UNKNOW	SYBR	None				18.728	2.32535922241210	0.19952	4.07376
B6	FALSE	wt	CTGF	UNKNOW	SYBR	None				18.668	2.39245207214360	0.19046	3.88865
B7	FALSE	mutant1	CTGF	UNKNOW	SYBR	None				19.465	3.40595628356930	0.09434	1.92621
B8	FALSE	mutant1	CTGF	UNKNOW	SYBR	None				19.831	3.50003370666500	0.08839	1.80461
B9	FALSE	mutant1	CTGF	UNKNOW	SYBR	None				19.806	3.65759844970700	0.07924	1.6179
B1	FALSE	mutant2	CTGF	UNKNOW	SYBR	None				19.847	3.82287881469730	0.07066	1.44277
B2	FALSE	mutant2	CTGF	UNKNOW	SYBR	None				19.966	3.65119453430180	0.07959	1.6251
B3	FALSE	mutant2	CTGF	UNKNOW	SYBR	None				19.862	3.67534623000000	0.07827	1.59812
B4	FALSE	mutant3	CTGF	UNKNOW	SYBR	None				19.621	3.07912456930000	0.11833	2.41596
B5	FALSE	mutant3	CTGF	UNKNOW	SYBR	None				19.745	3.34236452346650	0.09859	2.01301
B6	FALSE	mutant3	CTGF	UNKNOW	SYBR	None				19.686	3.40965684497070	0.0941	1.92128
B7	FALSE	mutant4	CTGF	UNKNOW	SYBR	None				20.675	4.61643240000000	0.04077	0.83237
B8	FALSE	mutant4	CTGF	UNKNOW	SYBR	None				20.323	3.99246456000000	0.06283	1.28276
B9	FALSE	mutant4	CTGF	UNKNOW	SYBR	None				20.356	4.20843123000000	0.05409	1.10442

Figure7J

	Cytoplasm			Nucleus		
Flag	0.702453	0.742562	0.765287	0.297548	0.257438	0.234713
WT	0.201748	0.236821	0.194673	0.798252	0.763179	0.805328
Mutant1	0.597428	0.559754	0.576319	0.402573	0.440246	0.423681
Mutant2	0.523605	0.501788	0.538096	0.476395	0.498212	0.461904
Mutant3	0.541454	0.497865	0.512865	0.458546	0.502136	0.487135
Mutant4	0.721379	0.734329	0.714876	0.278621	0.265671	0.285124

Figure7K

Flag			EDNRA WT			EDNRA Mutant1			EDNRA Mutant2			EDNRA Mutant3			EDNRA Mutant4		
1.02151	1.13266	0.84582	3.25214	2.98654	3.10512	1.45214	1.62385	1.63421	1.36214	1.49124	1.29654	1.61321	1.4978	1.74235	1.20512	1.09872	1.19022

Figure7M

Flag			EDNRA WT			EDNRA Mutant1			EDNRA Mutant2			EDNRA Mutant3			EDNRA Mutant4		
1.18246	0.92341	0.89413	3.12565	2.54891	2.72546	1.62451	1.65643	1.71895	1.58421	1.35247	1.56321	1.80945	1.91256	1.69784	1.05643	1.21457	1.02895

Figure7O

Flag			EDNRA WT			EDNRA Mutant1			EDNRA Mutant2			EDNRA Mutant3			EDNRA Mutant4		
0.30842	0.35123	0.32035	0.88246	0.7641	0.81325	0.44211	0.39854	0.47931	0.42543	0.51895	0.47562	0.61562	0.52451	0.63985	0.38432	0.34126	0.37449

Figure8C

sgCtrl			sg1		
1.14286	0.97654	0.91246	0.54286	0.46321	0.41235

sgCtrl			sg2		
1.08523	1.02346	0.89412	0.44232	0.49567	0.47235

Figure8D

sgCtrl			sg1			sg2		
1.12457	0.93564	0.94123	0.94123	1.02564	0.98453	0.42564	0.54321	0.49123

Figure8E

	siControl			siYAP#1			siYAP#2		
CYR61	1.09158	0.8647	1.04372	0.30666	0.31381	0.38213	0.35397	0.38382	0.35322
CTGF	1.0502	0.83389	1.11591	0.44935	0.41957	0.45341	0.37797	0.38306	0.32975

FALSE	siC	CYR61	UNKNOW	SYBR	None				20.7245	4.57027	0.042093171	1.091577806
FALSE	siC	CYR61	UNKNOW	SYBR	None				20.89312	4.90641	0.033344439	0.864702022
FALSE	siC	CYR61	UNKNOW	SYBR	None				20.6584	4.63495	0.040247696	1.043720172
FALSE	siYAP	CYR61	UNKNOW	SYBR	None				22.6109	6.40198	0.011825295	0.306658526
FALSE	siYAP	CYR61	UNKNOW	SYBR	None				22.54327	6.36871	0.012101167	0.313812553
FALSE	siYAP	CYR61	UNKNOW	SYBR	None				22.3256	6.08457	0.0147354	0.38212458
FALSE	siYAP2	CYR61	UNKNOW	SYBR	None				22.3841	6.19498	0.013649766	0.353971465
FALSE	siYAP2	CYR61	UNKNOW	SYBR	None				22.22349	6.07818	0.014800811	0.383820844
FALSE	siYAP2	CYR61	UNKNOW	SYBR	None				22.2105	6.19803	0.013620939	0.353223924
												0.158049219
FALSE	siC	CTGF	UNKNOW	SYBR	None				18.74512	2.59089	0.1659833	1.050200063
FALSE	siC	CTGF	UNKNOW	SYBR	None				18.91034	2.92363	0.131795224	0.833887222
FALSE	siC	CTGF	UNKNOW	SYBR	None				18.52678	2.50333	0.176369133	1.115912715
FALSE	siYAP	CTGF	UNKNOW	SYBR	None				20.02456	3.81564	0.071019549	0.449350835
FALSE	siYAP	CTGF	UNKNOW	SYBR	None				20.08912	3.91456	0.066313205	0.419573128
FALSE	siYAP	CTGF	UNKNOW	SYBR	None				20.04371	3.80268	0.071660405	0.453405623
FALSE	siYAP2	CTGF	UNKNOW	SYBR	None				20.25432	4.0652	0.0597383	0.377972764
FALSE	siYAP2	CTGF	UNKNOW	SYBR	None				20.19123	4.04592	0.060541994	0.383057849
FALSE	siYAP2	CTGF	UNKNOW	SYBR	None				20.27456	4.26209	0.052117439	0.329754485

Figure8F

	DMSO			VP 2 μmol/L			VP 4 μmol/L		
CYR61	1.09184	0.87459	1.02863	0.42077	0.45956	0.42503	0.317	0.31924	0.26548
CTGF	1.06868	0.86057	1.10672	0.45668	0.43272	0.4527	0.30856	0.27326	0.26773

FALSE	0	CYR61	UNKNOW	SYBR	None				20.6984	4.56993	0.042103092	1.091835089
FALSE	0	CYR61	UNKNOW	SYBR	None				20.85412	4.89	0.033725882	0.874593764
FALSE	0	CYR61	UNKNOW	SYBR	None				20.7123	4.65596	0.039665815	1.028630593
FALSE	2	CYR61	UNKNOW	SYBR	None				22.1891	5.94559	0.016225537	0.420767438
FALSE	2	CYR61	UNKNOW	SYBR	None				22.01043	5.81835	0.017721567	0.459563132
FALSE	2	CYR61	UNKNOW	SYBR	None				22.1456	5.93104	0.016390004	0.425032471
FALSE	4	CYR61	UNKNOW	SYBR	None				22.5109	6.35412	0.012224167	0.317002246
FALSE	4	CYR61	UNKNOW	SYBR	None				22.45641	6.34398	0.012310388	0.319238149
FALSE	4	CYR61	UNKNOW	SYBR	None				22.6345	6.60999	0.010237519	0.265483659
												0.159944155
FALSE	0	CTGF	UNKNOW	SYBR	None				18.6942	2.56573	0.168903368	1.06867575
FALSE	0	CTGF	UNKNOW	SYBR	None				18.84231	2.87819	0.136012391	0.860569841
FALSE	0	CTGF	UNKNOW	SYBR	None				18.5716	2.51526	0.174916707	1.106723007
FALSE	2	CTGF	UNKNOW	SYBR	None				20.0358	3.79229	0.072178351	0.456682745
FALSE	2	CTGF	UNKNOW	SYBR	None				20.06214	3.87006	0.068390512	0.432716544
FALSE	2	CTGF	UNKNOW	SYBR	None				20.0195	3.80494	0.071548236	0.452695913
FALSE	4	CTGF	UNKNOW	SYBR	None				20.5147	4.35792	0.048768047	0.308562402
FALSE	4	CTGF	UNKNOW	SYBR	None				20.64563	4.5332	0.043188769	0.273261513
FALSE	4	CTGF	UNKNOW	SYBR	None				20.5872	4.56269	0.042314913	0.267732501

Figure8G

	DMSO			XMU-MP-1 2 μmol/L			XMU-MP-1 4 μmol/L		
CYR61	1.00092	0.91294	1.08614	1.81951	1.94553	1.89307	3.34243	3.61721	3.69652
CTGF	1.03106	0.96288	1.00606	1.90772	1.79963	2.06627	3.74613	4.08943	3.7349

FALSE	0	CYR61	UNKNOW	SYBR	None				22.6412	6.49793	0.011064407	1.000919859
FALSE	0	CYR61	UNKNOW	SYBR	None				22.58347	6.63066	0.010091889	0.912942857
FALSE	0	CYR61	UNKNOW	SYBR	None				22.4195	6.38005	0.012006421	1.086137284
FALSE	2	CYR61	UNKNOW	SYBR	None				21.8568	5.63571	0.02011325	1.819505568
FALSE	2	CYR61	UNKNOW	SYBR	None				21.72341	5.53909	0.021506402	1.945534369
FALSE	2	CYR61	UNKNOW	SYBR	None				21.7842	5.57853	0.02092643	1.893068304
FALSE	4	CYR61	UNKNOW	SYBR	None				20.8912	4.75836	0.036947998	3.342427961
FALSE	4	CYR61	UNKNOW	SYBR	None				20.74563	4.64438	0.03998548	3.617207766
FALSE	4	CYR61	UNKNOW	SYBR	None				20.6587	4.61309	0.04086218	3.69651679
												0.041666714
FALSE	0	CTGF	UNKNOW	SYBR	None				20.6841	4.54083	0.042960959	1.031061846
FALSE	0	CTGF	UNKNOW	SYBR	None				20.59234	4.63953	0.040120127	0.962881972
FALSE	0	CTGF	UNKNOW	SYBR	None				20.6157	4.57625	0.041919055	1.006056182
FALSE	2	CTGF	UNKNOW	SYBR	None				19.8742	3.65311	0.079488502	1.907721895
FALSE	2	CTGF	UNKNOW	SYBR	None				19.92158	3.73726	0.074984697	1.799630686
FALSE	2	CTGF	UNKNOW	SYBR	None				19.7436	3.53793	0.086094805	2.066272991
FALSE	4	CTGF	UNKNOW	SYBR	None				18.8124	2.67956	0.156088916	3.746129758
FALSE	4	CTGF	UNKNOW	SYBR	None				18.65431	2.55306	0.170393239	4.089433127
FALSE	4	CTGF	UNKNOW	SYBR	None				18.7295	2.68389	0.155621144	3.734903252

Figure8H

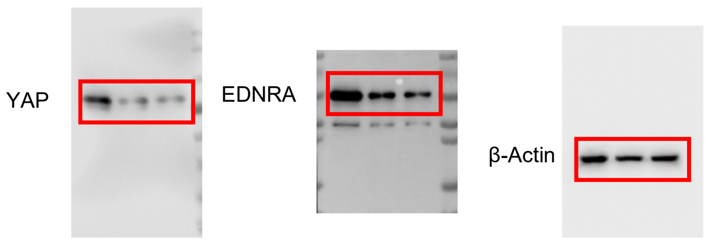

Figure8I

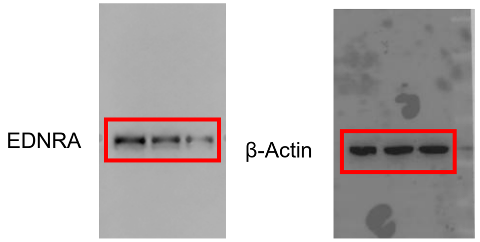

Figure8J

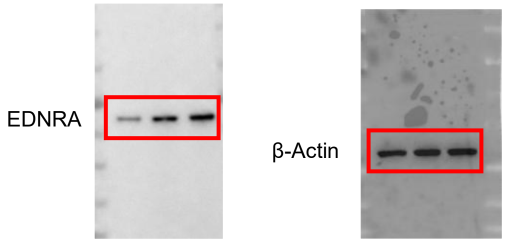

Figure8K

	siControl			siYAP#1			siYAP#2		
EDNRA	1.084357	0.973296	0.942347	0.570837	0.450563	0.458995	0.320619	0.331585	0.326264

FALSE	sic	EDNRA	UNKNOW	SYBR	None				23.85641	7.73185	6.58423E-08	1.084357293
FALSE	sic	EDNRA	UNKNOW	SYBR	None				24.0123	8.04358	5.90986E-08	0.973295776
FALSE	sic	EDNRA	UNKNOW	SYBR	None				24.05892	8.00658	5.72194E-08	0.942346931
FALSE	siYAP1	EDNRA	UNKNOW	SYBR	None				24.7821	8.56621	3.46613E-08	0.57083678
FALSE	siYAP1	EDNRA	UNKNOW	SYBR	None				25.12345	8.92913	2.73582E-08	0.450562828
FALSE	siYAP1	EDNRA	UNKNOW	SYBR	None				25.0967	8.88999	2.78702E-08	0.458994954
FALSE	siYAP2	EDNRA	UNKNOW	SYBR	None				25.61432	9.47197	1.9468E-08	0.320618638
FALSE	siYAP2	EDNRA	UNKNOW	SYBR	None				25.5658	9.45556	2.01339E-08	0.331584896
FALSE	siYAP2	EDNRA	UNKNOW	SYBR	None				25.58914	9.55057	1.98108E-08	0.326263657

Figure8L

	DMSO			VP 2 μmol/L			VP 4 μmol/L		
EDNRA	1.11097	0.990171	0.914408	0.558973	0.45615	0.444437	0.318433	0.305438	0.299993

FALSE	0	EDNRA	UNKNOW	SYBR	None				23.82143	7.68859	6.74582E-08	1.110970262
FALSE	0	EDNRA	UNKNOW	SYBR	None				23.9875	8.01299	6.01233E-08	0.99017141
FALSE	0	EDNRA	UNKNOW	SYBR	None				24.10234	8.05342	5.5523E-08	0.91440817
FALSE	2	EDNRA	UNKNOW	SYBR	None				24.8124	8.58899	3.39409E-08	0.558972882
FALSE	2	EDNRA	UNKNOW	SYBR	None				25.10567	8.90442	2.76975E-08	0.456149993
FALSE	2	EDNRA	UNKNOW	SYBR	None				25.1432	8.94753	2.69863E-08	0.444436805
FALSE	4	EDNRA	UNKNOW	SYBR	None				25.62419	9.46987	1.93353E-08	0.318432656
FALSE	4	EDNRA	UNKNOW	SYBR	None				25.6843	9.57539	1.85462E-08	0.305437732
FALSE	4	EDNRA	UNKNOW	SYBR	None				25.71025	9.66794	1.82156E-08	0.299992887

Figure8M

	DMSO			XMU-MP-1 2 μmol/L			XMU-MP-1 4 μmol/L		
EDNRA	1.022301	0.968458	1.009241	1.838043	1.913536	2.009032	3.051991	3.314797	3.336026

FALSE	0	EDNRA	UNKNOW	SYBR	None				25.63412	9.5185	0.001363471	1.022301472
FALSE	0	EDNRA	UNKNOW	SYBR	None				25.5789	9.59656	0.001291658	0.96845754
FALSE	0	EDNRA	UNKNOW	SYBR	None				25.60124	9.53705	0.001346052	1.009240987
FALSE	2	EDNRA	UNKNOW	SYBR	None				24.9032	8.67215	0.002451448	1.838043236
FALSE	2	EDNRA	UNKNOW	SYBR	None				24.8015	8.61408	0.002552134	1.91353555
FALSE	2	EDNRA	UNKNOW	SYBR	None				24.7562	8.54382	0.002679501	2.009032223
FALSE	4	EDNRA	UNKNOW	SYBR	None				24.0891	7.94057	0.004070523	3.051990991
FALSE	4	EDNRA	UNKNOW	SYBR	None				23.94237	7.8214	0.004421035	3.314797418
FALSE	4	EDNRA	UNKNOW	SYBR	None				23.8654	7.81219	0.004449349	3.336026395

Figure9A

Concertration of P'		PTX			PTX+Atrasentan		
	0	100.3847	99.61758	100.1289	97.24164	96.48257	95.60383
	5	95.61837	93.84622	94.92756	83.12695	85.42832	84.05167
	10	88.71359	90.94268	89.75644	67.82948	70.18325	69.40762
	15	78.58424	80.2417	79.36272	48.76313	51.48276	49.8316
	20	65.93285	68.43572	66.74929	32.43692	34.78153	33.89427
	25	52.84639	55.17463	53.65392	20.68275	22.91349	21.74954
	30	35.47284	37.91857	36.21466	11.23649	13.8127	12.14594
	35	21.38463	23.65791	22.19355	6.384715	7.526491	6.912837
	40	11.49264	13.26745	12.58473	3.548216	4.673895	4.129582

Figure9H

Concertration of P'		PTX			PTX+Atrasentan		
	0	**99.84262**	**100.5738**	**99.31649**	**96.81427**	**95.92842**	**96.35782**
	5	97.13458	96.25195	95.84274	89.36472	88.15749	87.5362
	10	92.63749	90.84627	91.72854	79.74262	77.46194	78.19872
	15	84.38476	86.19433	85.02865	66.18427	63.84295	64.91864
	20	75.94382	73.86259	74.63219	52.87594	50.48622	51.69453
	25	63.48153	61.23789	62.74632	39.14266	37.58644	38.42891
	30	48.93674	46.18759	47.64382	25.68439	23.87956	24.92715
	35	34.18643	32.67495	33.54828	15.73648	14.38572	15.09284
	40	22.78364	20.94676	21.86592	8.674259	7.498316	7.943582

Figure9G

DMSO			PTX			PTX+Atrasentan		
0.958427	1.047316	1.012584	0.621638	0.643275	0.547491	0.174628	0.213547	0.146382

Figure9H

DMSO			PTX			PTX+Atrasentan		
1.064281	0.947536	1.018724	0.573418	0.642785	0.618253	0.136524	0.172841	0.114637

Figure9K

DMSO			PTX			PTX+Atrasentan		
6.43	5.94	6.56	14.45	12.98	14.74	21.89	21.07	22.66

Figure9L

DMSO			PTX			PTX+Atrasentan		
8.77	8.03	9.21	13.48	14.55	13.79	23.72	24.64	23.28

Figure9N

DMSO			PTX			PTX+Atrasentan		
54.23	50.78	53.64	35.03	37.45	33.21	22.08	25.67	23.01

Figure9P

DMSO			PTX			PTX+Atrasentan		
52.65	57.93	53.88	37.66	33.53	34.92	22.07	25.48	26.19
